# Flavonoids from *Pyrus communis* L. ‘Kashi’: Isolation, Structural Identification, NO Inhibitory Activity, and Molecular Docking

**DOI:** 10.3390/molecules31213859

**Published:** 2026-10-26

**Authors:** Zhuang-Zhuang Tang, Erkin Tursun, Adilijiang Abulimiti, Yu-Jie Qiao, Yu Xue, Xue-Lin Cheng, Maimaiti Tuerxun, Abdulla Yusuf, Adila Abudurexiti

**Affiliations:** Key Laboratory of Xinjiang Native Medicinal and Edible Plant Resources Chemistry, College of Chemistry and Environmental Science, Kashi University, Kashi 844000, China

**Keywords:** *Pyrus communis* L. ‘Kashi’, flavonoids, nitric oxide inhibition, structure–activity relationship, molecular docking

## Abstract

*Pyrus communis* L. ‘Kashi’, a Xinjiang pear cultivar, is traditionally consumed after baking to alleviate inflammation-related ailments, although its active constituents remain unclear. Flavonoids were isolated chromatographically and identified by nuclear magnetic resonance (NMR) spectroscopy and high-resolution electrospray ionization mass spectrometry (HR-ESI-MS). Cytotoxicity and nitric oxide (NO) inhibition were evaluated in lipopolysaccharide (LPS)-stimulated RAW264.7 macrophages using 3-(4,5-dimethylthiazol-2-yl)-2,5-diphenyltetrazolium bromide (MTT) and Griess assays, respectively. Docking was performed with inducible nitric oxide synthase (iNOS) and cyclooxygenase-2 (COX-2). Eleven flavonoids were isolated and identified; four were isolated and NMR-characterized from *Pyrus* for the first time. Cell viability remained above 60% at 100 μM. Apigenin, kaempferol, and isorhamnetin-3-O-galactoside showed the strongest NO-inhibitory effects, with half-maximal inhibitory concentration (IC_50_) values of 22.75 ± 3.23, 37.54 ± 4.40, and 58.19 ± 10.41 μM, respectively. Preliminary structure–activity relationship (SAR) analysis suggested that reduced hydroxylation in compounds **1**–**3** and shorter sugar chains among the glycosides examined tended to be associated with lower normalized NO levels; Isorhamnetin-3-O-galactoside was more active than the corresponding glucoside. Docking revealed differences in predicted ligand orientation and residue interactions, providing complementary qualitative structural information. These findings expand the flavonoid profile of *P. communis* L. ‘Kashi’ and highlight preliminary NO-inhibitory SAR trends.

## 1. Introduction

The genus *Pyrus* L. is widely distributed and has a long history of culinary and medicinal use. In Traditional Chinese Medicine, pears are traditionally used for moistening the lungs, clearing the heart, resolving phlegm, and reducing internal heat [1]. *Pyrus communis* L. ‘Kashi’ (locally called ‘Kuke Amuti’) is a distinct pear cultivar grown primarily in the Kashi region of Xinjiang, China, and represents an important regional food and medicinal resource. Locally, baked pears are traditionally used to relieve cough, chronic bronchitis, and sore throat [2], which has prompted investigation of their potential anti-inflammatory properties. Nevertheless, the chemical constituents associated with the biological activity of *P*. *communis* L. ‘Kashi’, particularly its potentially anti-inflammatory flavonoids and their structure–activity relationships (SAR), remain insufficiently characterized.

Phytochemical studies have shown that *Pyrus* species contain diverse bioactive constituents, including flavonoids, triterpenes, and phenylpropanoids [3,4]. Among them, flavonoids represent an important class of natural products with a broad range of biological activities, including anti-inflammatory effects [5,6]. Flavonoids occur as aglycones or glycosides, and depending on their degree of glycosylation, they are classified as aglycones, flavonoid monoglycosides (FMGs), flavonoid diglycosides (FDGs), and flavonoid triglycosides (FTGs). Differences in glycosylation can significantly affect their physicochemical properties and biological activities [7]. Our previous study on *P. communis* L. ‘Kashi’ showed that the ethyl acetate and n-butanol fractions were rich in flavonoids and inhibited nitric oxide (NO) production in lipopolysaccharide (LPS)-stimulated RAW264.7 cells. These findings provided a basis for further identification of the flavonoid constituents responsible for the observed activity.

Macrophages play an important role in inflammatory responses. Upon stimulation with LPS, RAW264.7 macrophages produce increased levels of inflammatory mediators, including NO, accompanied by changes in inflammation-related enzymes such as inducible nitric oxide synthase (iNOS) and cyclooxygenase-2 (COX-2) [6]. Accordingly, LPS-stimulated RAW264.7 cells are widely used as an in vitro model for evaluating the potential anti-inflammatory activity of natural products [8]. iNOS and COX-2 are also commonly investigated molecular targets in studies of flavonoid-mediated inflammatory responses [9]. Molecular docking can predict plausible ligand–target binding poses and interactions and therefore provide complementary structural information on ligand-target recognition [10].

In the present study, flavonoids were systematically isolated and identified from *P. communis* L. ‘Kashi’, and their inhibitory effects on NO production were evaluated in LPS-stimulated RAW264.7 macrophages. The relationships between structural features and NO-inhibitory activity were examined by comparing structurally related flavonoids, with particular attention to hydroxyl substitution and glycosylation. Molecular docking with iNOS and COX-2 was further employed to explore potential ligand–target interaction patterns as complementary structural information. The present study therefore aimed to characterize the flavonoid constituents of *P. communis* L. ‘Kashi’ and to identify structural features associated with their NO-inhibitory activity.

## 2. Results and Discussion

### 2.1. Structural Identification of Flavonoids

**Figure 1:**
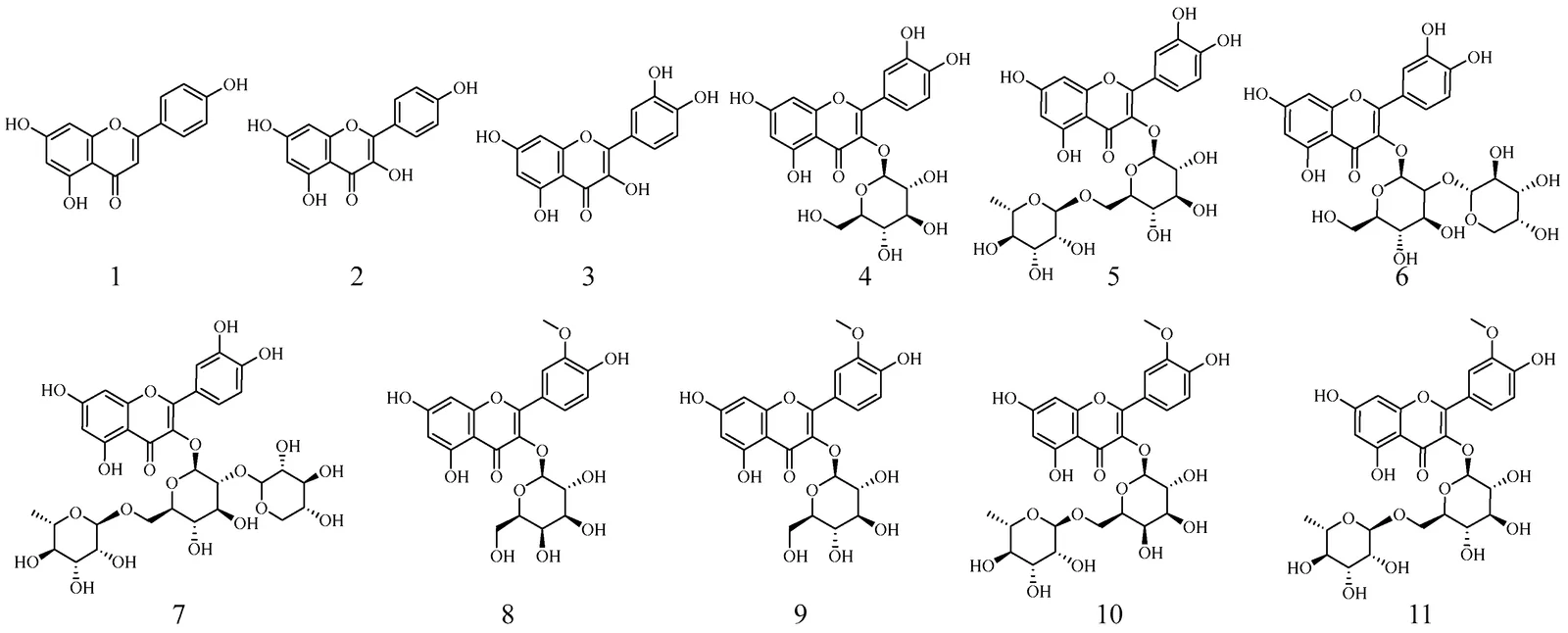
Structures of compounds **1**–**11** isolated from *Pyrus communis* L. ‘Kashi’.

Eleven flavonoids were isolated from *Pyrus communis* ‘Kashi’ and identified (Figure 1). As all compounds are known flavonoids, their structures were confirmed by comparison of their nuclear magnetic resonance (NMR) and high-resolution electrospray ionization mass spectrometry (HR-ESI-MS) data with those reported in the literature. The compounds were: Apigenin (compound **1**) [11], Kaempferol (compound **2**) [11], Quercetin (compound **3**) [11], Isoquercitrin (compound **4**) [12], Rutin (compound **5**) [12], Quercetin-3-O-*L*-arabinopyranosyl-(1 → 2)-*D*-glucopyranoside (compound **6**) [13], Quercetin-3-O-[2-O-*β*-xylopyranosyl-6-O-*α*-rhamnopyranosyl]-*β*-glucopyranoside (compound **7**) [14], Isorhamnetin-3-O-galactoside (compound **8**) [15], Isorhamnetin-3-O-glucoside (compound **9**) [16], Isorhamnetin-3-O-robinobioside (compound **10**) [17], and Narcissoside (compound **11**) [18]. To the best of our knowledge, compound **6** was isolated from the genus *Pyrus* for the first time. Compounds **7**, **8**, and **10** had previously been detected in pear skins by LC-DAD-ESI/MS profiling [19], but had not been isolated and structurally confirmed by NMR. Thus, the present study represents the first isolation and NMR-based structural identification of compounds **7**, **8**, and **10** from the genus *Pyrus*. Detailed NMR spectroscopic data are provided in Supplementary Text S1 and Figures S1–S44.

### 2.2. Cytotoxicity and NO Inhibitory Activity

#### 2.2.1. Cytotoxicity of Flavonoids in RAW264.7 Cells

**Figure 2:**
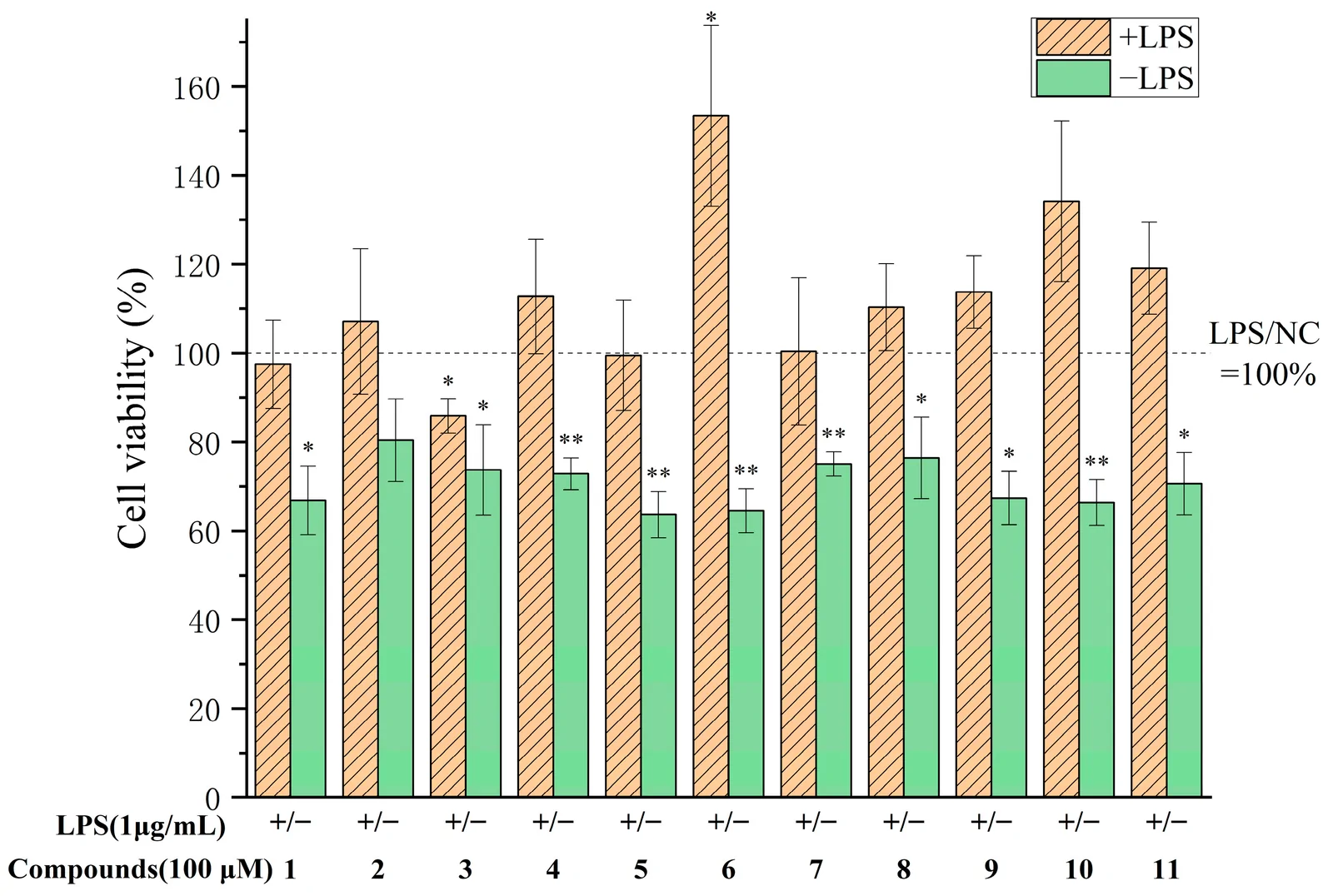
Effect of flavonoids **1**–**11** (100 μM) on the viability of RAW264.7 cells in the presence (+LPS) or absence (−LPS) of LPS (1 μg/mL). Cell viability was calculated relative to the corresponding control (LPS-stimulated model group for +LPS; normal control group for −LPS); the dashed line indicates 100% viability. Data are presented as mean ± SD of three independent experiments (n = 3), each performed in triplicate. Statistical significance was determined by one-way ANOVA followed by Dunnett’s multiple-comparisons test versus the corresponding control (* *p* < 0.05, ** *p* < 0.01).

To evaluate cell viability at the concentration used for subsequent NO screening, the effects of compounds **1**–**11** (100 µM) on RAW264.7 cell viability were evaluated by the MTT assay [20] in the presence (+LPS) or absence (−LPS) of LPS (1 μg/mL) (Figure 2).

Under the −LPS condition, cell viability ranged from 63.65% to 80.36% (Figure 2). Except for compound **2** (80.36 ± 9.31%), all compounds significantly reduced viability relative to the normal control (*p* < 0.05 or *p* < 0.01), with compound **5** showing the lowest value (63.65 ± 5.18%). Under the +LPS condition, viability ranged from 85.87% to 153.39%, with most compounds at or above the LPS control level; notably, compounds **6** (153.39 ± 20.35%) and 10 (134.14 ± 18.03%) showed MTT signals above 100% of the LPS control [21]. Taken together, cell viability remained above 60% at 100 µM under both conditions, although most compounds significantly reduced viability under the −LPS condition.

Because viability remained above 60% for all compounds under both conditions, 100 μM was adopted as the maximum concentration for the subsequent NO inhibitory activity screening.

#### 2.2.2. Inhibitory Effects on NO Production

**Figure 3:**
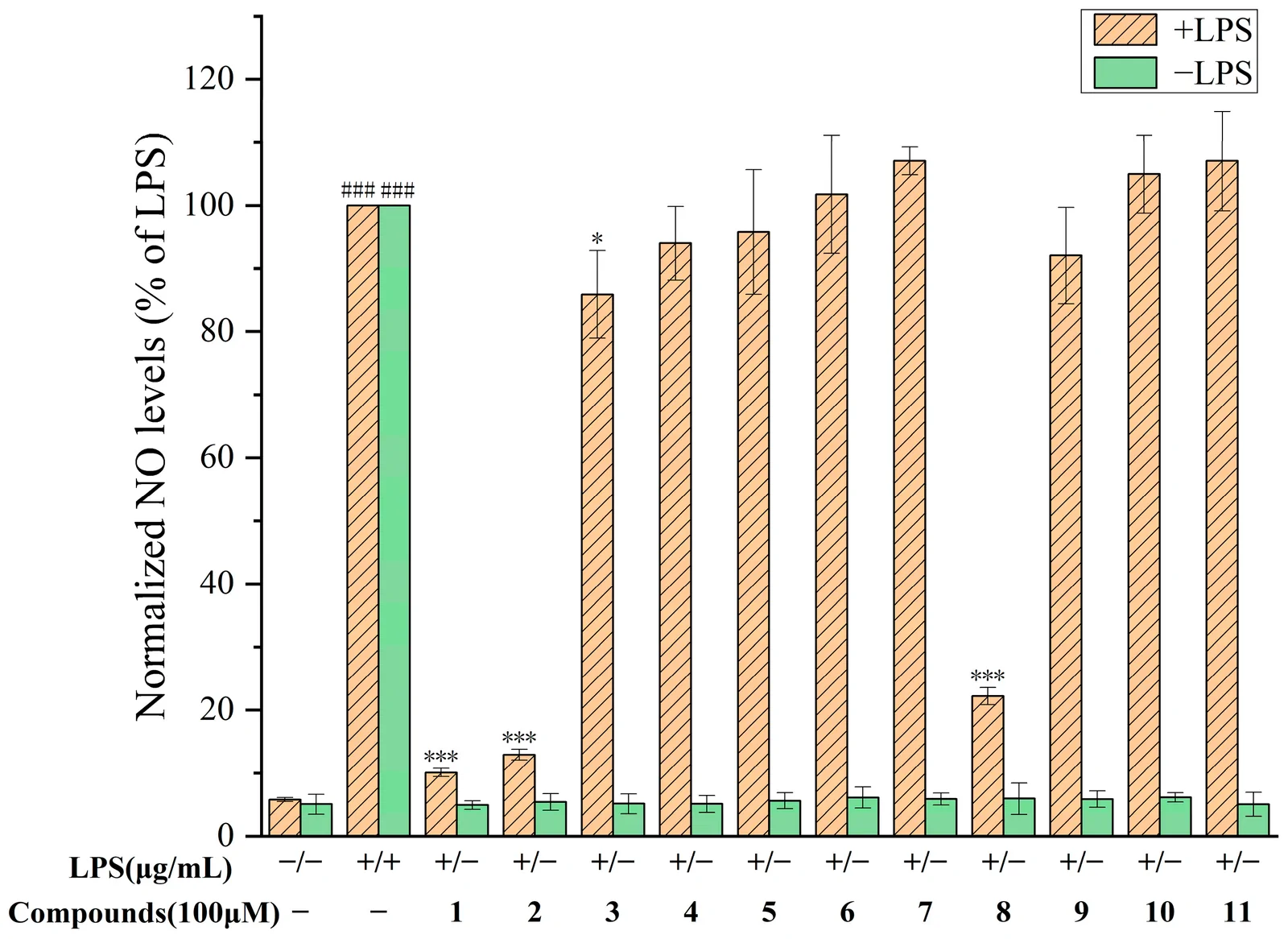
Inhibitory effects of flavonoids **1**–**11** (100 μM) on LPS-induced NO production in RAW264.7 cells. Cells were pretreated with each compound for 2 h and then incubated with or without 1 μg/mL LPS for 16 h (+LPS and −LPS groups, respectively). Normalized NO levels were calculated relative to the LPS-stimulated control (set as 100%, indicated by the dashed line). Data are presented as mean ± SD of three independent experiments (n = 3), each performed in triplicate. Statistical significance was determined by one-way ANOVA followed by Dunnett’s multiple-comparisons test (### *p* < 0.001, LPS group versus NC group; * *p* < 0.05 and *** *p* < 0.001, treatment groups versus LPS group). No significant difference was observed between any −LPS treatment group and the NC group (*p* > 0.05).

The effects of the 11 flavonoids on NO production were evaluated in LPS-stimulated RAW264.7 cells at a screening concentration of 100 μM using the Griess assay. In the +LPS experiment, the unstimulated normal control (NC) showed a basal NO level of only 5.78 ± 0.31% of the LPS control (set as 100%). Compounds **1**, **2**, and **8** exhibited the strongest NO-inhibitory activity, with normalized NO levels of 10.09 ± 0.64%, 12.92 ± 0.87%, and 22.22 ± 1.36%, respectively (all *p* < 0.001 versus the LPS group); compound **3** showed a moderate but significant reduction (85.88 ± 6.96%, *p* < 0.05), whereas the remaining compounds (**4**–**7**, **9**–**11**) showed no significant inhibition (NO levels of 92.06–107.06%, Figure 3). Notably, compounds **6**, **7**, **10**, and **11** showed NO levels slightly above 100%; however, these differences were not statistically significant, indicating no significant inhibitory effect and no significant increase in NO production relative to the LPS group.

To assess whether the compounds altered basal NO levels in the absence of LPS stimulation, NO levels were also measured under LPS-free conditions. In the −LPS experiment, all compounds produced NO levels of 4.92–6.13%, comparable to the NC basal level (5.06 ± 1.55%, *p* > 0.05), supporting the interpretation that the reduced NO levels observed under +LPS conditions reflect inhibition of LPS-induced NO production. A cell-free nitrite/Griess interference control was not performed; therefore, direct chemical interference of the tested compounds with the Griess reaction cannot be completely excluded.

Based on the screening results, compounds **1**, **2**, and **8**, which showed the strongest inhibition at 100 μM, were selected for concentration–response analysis; the remaining compounds were not tested further owing to their insufficient activity at the screening concentration. As shown in Table 1, concentration–response curves were well fitted by nonlinear regression (R^2^ > 0.97), yielding IC_50_ values of 22.75 ± 3.23 μM for apigenin (**1**), 37.54 ± 4.40 μM for kaempferol (**2**), and 58.19 ± 10.41 μM for isorhamnetin-3-O-galactoside (**8**). The corresponding Hill slopes were 1.74 ± 0.34, 2.13 ± 0.91, and 1.57 ± 0.31, respectively. Under the same experimental conditions, the positive control dexamethasone exhibited an IC_50_ value of 7.4 ± 1.3 μM.

To verify that the observed NO inhibition was not attributable to cytotoxicity, the viability of cells treated with compounds **1**, **2**, and **8** (6.25–100 μM) was assessed under both +LPS and −LPS conditions. Viability remained above 70% at all tested concentrations (Figure S111), supporting the interpretation that the observed concentration-dependent reductions in NO were not solely attributable to compound-induced loss of cell viability.

### 2.3. SAR Discussion

Having identified the most active compounds (**1**, **2**, and **8**) for IC_50_ determination in Section 2.2.2, we next performed a preliminary SAR analysis based on the single-concentration NO screening data for all 11 flavonoids. The analysis focused on two structural features: hydroxyl substitution of the flavonoid aglycone and the type and length of the attached sugar moieties. The corresponding physicochemical properties and normalized NO levels are summarized in Table 2.

Comparison of the physicochemical parameters in Table 2 showed that the more active flavonoid aglycones generally had higher cLogP values and lower TPSA values, whereas glycosylation was associated with lower cLogP and higher TPSA values. Because cellular permeability was not directly measured in this study, these parameters are considered physicochemical descriptors associated with the observed activity patterns rather than direct evidence of cellular uptake.

#### 2.3.1. Effect of Hydroxyl Position on the NO-Inhibitory Activity of Flavonoid Aglycones

Among the three structurally related aglycones examined, NO-inhibitory activity varied markedly with the hydroxylation pattern. In particular, the activity data suggested that the absence of hydroxyl groups at C-3′ of the B ring and C-3 of the C ring was associated with lower normalized NO levels.

Quercetin (compound **3**), kaempferol (compound **2**), and apigenin (compound **1**) share a closely related flavonoid scaffold and differ sequentially in the presence or absence of hydroxyl groups at C-3′ and C-3. The corresponding normalized NO levels were 85.88%, 12.92%, and 10.09% of the LPS-stimulated control, respectively. The normalized NO level decreased from 85.88% for quercetin to 12.92% for kaempferol, which lacks the C-3′ hydroxyl group. Apigenin, which additionally lacks the C-3 hydroxyl group, showed a normalized NO level of 10.09%, approximately 2.83 percentage points lower than that of kaempferol.

This trend is consistent with previous reports indicating that the position of hydroxyl substitution can influence flavonoid anti-inflammatory activity. Wang et al. reported that hydroxyl groups at C-5 and C-4′ may favor activity, whereas hydroxylation at C-3′ may reduce it [9]. Zheng et al. similarly reported that hydroxylation at C-3 of the C ring was associated with reduced inhibition of M1 polarization in RAW264.7 macrophages [22]. Within compounds **1**–**3**, decreasing hydroxylation also coincided with higher cLogP and lower TPSA values (Table 2). These physicochemical differences may contribute to the observed cellular activity pattern, but they do not establish a causal mechanism.

Taken together, compounds **1**–**3** support a preliminary SAR observation, restricted to the aglycones examined in this study, in which the absence of hydroxyl groups at C-3′ of the B ring and C-3 of the C ring is associated with stronger NO-inhibitory activity. These results should not be generalized to flavonoids as a whole.

#### 2.3.2. Effects of Sugar Type and Sugar-Chain Length on NO-Inhibitory Activity

Among the glycosylated flavonoids examined, sugar-chain length appeared to be associated with differences in normalized NO levels. In both the quercetin and isorhamnetin series, monoglycosides tended to show lower normalized NO levels than the corresponding di- or triglycosides.

Among the quercetin derivatives, the FTG compound **7** and FDG compounds **5** and **6** showed normalized NO levels of 107.06%, 95.78%, and 101.76% of the LPS-stimulated control, respectively, whereas the FMG compound **4** showed a normalized NO level of 94.01%. In the isorhamnetin series, the FDG compounds **10** and **11** showed normalized NO levels of 104.95% and 107.05%, respectively, whereas the FMGs **8** and **9** showed normalized NO levels of 22.22% and 92.06% of the LPS-stimulated control. These comparisons suggest that, within the compounds tested here, shorter sugar chains tended to be associated with lower normalized NO levels. This observation is consistent with previous reports that extension of the sugar moiety can reduce the cellular activity of flavonoid glycosides [23]. The accompanying increase in cLogP and decrease in TPSA with shorter sugar chains may also contribute to this trend, although these physicochemical parameters alone cannot explain the cellular activity differences.

Sugar configuration also appeared to influence activity at the monoglycoside level. Isorhamnetin-3-O-galactoside (compound **8**) and isorhamnetin-3-O-glucoside (compound **9**) share the same aglycone and contain a single hexose residue, but differ in the configuration at C-4′′ of the sugar moiety. Their normalized NO levels were 22.22% and 92.06% of the LPS-stimulated control, respectively. This matched comparison suggests that sugar configuration may contribute to the pronounced activity difference between compounds **8** and **9**.

However, this trend was not maintained at the diglycoside level. Compound **10**, which contains galactose as part of its disaccharide moiety, showed a normalized NO level of 104.95%, which was slightly lower than compound **11** (107.05%), which contains glucose. Thus, the apparent advantage observed for the galactoside over the glucoside at the FMG level should not be generalized to longer glycosides. Increased sugar-chain size, polarity, and other structural differences may become more influential in the diglycosides.

Overall, two preliminary SAR trends emerged among the flavonoids examined in this study. First, within compounds **1**–**3**, the absence of hydroxyl groups at C-3′ and C-3 was associated with stronger NO-inhibitory activity. Second, among the glycosides examined, shorter sugar chains tended to be associated with lower normalized NO levels.

At the monoglycoside level, isorhamnetin-3-O-galactoside (**8**) was substantially more active than the corresponding glucoside (**9**). Because the galactoside–glucoside comparison is based on a single matched FMG pair, this observation should be regarded as a preliminary SAR finding rather than a general rule for flavonoid glycosides.

### 2.4. Molecular Docking Analysis

To provide complementary structural information, compounds **1**–**11** were docked into the binding sites of iNOS (PDB: 3E7G) and COX-2 (PDB: 3LN1). Because docking predictions do not account for cellular permeability, metabolism, or other biological factors, the docking results are presented only as qualitative structural information and are not used to infer cellular potency or mechanism of action.

#### 2.4.1. Docking Validation

**Figure 4:**
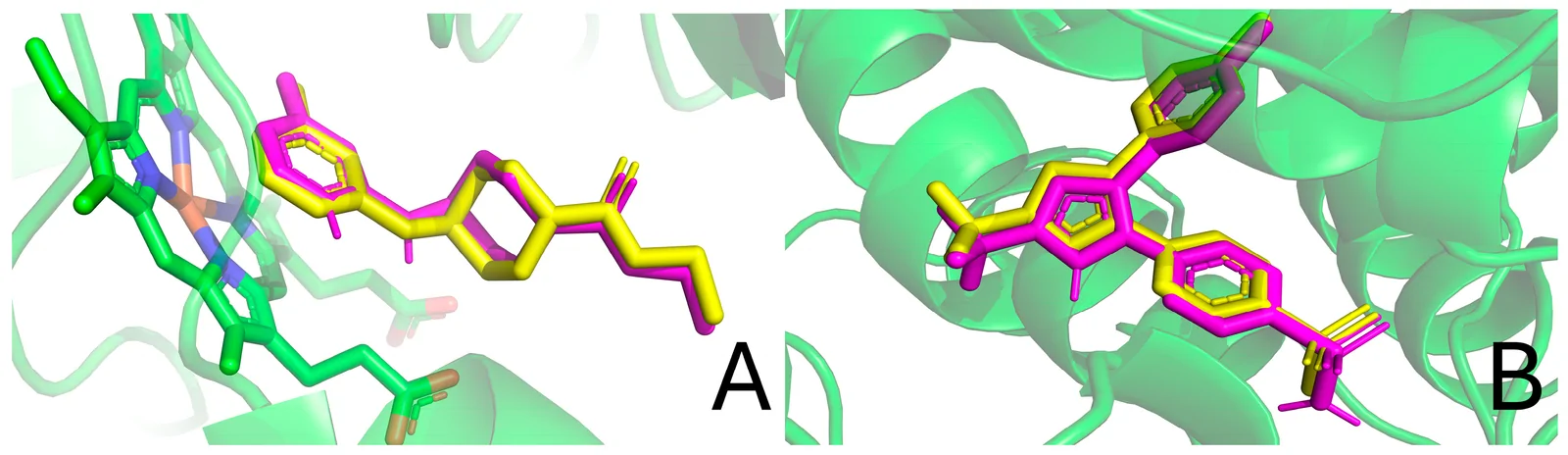
Redocking validation of co-crystallized ligands. (**A**) Superimposition of the crystallographic ligand ethyl 4-[(4-methylpyridin-2-yl)amino]piperidine-1-carboxylate (AT2, AR-C95791) (yellow) and the redocked pose (magenta) within the iNOS heme-binding pocket (PDB: 3E7G). The heme cofactor (HEM) is shown as sticks colored by element (green, carbon; blue, nitrogen; red, oxygen) (**B**) Superimposition of the crystallographic ligand celecoxib (yellow) and the redocked pose (magenta) within the COX-2 binding pocket (PDB: 3LN1).

The reliability of the docking protocol was assessed by re-docking the co-crystallized ligands into their respective binding sites. The root-mean-square deviation (RMSD) between the best re-docked pose and the crystallographic conformation was 0.490 Å for iNOS (19 heavy atoms) and 0.909 Å for COX-2 (26 heavy atoms) (Figure 4). Both values are below the commonly accepted threshold of 2.0 Å [24], supporting the suitability of the protocol for pose prediction.

#### 2.4.2. Binding Energy Analysis

Predicted binding energies of compounds **1**–**11** with iNOS and COX-2 are summarized in Table 3. The scores ranged from −7.78 to −9.69 kcal/mol for iNOS and from −7.43 to −10.33 kcal/mol for COX-2. Notably, these values did not parallel the cellular NO-inhibitory activities. Isorhamnetin-3-O-galactoside (**8**), which exhibited a low normalized NO level (22.22%) in cellular assays, displayed among the least favorable predicted binding energies for both targets (−7.78 kcal/mol for iNOS and −7.43 kcal/mol for COX-2). Conversely, rutin (**5**) and narcissoside (**11**), which showed weak or absent cellular activity (normalized NO levels 95.78% and 107.05%, respectively), displayed relatively favorable iNOS docking scores of −9.69 and −9.68 kcal/mol. Similarly, quercetin (**3**), which showed only moderate cellular activity (85.88%), had a more negative iNOS binding energy (−9.36 kcal/mol) than the more active apigenin (**1**, −8.78 kcal/mol) and kaempferol (**2**, −8.68 kcal/mol). These discrepancies indicate that the docking scores should not be used to predict cellular NO-inhibitory activity or to explain the observed SAR [25].

#### 2.4.3. Binding Mode Analysis

Given the lack of correlation between docking scores and cellular activity [25], the predicted poses were inspected qualitatively to assess whether structural modifications were accompanied by altered ligand orientations.

**Figure 5:**
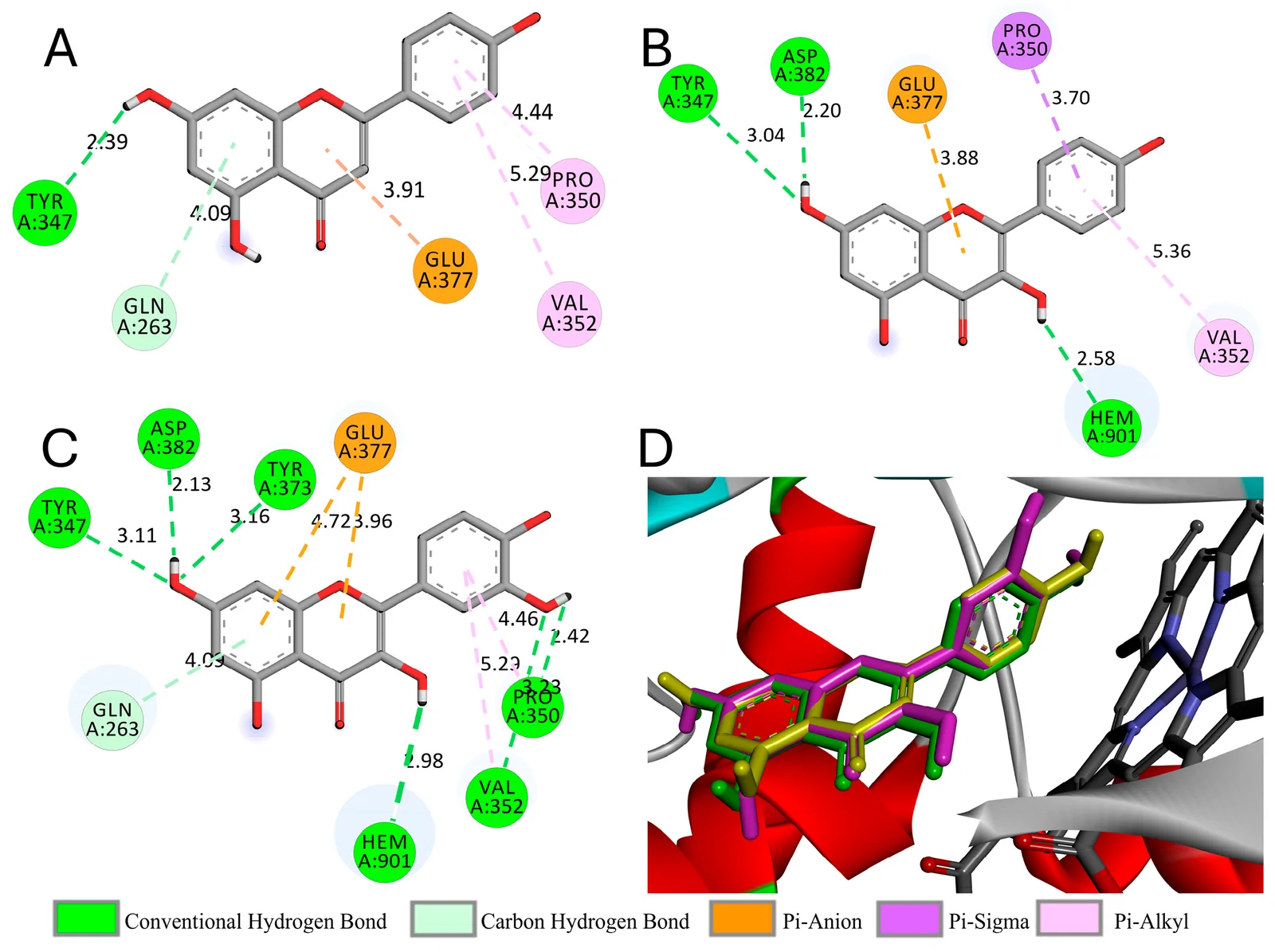
Two-dimensional (2D) molecular docking diagrams and an overlay of compounds **1**–**3** docked with iNOS. (**A**) 2D docking diagram of compound **1**; (**B**) 2D docking diagram of compound **2**; (**C**) 2D docking diagram of compound **3**; (**D**) overlay of compounds **1**–**3**, with flavonoid molecules colored yellow for compound **1**, green for compound **2**, and magenta for compound **3**.

**Figure 6:**
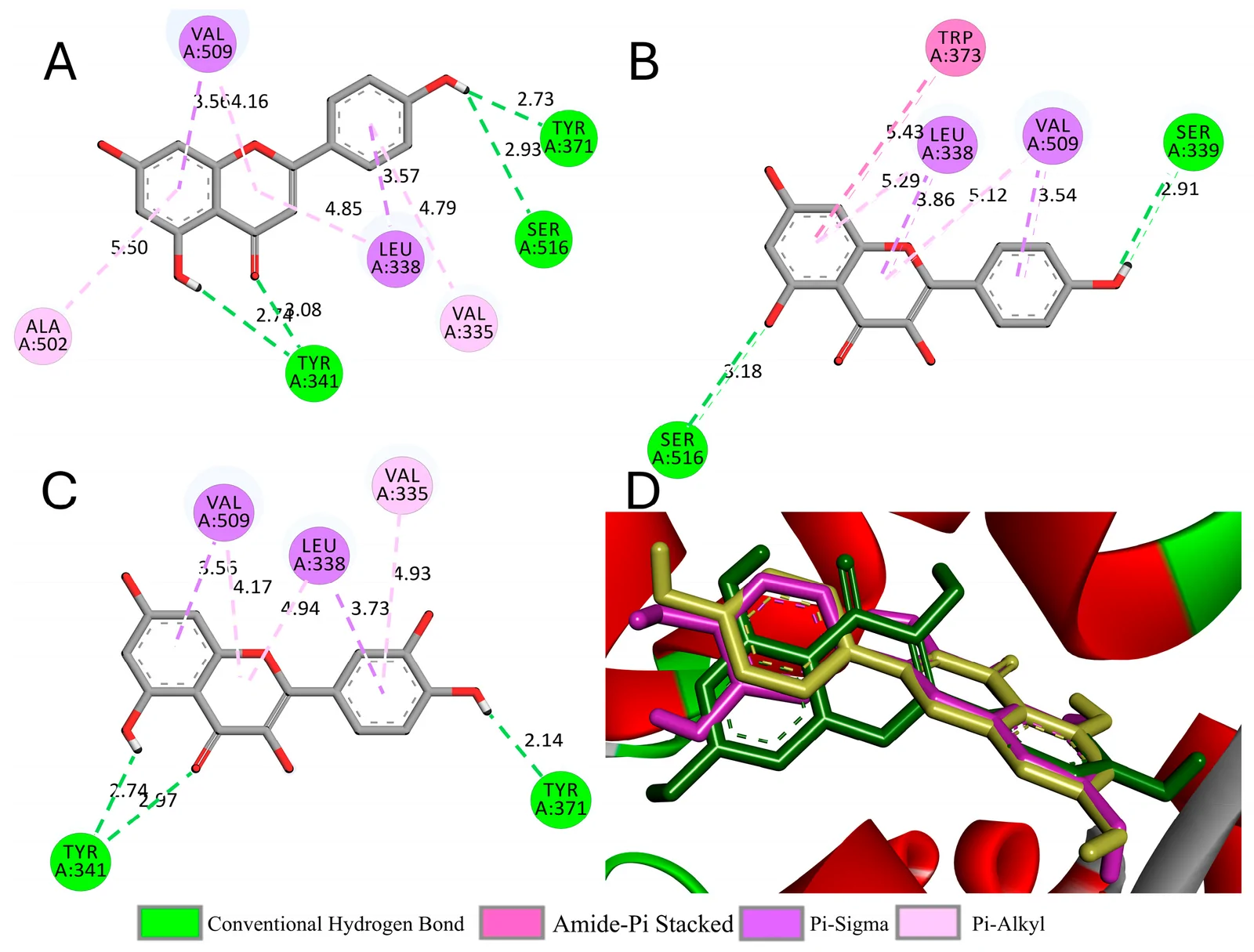
Two-dimensional (2D) molecular docking diagrams and an overlay of compounds **1**–**3** docked with COX-2. (**A**) 2D docking diagram of compound **1**; (**B**) 2D docking diagram of compound **2**; (**C**) 2D docking diagram of compound **3**; (**D**) overlay of compounds **1**–**3,** with flavonoid molecules colored yellow for compound **1**, green for compound **2**, and magenta for compound **3**.

Progressive differences in hydroxyl substitution among compounds **1**–**3** were accompanied by differences in their predicted orientations and contact patterns within both iNOS and COX-2 (Figure 5 and Figure 6). Notably, quercetin (**3**) displayed more favorable docking scores than apigenin (1) and kaempferol (**2**), despite showing weaker cellular NO-inhibitory activity (Table 3). This discrepancy further illustrates that the predicted docking interactions do not directly reflect cellular NO-inhibitory activity [25]. Therefore, these pose differences are presented only as qualitative structural observations and should not be interpreted as evidence of direct iNOS or COX-2 inhibition.

**Figure 7:**
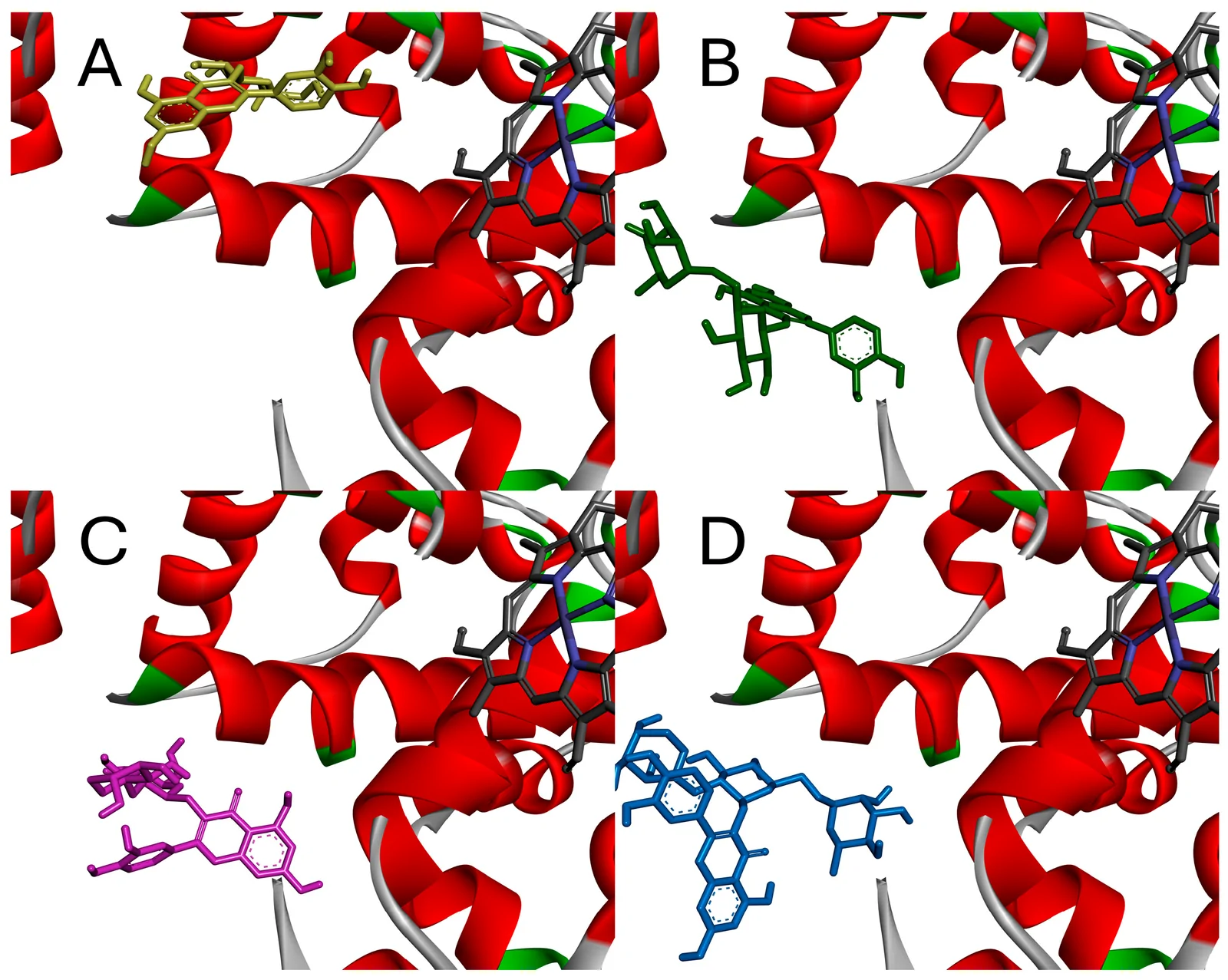
Overall views of compounds **4**–**7** docked with iNOS. (**A**) Overall view of compound **4**; (**B**) overall view of compound **5**; (**C**) overall view of compound **6**; (**D**) overall view of compound **7**.

**Figure 8:**
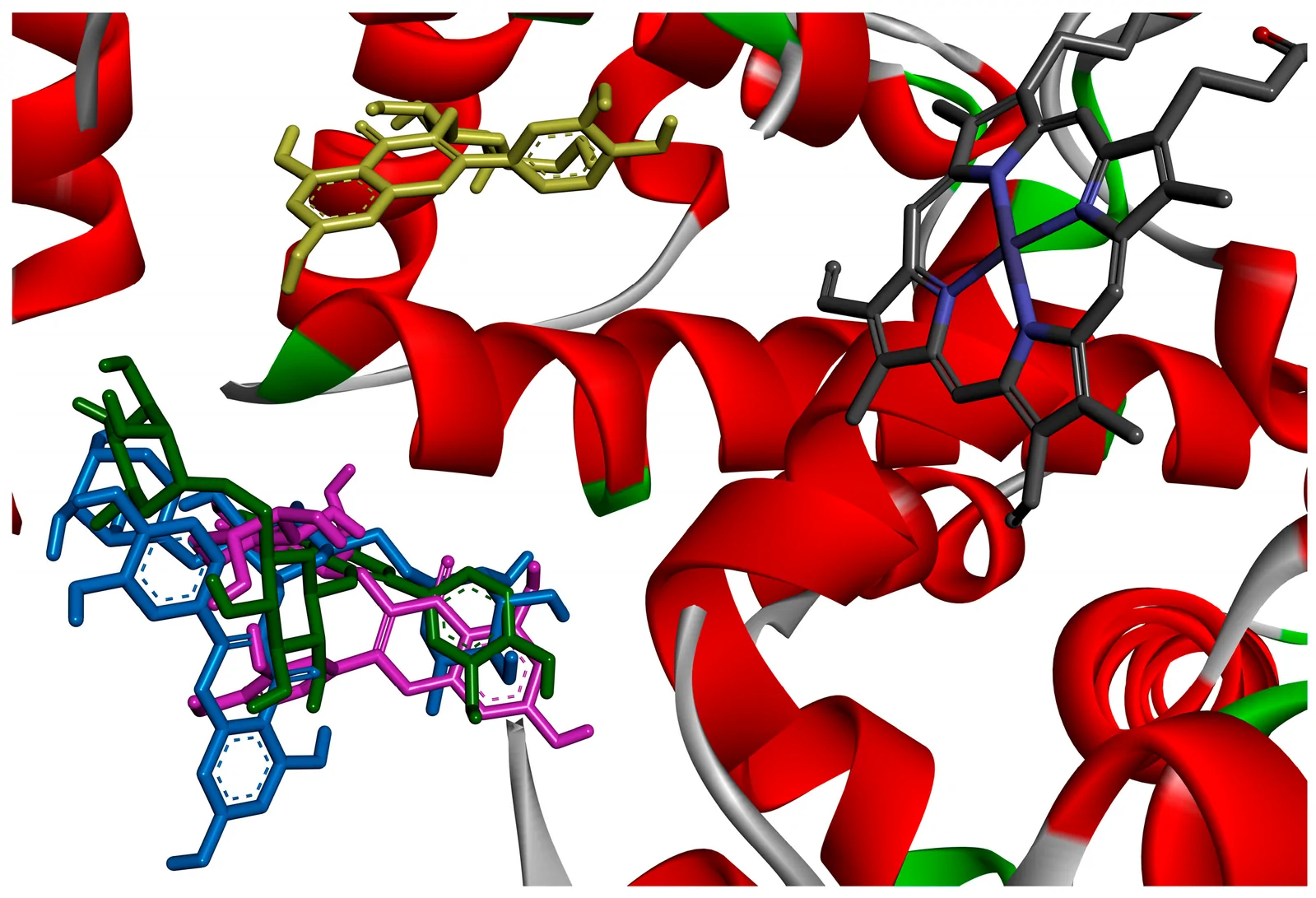
Overlay of compounds **4**–**7** docked with COX-2. Flavonoid molecules are colored yellow for compound **4**, green for compound **5**, pink for compound **6**, and blue for compound **7**.

In the glycosylated series, extension of the sugar chain markedly altered the predicted binding poses. In iNOS (Figure 7), the flavonoid core of the monoglycoside **4** occupied the inner active-site region [26], whereas the di- and triglycosides (**5**–**7**) shifted outward, with their elongated glycosyl chains projecting into the pocket and displacing the aglycone toward the entrance. In COX-2 (Figure 8), compound **4** nestled within the pocket interior, while **5**–**7** adopted more peripheral binding poses near the pocket entrance. This sugar-chain-driven shift from deep to peripheral binding was observed alongside the cellular activity differences among the glycosides. Nevertheless, because several weakly active glycosides still displayed favorable docking scores, these pose differences are presented as qualitative structural insight rather than a complete mechanistic account of the glycosylation effect. Detailed docking interaction parameters are provided in Supplementary Figures S45–S110.

**Figure 9:**
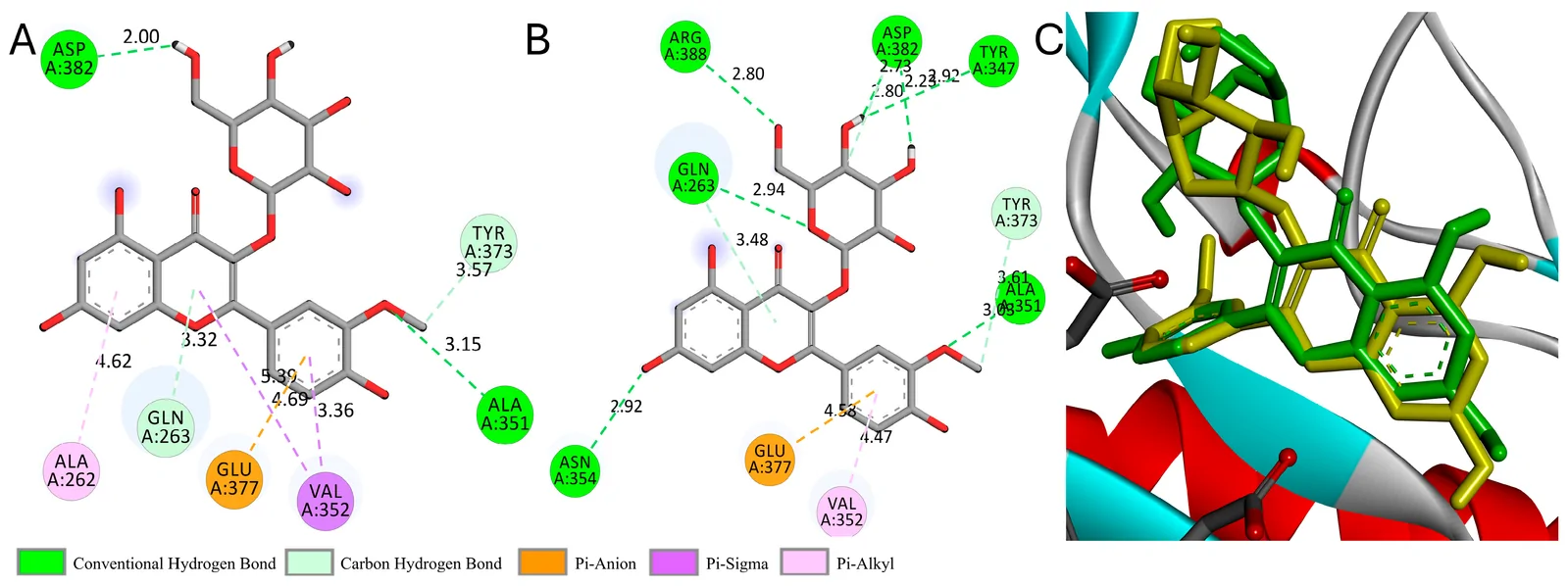
Two-dimensional (2D) molecular docking diagrams and an overlay of compounds **8** and **9** docked with iNOS. (**A**) 2D docking diagram of compound **8**; (**B**) 2D docking diagram of compound **9**; (**C**) overlay of compounds **8** and **9**, with flavonoid molecules colored yellow for compound **8** and green for compound **9**.

**Figure 10:**
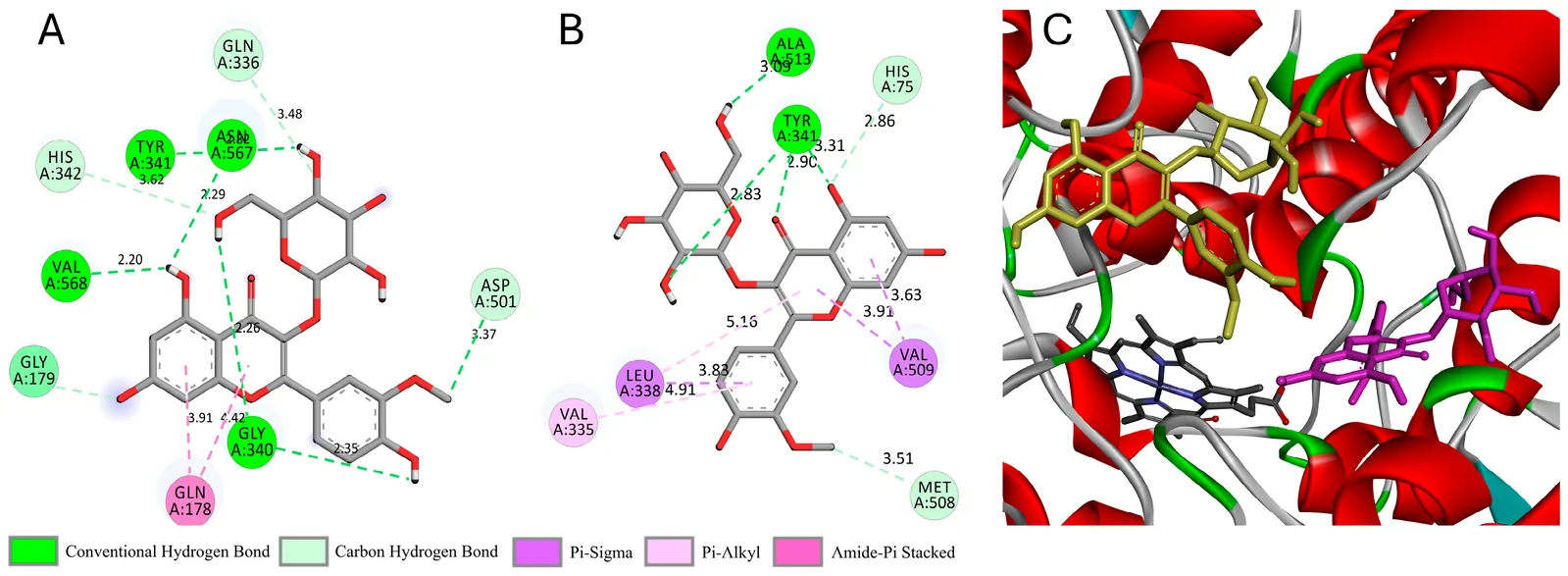
Two-dimensional (2D) molecular docking diagrams and an overlay of compounds **8** and **9** docked with COX-2. (**A**) 2D docking diagram of compound **8**; (**B**) 2D docking diagram of compound **9**; (**C**) overlay of compounds **8** and **9**, with flavonoid molecules colored yellow for compound **8** and pink for compound **9**.

At the monoglycoside level, isorhamnetin-3-O-galactoside (**8**) and isorhamnetin-3-O-glucoside (**9**) adopted distinct predicted orientations in both targets (Figure 9 and Figure 10). The configurational difference at C-4′′ of the sugar moiety appeared to modify the distribution of predicted contacts without producing a proportionally large change in overall docking score, despite the substantial difference in cellular NO-inhibitory activity between the two compounds. Nevertheless, because this comparison is based on a single matched pair and computational docking rather than direct enzyme-inhibition measurements, these docking results should be regarded as qualitative structural observations rather than evidence for a general galactoside-versus-glucoside SAR.

Overall, the docking results suggest that both hydroxylation pattern and glycosylation can alter predicted binding orientations. However, because docking scores did not correlate with cellular NO-inhibitory activity, and because predicted contacts are computational estimates rather than experimental observations, these results are intended solely as complementary structural information. They should not be interpreted as experimental validation of iNOS or COX-2 inhibition.

## 3. Materials and Methods

### 3.1. Plant Material

Fruits of *Pyrus communis* L. ‘Kashi’ were collected in October 2022 in Shufu County, Kashi Region, Xinjiang, China. The plant material was authenticated by Aishajiang Abudushalamu (College of Life and Geography Sciences, Kashi University) as *Pyrus communis* L. ‘Kashi’ (Rosaceae). A voucher specimen (No. KKA-202311001) was deposited at the Key Laboratory of Xinjiang Native Medicinal and Edible Plant Resources Chemistry, Kashi University.

### 3.2. Extraction and Isolation

Fresh fruits of *Pyrus communis* L. ‘Kashi’ (45 kg) were extracted by maceration with 70% ethanol (15 L for each extraction) at room temperature for 24 h, and the extraction was repeated ten times. The combined extracts were concentrated under reduced pressure to afford 5.2 kg of crude extract, which was suspended in water and sequentially partitioned with petroleum ether, dichloromethane (DCM), ethyl acetate (EtOAc), and n-butanol (n-BuOH).

The EtOAc fraction (60.0 g) was subjected to silica gel column chromatography (CC) eluting with a gradient of DCM/MeOH (100:1 → 0:100, *v*/*v*). Fractions were pooled based on TLC and High-Performance Liquid Chromatography (HPLC) analyses to afford 16 fractions (Fr.1–Fr.16). Fr.4 (6.6 g) was further separated by silica gel CC into eight subfractions; subfraction Fr.4.2 was purified by Sephadex LH-20 CC followed by semi-preparative HPLC (14% MeOH, isocratic) to yield compound **3** (4.5 mg, tR = 28.0 min). Fr.6 (8.1 g) was chromatographed over Sephadex LH-20 to give six subfractions. Subfractions Fr.6.3 and Fr.6.6 were further purified by Sephadex LH-20 (MeOH) and semi-preparative HPLC (10% MeOH, isocratic) to afford compounds **8** (9.1 mg, tR = 49.0 min) and **9** (6.4 mg, tR = 53.0 min), respectively.

The n-BuOH fraction (1235.6 g) was fractionated on a D101 macroporous resin column eluting with a stepwise gradient of MeOH/H_2_O (0–90% MeOH). The eluates were combined into six major fractions (Fr.1–Fr.6) according to TLC and HPLC monitoring. Fr.5 (25.8 g) was separated on a polyamide column into an aqueous fraction (Fr.5.1) and an 80% MeOH fraction (Fr.5.2). Fr.5.1 was subjected to silica gel CC using a gradient of DCM/MeOH/HCOOH (100:1:0.1 → 1:1:0.1, *v*/*v*/*v*). Subfraction Fr.5.1.3 was purified by semi-preparative HPLC (13% MeOH, isocratic) to give compounds **1** (5.0 mg, tR = 21.5 min) and **2** (4.3 mg, tR = 37.0 min). Fr.5.2 was further fractionated by silica gel CC into four subfractions (Fr.5.2.1–Fr.5.2.4). Fr.5.2.1 was chromatographed over Sephadex LH-20 (MeOH) and then purified by semi-preparative HPLC (15% MeOH, isocratic) to yield compound **10** (4.0 mg, tR = 48.0 min). Fr.5.2.2 was purified directly by semi-preparative HPLC (35% MeOH, isocratic) to afford compound **4** (3.4 mg, tR = 22.0 min). Fr.6 (15.3 g) was divided on a polyamide column into an aqueous fraction (Fr.6.1) and an 80% MeOH fraction (Fr.6.2). Fr.6.1 was chromatographed on a reversed-phase C18 column followed by semi-preparative HPLC (23% MeOH, isocratic) to give compounds **6** (3.9 mg, tR = 20.0 min) and **11** (3.2 mg, tR = 23.0 min). Fr.6.2 was first subjected to reversed-phase C18 chromatography, then to Sephadex LH-20 (MeOH), and finally purified by semi-preparative HPLC (17% MeOH, isocratic) to afford compounds **5** (3.2 mg, tR = 37.0 min) and **7** (3.7 mg, tR = 43.0 min) [27].

The isolated compounds were identified by NMR (Bruker Avance 600 MHz, Bruker, Karlsruhe, Germany; Varian Mercury Plus 400 MHz, Varian, Palo Alto, CA, USA). The HRESIMS data were determined on a quadrupole-orbitrap-HRMS (Thermo Fisher Scientific, Bremen, Germany) connected to an Ultimate 3000 Rapid Separation system (RSLC, Dionex, Sunnyvale, CA, USA), and HPLC (Shimadzu LC-20AT, Shimadzu, Kyoto, Japan) analyses and by comparison of their spectroscopic data with published data. All NMR spectra were recorded in CD_3_OD. Due to rapid proton exchange with the deuterated solvent, hydroxyl (OH) proton signals were not observed in the ^1^H NMR spectra; accordingly, hydroxyl positions were assigned based on ^13^C NMR chemical shifts and comparison with published spectroscopic data.

### 3.3. Cell Culture and Treatment

RAW264.7 mouse macrophages were obtained from Procell Life Science & Technology Co., Ltd. (Wuhan, China). The RAW264.7 cells were maintained in Dulbecco’s Modified Eagle Medium (DMEM; Gibco, Thermo Fisher Scientific, Waltham, MA, USA) supplemented with 10% fetal bovine serum (FBS; BI, Beit Haemek, Israel) and 1% penicillin-streptomycin (P/S; HyClone, Logan, UT, USA) at 37 °C in a humidified atmosphere containing 5% CO_2_. The culture medium was refreshed every two days, and cells were continuously passaged until they reached 70 to 80% confluence. Test compounds were dissolved in dimethyl sulfoxide (DMSO; Sigma-Aldrich, St. Louis, MO, USA) using sonication to prepare 50 mM stock solutions, which were subsequently diluted in complete medium immediately prior to use, ensuring a final DMSO concentration of 0.1% (*v*/*v*).

### 3.4. Cell Viability Assay

RAW264.7 cells were seeded at a density of 8 × 10^3^ cells per well in 96-well tissue culture plates. Following overnight incubation, the cells were allocated into four groups: a control group treated with 0.1% DMSO, an LPS model group, a group treated with varying concentrations of the compounds in conjunction with LPS, and a compound-only group treated with the corresponding compounds in the absence of LPS, with each concentration tested in triplicate wells. For the initial viability assessment, compounds **1**–**11** were evaluated at a concentration of 100 μM. Additionally, compounds **1**, **2**, and **8** were further assessed at concentrations of 6.25, 12.5, 25, 50, and 100 μM in order to conduct concentration–response nitric oxide (NO) assays. After a 2-h pretreatment period with the compounds, LPS was introduced to the +LPS test groups at a final concentration of 1 μg/mL, whereas the corresponding −LPS groups were maintained without LPS, followed by a 24-h incubation. Subsequently, the medium was removed, and the cells were treated with MTT (5 mg/mL; Sigma-Aldrich) for 4 h. Afterward, the medium was completely aspirated, 150 μL of DMSO was added to each well, and the plates were gently agitated for 10 min. Absorbance values were then measured at 490 nm using an Agilent BioTek Synergy H1 Multimode Reader (BioTek, Winooski, VT, USA).

Viability (%) = (ODtreated/ODcontrol) × 100%

The data are presented as the mean ± standard deviation (SD) derived from three independent experiments. Statistical analysis was conducted using one-way analysis of variance (ANOVA), followed by Dunnett’s multiple comparisons test, utilizing GraphPad Prism version 9.0.

### 3.5. Evaluation of NO-Inhibitory Activity

RAW264.7 cells were seeded in 6-well plates at a density of 2.5 × 10^5^ cells per well and allowed to adhere for 16 h. The experimental groups comprised a normal control group (NC), an LPS model group, a flavonoid + LPS group (100 μM), and flavonoid groups (100 μM without LPS stimulation). Cells were pre-treated with various flavonoids for 2 h, followed by the addition of LPS (Solarbio, Beijing, China) at a final concentration of 1 μg/mL for 16 h. Nitric oxide (NO) production was assessed by adding 50 μL of cell supernatant to an equal volume of Griess reagent (Beyotime Biotechnology, Shanghai, China; Cat. No. S0021S), incubating for 3–5 min in the dark. Absorbance was measured at 540 nm using an Agilent BioTek Synergy H1 Multimode Reader (BioTek, Winooski, VT, USA), and nitrite concentrations were determined from a sodium nitrite standard curve (0–100 μM). Normalized NO levels were calculated by dividing the nitrite concentration of each sample by that of the LPS-stimulated control group (set as 100%). Each experiment included three replicate wells and was conducted in three independent trials. Data are expressed as the mean ± standard deviation (SD) and were analyzed using one-way ANOVA followed by Dunnett’s multiple comparisons test against the LPS group, utilizing GraphPad Prism 9.0 [28].

Based on the screening results, compounds **1**, **2**, and **8**, together with the dexamethasone positive control, were further evaluated at 6.25, 12.5, 25, 50, and 100 μM using the same pretreatment and stimulation protocol. IC_50_ values were calculated by fitting the concentration–response data to a four-parameter logistic equation (GraphPad Prism 9.0), with the bottom and top of the curve constrained to 0% and 100% inhibition, respectively, because the normalized response is bounded by these values. The goodness of fit was evaluated by the coefficient of determination (R^2^), and the 95% confidence intervals (CI) and Hill slopes were reported. Data are presented as the mean ± SD of three independent experiments.

### 3.6. Molecular Docking

Protein preparation. The crystal structures of the human iNOS oxygenase domain (PDB ID: 3E7G) [26] and murine (mouse) COX-2 in complex with celecoxib (PDB ID: 3LN1) [29] were retrieved from the RCSB Protein Data Bank. Initial pre-processing was performed in PyMOL (Version 2.5.4): for both proteins, only chain A was retained, and all water molecules and heteroatoms were removed, with the exception of the heme cofactor (HEM) in the iNOS structure. The co-crystallized ligands—ethyl 4-[(4-methylpyridin-2-yl)amino]piperidine-1-carboxylate (AT2, AR-C95791) from 3E7G and celecoxib from 3LN1—were separately extracted and saved as reference structures for subsequent redocking validation. The processed protein files were then imported into AutoDock Tools (Version 1.5.7) for further preparation, including the removal of remaining water molecules, addition of polar hydrogen atoms, and assignment of partial charges.

Ligand preparation. Three-dimensional structures of compounds **1**–**11** were obtained from the PubChem database and energy-minimized using Chem3D (Version 23.1.3). All ligands were prepared in AutoDock Tools by merging non-polar hydrogens, assigning rotatable bonds, and adding Gasteiger charges.

Grid box definition. The docking grid for iNOS was centered at (x = 56.03, y = 20.374, z = 79.669) with dimensions of 24.0 × 24.0 × 24.0 Å, following a previously reported protocol [30]. For COX-2, the grid was defined to fully encompass the celecoxib-binding site, consistent with a previously reported AutoDock Vina docking setup for this structure [31]; the grid center was determined by calculating the geometric center of the celecoxib binding site in PyMOL using the cmd.extent command (min: [25.378, −25.290, −20.210]; max: [35.816, −19.828, −11.306]); the averaged coordinates (x = 30.597, y = −22.559, z = −15.758) were used as the grid center with dimensions of 30.0 × 30.0 × 30.0 Å.

Docking validation. Prior to screening the test compounds, the docking protocol was validated by re-docking the respective co-crystallized ligands (AT2 into iNOS and celecoxib into COX-2) into their original binding sites using the grid parameters described above, following a previously reported redocking protocol [24]. All other parameters were maintained at AutoDock Vina defaults.

Docking procedure. Semi-flexible docking of compounds **1**–**11** was performed with AutoDock Vina (Version 1.2.5) [32] using the validated grid parameters. For each compound, the pose with the lowest predicted binding energy located within the predefined active-site region was selected for interaction analysis. Predicted binding energies (kcal/mol) were recorded for comparative purposes. Docking poses were visualized using Discovery Studio Visualizer (Version 4.5) and PyMOL.

### 3.7. cLogP and TPSA Calculation

The cLogP and TPSA values of the compounds were calculated using ChemDraw 20.0.

## 4. Conclusions

This study provides the first systematic isolation and structural characterization of flavonoids from *Pyrus communis* L. ‘Kashi’. Eleven flavonoids were identified, including aglycones, FMGs, FDGs, and FTGs. To the best of our knowledge, compound **6** was isolated from the genus *Pyrus* for the first time. Although compounds **7**, **8**, and **10** had previously been detected in pear skins by LC-DAD-ESI/MS profiling, the present study provides their first isolation and NMR-based structural identification from the genus *Pyrus*.

Evaluation of NO production in LPS-stimulated RAW264.7 macrophages showed that apigenin, kaempferol, and isorhamnetin-3-O-galactoside exhibited the strongest inhibitory activities among the tested compounds. Comparative SAR analysis revealed two main trends within the investigated compound set. Among compounds **1**–**3**, the absence of hydroxyl groups at C-3′ of the B ring and C-3 of the C ring was associated with stronger NO-inhibitory activity. Among the glycosides examined, shorter sugar chains tended to be associated with lower normalized NO levels. At the monoglycoside level, isorhamnetin-3-O-galactoside was substantially more active than the corresponding glucoside; however, this observation was based on a single matched pair and should not be generalized to other flavonoid glycosides.

Molecular docking indicated that changes in hydroxylation and glycosylation were accompanied by differences in predicted ligand orientation and residue-interaction patterns with iNOS and COX-2. However, docking-score rankings did not parallel the cellular NO-inhibitory activities of the tested compounds. Thus, the docking results provide complementary qualitative structural information rather than direct evidence of iNOS or COX-2 inhibition or experimental validation of an anti-inflammatory mechanism.

Overall, these findings expand the phytochemical characterization of *P. communis* L. ‘Kashi’ and provide comparative information on the NO-inhibitory activities and preliminary SAR of its flavonoid constituents. Further experimental studies are required to establish the molecular targets and mechanisms underlying these cellular effects.

## Figures and Tables

**Table 1:** Concentration–response parameters of compounds **1**, **2**, and **8**.

Compound	IC_50_ (µM)	95% CI of IC_50_	R^2^	nH
Apigenin (**1**)	22.75 ± 3.23	14.74–30.76	0.983 ± 0.010	1.74 ± 0.34
Kaempferol (**2**)	37.54 ± 4.40	26.60–48.48	0.975 ± 0.029	2.13 ± 0.91
Isorhamnetin-3-O-galactoside (**8**)	58.19 ± 10.41	32.33–84.05	0.972 ± 0.024	1.57 ± 0.31

**Table 2:** Physicochemical properties and normalized NO levels of flavonoids isolated from *Pyrus communis* ‘Kashi’ (*n* = 3).

Sample	Aglycone	Sugar Moiety	No. of Sugars	cLogP	TPSA	Normalized NO Levels
NC	–	–	–	–	–	5.78 ± 0.31
LPS	–	–	–	–	–	100
**1**	Apigenin	–	0	2.91	86.99	10.09 ± 0.64
**2**	Kaempferol	–	0	2.1	107.22	12.92 ± 0.87
**3**	Quercetin	–	0	1.5	127.45	85.88 ± 6.96
**4**	Quercetin	Glc	1	−0.34	206.6	94.01 ± 5.86
**5**	Quercetin	Glc-Rha	2	−1.36	265.52	95.78 ± 9.87
**6**	Quercetin	Glc-Ara	2	−1.83	265.52	101.76 ± 9.38
**7**	Quercetin	Glc-Rha-Xyl	3	−2.86	324.44	107.06 ± 2.22
**8**	Isorhamnetin	Gal	1	0.85	175.37	22.22 ± 1.36
**9**	Isorhamnetin	Glc	1	0.85	175.37	92.06 ± 7.63
**10**	Isorhamnetin	Gal-Rha	2	−0.92	254.52	104.95 ± 6.16
**11**	Isorhamnetin	Glc-Rha	2	−0.92	254.52	107.05 ± 7.88

Abbreviations: –, not applicable. TPSA, topological polar surface area; cLogP, calculated octanol–water partition coefficient. Glc, glucose; Rha, rhamnose; Ara, arabinose; Xyl, xylose; Gal, galactose. NC, normal control: unstimulated RAW264.7 cells treated with vehicle (DMSO) alone. LPS, lipopolysaccharide-stimulated control: cells treated with 1 μg/mL LPS alone; its NO level was set as 100%. Normalized NO levels are expressed as percentages of the LPS-stimulated control. Values below 100% indicate lower nitrite levels than the LPS control, whereas values above 100% indicate higher nitrite levels. Data are presented as mean ± SD from three independent biological experiments (n = 3).

**Table 3:** Molecular docking binding energies (kcal/mol) of compounds **1**–**11** with iNOS and COX-2.

Compound No.	Compound Name	iNOS	COX-2
**1**	Apigenin	−8.78	−9.98
**2**	Kaempferol	−8.68	−9.29
**3**	Quercetin	−9.36	−9.78
**4**	Isoquercitrin	−9.01	−9.22
**5**	Rutin	−9.69	−9.00
**6**	Quercetin-3-O-*L*-arabinopyranosyl-(1 → 2)-*D*-glucopyranoside	−8.78	−7.91
**7**	Quercetin-3-O-[2-O-*β*-xylopyranosyl-6-O-*α*-rhamnopyranosyl]-*β*-glucopyranoside	−9.48	−8.57
**8**	Isorhamnetin-3-O-galactoside	−7.78	−7.43
**9**	Isorhamnetin-3-O-glucoside	−8.85	−10.33
**10**	Isorhamnetin-3-O-robinobioside	−8.64	−8.63
**11**	Narcissoside	−9.68	−9.12

## Data Availability

The original contributions presented in the study are included in the article/Supplementary Materials. Further inquiries can be directed to the corresponding authors.

## Supplementary Materials

The following supporting information can be downloaded at: https://www.mdpi.com/article/10.3390/molecules31213859/s1, Text S1: Full ^1^H NMR, ^13^C NMR and HRMS (ESI) spectroscopic data of compounds **1**–**11**. Figures S1–S44: ^1^H NMR, ^13^C NMR, HRMS (ESI), and HPLC data of compounds **1**–**11**. Figures S45–S77: Overall views, enlarged views and 2D interaction diagrams of compounds **1**–**11** docked with iNOS (3 figures for each compound). Figures S78–S110: Overall views, enlarged views and 2D interaction diagrams of compounds **1**–**11** docked with COX-2 (3 figures for each compound). Figure S111: MTT Cell Viability Assay of Compounds **1**, **2** and **8** at Different Concentrations. The Supplementary Materials associated with this manuscript are available at https://doi.org/10.5281/zenodo.21903027.
