# Supplementary material for: Flavonoids from Pyrus communis L. ‘Kashi’: Isolation, Structural Identification, NO Inhibitory Activity, and Molecular Docking
Source: Molecules. 2026 Oct 26;31(21):3859. doi: 10.3390/molecules31213859 (PMC13765364; doi:10.3390/molecules31213859)
Supplement: Supplementary file 1 [file molecules-31-03859-s001.zip › molecules-4529266-supplementary.pdf]

Supplementary Materials:

Flavonoids from *Pyrus communis* L. 'Kashi': Isolation, Structural Identification, NO Inhibitory Activity, and Molecular Docking

Zhuang-Zhuang Tang †, Erkin Tursun†, Adilijiang Abulimiti, Yu-Jie Qiao, Yu Xue, Xue-Lin Cheng, Maimaiti Tuerxun *, Abdulla Yusuf * and Adila Abudurexiti *

Key Laboratory of Xinjiang Native Medicinal and Edible Plant Resources Chemistry, College of Chemistry and Environmental Science, Kashi University, Kashi 844000, China; 13175099823@163.com (Z.-Z.T.); erkin0998@163.com (E.T.); adilijiang2022@163.com (A.A.); 13772786008@163.com (Y.-J.Q.); xy2187231628@163.com (Y.X.); 15022831884@163.com (X.-L.C.)

* Correspondence: 15722632656@163.com (M.T.); kashidaxue_abudula@163.com (A.Y.); aji_228@163.com (A.A.)

† These authors contributed equally to this work.

Text S1: Full ^{1}H NMR, ^{13}C NMR and HRMS (ESI) spectroscopic data of compounds 1-11.

Figures S1–S44: ^{1}H NMR, ^{13}C NMR, HRMS (ESI), and HPLC data of compounds 1-11.

Figures S45-S77: Overall views, enlarged views and 2D interaction diagrams of compounds 1-11 docked with iNOS (3 figures for each compound).

Figures S78-S110: Overall views, enlarged views and 2D interaction diagrams of compounds 1-11 docked with COX-2 (3 figures for each compound).

Figure S111: Cell viability after treatment with compounds 1, 2, and 8 at different concentrations.

Text S1. Full ^{1}H NMR, ^{13}C NMR and HRMS (ESI) spectroscopic data of compounds 1–11

Compound 1 (Apigenin): yellow solid, HRMS (ESI) m/z calcd for $C_{15}H_{11}O_5$ $[M+H]^+$ 271.0601, found 271.0599; error − 0.7 ppm. Molecular formula: $C_{15}H_{10}O_5$. ^{1}H-NMR (CD_3OD, 600 MHz) *δ*: 7.85 (2H, d, *J* = 8.5 Hz, H-2′,6′), 6.93 (2H, d, *J* = 8.6 Hz, H-3′,5′), 6.59 (1H, s, H-3), 6.45 (1H, d, *J* = 2.1 Hz, H-8), 6.20 (1H, d, *J* = 2.1 Hz, H-6).^{13}C-NMR (CD_3OD, 151 MHz) *δ*: 183.87(C-4), 166.26(C-2,7), 163.23(C-9), 162.80(C-4′), 159.50(C-5), 129.44(C-2′,6′), 123.30(C-1′), 117.05(C-3′,5′), 105.20(C-10), 103.83(C-3), 100.30(C-6), 95.16(C-8).

Compound 2 (Kaempferol): yellow amorphous powder. HRMS (ESI) m/z calcd for $C_{15}H_{11}O_6$ $[M+H]^+$ 287.0550, found 287.0546; error − 1.4 ppm. Molecular formula: $C_{15}H_{10}O_6$. ^{1}H-NMR (CD_3OD, 600 MHz) *δ*: 8.09-8.07 (2H, m, H-2′,6′), 6.91-6.89 (2H, m, H-3′,5′), 6.38 (1H, d, *J* = 2.1 Hz, H-8), 6.17 (1H, d, *J* = 2.0 Hz, H-6). ^{13}C-NMR (CD_3OD, 151 MHz) *δ*: 177.34(C-4), 165.60(C-5), 162.50(C-7), 160.54(C-4′), 158.24(C-9), 148.01(C-2), 137.12(C-3), 130.67(C-2′,6′), 123.73(C-1′), 116.29(C-3′,5′), 104.52(C-10), 99.26(C-6), 94.46(C-8).

Compound 3 (Quercetin): yellow solid, HRMS (ESI) m/z calcd for $C_{15}H_{11}O_7$ $[M+H]^+$ 303.0500, found 303.0495; error –1.6 ppm. Molecular formula: $C_{15}H_{10}O_7$. ^{1}H-NMR (CD_3OD, 400 MHz) *δ*: 7.74 (1H, d, J = 2.2 Hz, H-2′), 7.63 (1H, dd, J = 8.5, 2.2 Hz, H-6′), 6.88 (1H, d, J = 8.5 Hz, H-5′), 6.39 (1H, d, J = 2.1 Hz, H-8), 6.18 (1H, d, J = 2.1 Hz, H-6). ^{13}C-NMR (CD_3OD, 101 MHz) *δ*: 177.34 (C-4), 165.58 (C-7), 162.52 (C-5), 158.23 (C-9), 148.77 (C-2), 148.00 (C-4′), 146.23 (C-3′), 137.24 (C-3), 124.15 (C-1′), 121.67 (C-6′), 116.22 (C-2′), 115.99 (C-5′),

104.52 (C-10), 99.23 (C-6), 94.40 (C-8).

Compound 4 (Isoquercitrin): yellow solid, HRMS (ESI) m/z calcd for $C_{21}H_{21}O_{12}$ $[M+H]^+$ 465.1028, found 465.1021; error − 1.5 ppm. Molecular formula: $C_{21}H_{20}O_{12}$. ^{1}H-NMR (CD_3OD, 600 MHz) δ: 7.71 (1H, d, J = 2.2 Hz, H-2′), 7.59 (1H, dd, J = 8.4, 2.2 Hz, H-6′), 6.87 (1H, d, J = 8.4 Hz, H-5′), 6.40 (1H, d, J = 1.9 Hz, H-8), 6.21 (1H, d, J = 2.0 Hz, H-6), 5.25 (1H, d, J = 7.7 Hz, H-1″), 3.71 (1H, dd, J = 11.9, 2.4 Hz, H-6″a), 3.58 (1H, dd, J = 11.9, 5.4 Hz, H-6″b), 3.48 (1H, dd, J = 9.2, 7.7 Hz, H-2″), 3.43 (1H, t, J = 9.0 Hz, H-3″), 3.35 (1H, t, J = 9.3 Hz, H-4″), 3.24-3.21 (1H, m, H-5″). ^{13}C-NMR (CD_3OD, 151MHz) δ: 179.51(C-4), 166.06(C-7), 163.08(C-5), 159.02(C-9), 158.49(C-2), 149.87(C-3′), 145.93(C-4′), 135.61(C-3), 123.19(C-1′), 123.07(C-6′), 117.53(C-5′), 116.00(C-2′), 105.69(C-10), 104.28(C-1″), 99.89(C-6), 94.70(C-8), 78.41(C-5″), 78.12(C-3″), 75.72(C-2″), 71.22(C-4″), 62.54(C-6″).

Compound 5 (Rutin): yellow solid, HRMS (ESI) m/z calcd for $C_{27}H_{31}O_{16}$ $[M+H]^+$ 611.1607, found 611.1601; error −1.0 ppm. Molecular formula: $C_{27}H_{30}O_{16}$. ^{1}H-NMR (CD_3OD, 400 MHz)δ: 7.67 (1H, d, J = 2.2 Hz, H-2′), 7.63 (1H, dd, J = 8.5, 2.2 Hz, H-6′), 6.87 (1H, d, J = 8.5 Hz, H-5′), 6.39 (1H, d, J = 2.1 Hz, H-8), 6.20 (1H, d, J = 2.1 Hz, H-6), 5.10 (1H, d, J = 7.5 Hz, H-1″), 4.52 (1H, d, J = 1.8 Hz, H-1‴), 3.81 (1H, d, J = 10.3 Hz, H-6″a), 3.65 (1H, dd, J = 3.4, 1.6 Hz, H-2‴), 3.55 (1H, dd, J = 9.5, 3.4 Hz, H-3‴), 3.51-3.43 (2H, m, H-2″, H-5‴), 3.41-3.33 (3H, m, H-3″, H-6″b, H-5″), 3.27 (2H, m, H-4″, H-4‴), 1.12 (3H, d, J = 6.2 Hz, H-6‴). ^{13}C-NMR (CD_3OD, 101MHz) δ: 179.35(C-4), 166.04(C-7), 162.93(C-5), 159.31(C-9), 158.46(C-2), 149.80(C-4′), 145.80(C-3′), 135.62(C-3), 123.56(C-6′), 123.07(C-1′), 117.68(C-3′), 116.04(C-5′), 105.59(C-10), 104.75(C-1″), 102.39(C-1‴), 99.97(C-6), 94.86(C-8), 78.15(C-3″), 77.17(C-5″), 75.69(C-2″), 73.91(C-4‴), 72.22(C-3‴), 72.07(C-2‴), 71.36(C-4″), 69.68(C-5‴), 68.54(C-6″), 17.87(C-6‴).

Compound 6(quercetin 3-O-*L*-arabinopyranosyl-(1→2)-*D*-glucopyranoside): yellow solid, HRMS (ESI) m/z calcd for $C_{26}H_{28}O_{16}Na$ $[M+Na]^+$ 619.1274, found 619.1259; error − 2.4 ppm. Molecular formula: $C_{26}H_{28}O_{16}$. ^{1}H-NMR (CD_3OD, 600 MHz) δ: 7.64-7.63 (2H, m, H-2′, H-6′), 6.88-6.86 (1H, m, H-5′), 6.38 (1H, d, J = 2.1 Hz, H-8), 6.19 (1H, d, J = 2.1 Hz, H-6), 5.50 (1H, d, J = 7.6 Hz, H-1″), 4.76-4.75 (1H, m, H-1‴), 3.93 (1H, dd, J = 11.7, 5.0 Hz, H-5‴a), 3.73-3.70 (2H, m, H-2″, H-6″a), 3.61-3.56 (2H, m, H-3″, H-6″b), 3.51-3.49 (1H, m, H-4‴), 3.40-3.36 (3H, m, H-4″, H-2‴, H-3‴), 3.25-3.21 (2H, m, H-5″, H-5‴b). ^{13}C-NMR (CD_3OD, 151MHz) δ: 179.59(C-4), 165.97(C-7), 163.17(C-5), 158.43(C-2), 158.26(C-9), 149.74(C-4′), 146.07(C-3′), 135.14(C-3), 123.40(C-2′), 123.22(C-1′), 117.31(C-6′), 116.05(C-5′), 105.77(C-10), 105.38(C-1‴), 100.86(C-1″), 99.79(C-6), 94.58(C-8), 82.33(C-2″), 78.38(C-3″), 78.24(C-5″), 77.05(C-3‴), 74.91(C-2‴), 71.06(C-4″), 71.00(C-4‴), 66.65(C-5‴), 62.41(C-6″).

Compound 7(Quercetin-3-O-[2-O-*β*-xylopyranosyl-6-O-*α*-Rhamnopyranosyl]-*β*-glucopyranoside): yellow solid, HRMS (ESI) m/z calcd for $C_{32}H_{39}O_{20}$ $[M+H]^+$ 743.2017, found 743.2018; error +0.1 ppm. Molecular formula: $C_{32}H_{38}O_{20}$. ^{1}H-NMR (CD_3OD, 600 MHz) δ: 7.63 -7.62 (2H, m, H-2′, 6′), 6.88 (1H, d, J = 9.0 Hz, H-5′), 6.38 (1H, d, J = 2.1 Hz, H-8), 6.20 (1H, d, J = 2.0 Hz, H-6), 5.41 (1H, d, J = 7.6 Hz, H-1″), 4.79-4.78 (1H, m, H-1⁗), 4.51 (1H, d, J = 1.7 Hz, H-1‴), 3.97 (1H, dd, J = 11.6, 4.9 Hz, H-5⁗a), 3.81 (1H, dd, J = 11.1, 1.5 Hz, H-6″a), 3.71 (1H, dd, J = 9.0, 7.6 Hz, H-2″), 3.60-3.57 (2H, m, H-2‴, 3″), 3.52-3.49 (2H, m, H-4⁗, 3‴), 3.45-3.42 (1H, m, H-5‴), 3.45-3.37 (4H, m, H-2⁗, 3⁗, H- 5″,6″b), 3.35-3.33 (1H, m, H-4″), 3.28-3.24 (2H, m, H-4‴, H-5⁗b), 1.09 (3H, d, J = 6.2 Hz, H-6‴). ^{13}C-NMR (CD_3OD, 151MHz) δ: 179.46(C-4), 165.80(C-7), 163.08(C-5), 158.66(C-9), 158.45(C-2),

149.67(C-4′), 145.95(C-3′), 134.96(C-3), 123.54(C-6′), 123.30(C-1′), 117.52(C-2′), 116.07(C-5′), 105.75(C-10), 105.16(C-1′′′′), 102.15(C-1′′′), 100.90(C-1′′), 99.83(C-6), 94.75(C-8), 81.98(C-2′′), 78.23(C-3′′), 77.10(C-5′′), 76.84(C-3′′′′), 74.70(C-2′′′′), 73.90(C-4′′′), 72.24(C-3′′′), 72.11(C-2′′′), 71.38(C-4′′), 70.98(C-4′′′′), 69.71(C-6′′), 68.10(C-5′′′), 66.53(C-5′′′′), 17.84(C-6′′′).

Compound 8 (Isorhamnetin-3-O-galactoside): yellow solid, HRMS (ESI) m/z calcd for $C_{22}H_{22}O_{12}Na$ $[M+Na]^+$ 501.1009, found 501.0989; error − 4.0 ppm. Molecular formula: $C_{22}H_{22}O_{12}$. ^{1}H-NMR (CD_3OD, 600 MHz) δ: 8.04 (1H, d, J = 2.1 Hz, H-2′), 7.58 (1H, dd, J = 8.4, 2.1 Hz, H-6′), 6.90 (1H, d, J = 8.4 Hz, H-5′), 6.41 (1H, d, J = 2.1 Hz, H-8), 6.21 (1H, d, J = 2.1 Hz, H-6), 5.31 (1H, d, J = 7.8 Hz, H-1′′), 3.96 (3H, s, $-OCH_3$). ^{13}C-NMR (CD_3OD, 151 MHz) δ: 179.54 (C-4), 166.07 (C-7), 163.08 (C-5), 158.67 (C-2), 158.48 (C-9), 150.86 (C-3′), 148.44 (C-4′), 135.44 (C-3), 123.59 (C-6′), 123.01 (C-1′), 115.94 (C-5′), 114.55 (C-2′), 105.74 (C-10), 104.43 (C-1′′), 99.90 (C-6), 94.73 (C-8), 77.27 (C-5′′), 75.00 (C-3′′), 73.15 (C-2′′), 70.01 (C-4′′), 62.18 (C-6′′), 56.94 ($-OCH_3$).

Compound 9 (Isorhamnetin-3-O-glucoside): yellow solid, HRMS (ESI) m/z calcd for $C_{22}H_{23}O_{12}$ $[M+H]^+$ 479.1184, found 479.1171; error − 2.7 ppm. Molecular formula: $C_{22}H_{22}O_{12}$. ^{1}H-NMR (CD_3OD, 600 MHz) δ: 7.92 (1H, d, J = 2.1 Hz, H-2′), 7.57 (1H, dd, J = 8.4, 2.1 Hz, H-6′), 6.89 (1H, d, J = 8.4 Hz, H-5′), 6.36 (1H, d, J = 2.1 Hz, H-8), 6.18 (1H, d, J = 2.1 Hz, H-6), 5.40-5.39 (1H, m, H-1′′), 3.94 (3H, s, $-OCH_3$), 3.74 (1H, dd, J = 11.9, 2.4 Hz, H-6′′b), 3.56 (1H, dd, J = 11.9, 5.5 Hz, H-6′′a), 3.49-3.44 (2H, m, H-2′′, H-4′′), 3.35-3.32 (1H, m, H-3′′), 3.27-3.24 (1H, m, H-5′′). ^{13}C-NMR (CD_3OD, 151 MHz) δ: 179.36 (C-4), 166.01 (C-7), 163.07 (C-5), 158.59 (C-2), 158.42 (C-9), 150.82 (C-3′), 148.35 (C-4′), 135.32 (C-3), 123.80 (C-6′), 123.04 (C-1′), 115.96 (C-5′), 114.34 (C-2′), 105.75 (C-10), 103.67 (C-1′′), 99.90 (C-6), 94.72 (C-8), 78.51 (C-5′′), 78.05 (C-3′′), 75.91 (C-2′′), 71.47 (C-4′′), 62.54 (C-6′′), 56.75 ($-OCH_3$)

Compound 10 (Isorhamnetin-3-O-robinobioside): yellow solid, HRMS (ESI) m/z calcd for $C_{28}H_{32}O_{16}Na$ $[M+Na]^+$ 647.1586, found 647.1574; error –1.9 ppm. Molecular formula: $C_{28}H_{32}O_{16}$. ^{1}H-NMR (CD_3OD, 400 MHz) δ: 8.03 (1H, d, J = 2.0 Hz, H-2′), 7.60 (1H, dd, J = 8.5, 2.0 Hz, H-6′), 6.91 (1H, d, J = 8.4 Hz, H-5′), 6.41 (1H, d, J = 2.0 Hz, H-8), 6.21 (1H, d, J = 1.9 Hz, H-6), 5.23 (1H, d, J =7.8 Hz, H-1′′), 4.53 (1H, d, J = 1.7 Hz, H-1′′′), 3.97(3H, s, $-OCH_3$), 3.84-3.72 (3H, m, H-2′′, 4′′, 6′′a), 3.66 (1H, t, J = 6.2 Hz, H-5′′), 3.59-3.44 (5H, m, H-3′′, 6′′b, H-2′′′, 3′′′, 5′′′), 3.26 (1H, d, J = 9.5 Hz, H-4′′′), 1.17 (3H, d, J = 6.2 Hz, H-6′′′). ^{13}C-NMR (CD_3OD, 101MHz) δ: 179.49(C-4), 166.18(C-7), 163.04(C-5), 158.88(C-9), 158.52(C-2), 150.89(C-4′), 148.41(C-3′), 135.48(C-3), 123.76(C-6′), 122.97(C-1′), 115.98(C-5′), 114.62(C-2′), 105.66(C-10), 104.92(C-1′′), 101.93(C-1′′′), 99.98(C-6), 94.87(C-8), 75.53(C-5′′), 75.01(C-3′′), 73.84(C-4′′′), 73.09(C-2′′), 72.29(C-3′′′), 72.08(C-2′′′), 70.03(C-4′′), 69.72(C-5′′′), 67.42(C-6′′), 56.95(-OCH3), 17.97(C-6′′′).

Compound 11 (Narcissoside): yellow solid, HRMS (ESI) m/z calcd for $C_{28}H_{33}O_{16}$ $[M+H]^+$ 625.1761, found 625.1759; error − 0.3 ppm. Molecular formula: $C_{28}H_{32}O_{16}$. ^{1}H-NMR (CD_3OD, 400 MHz) δ: 7.94 (1H, s, H-2′), 7.61 (1H, d, J = 8.0 Hz, H-6′), 6.91 (1H, d, J = 8.2 Hz, H-5′), 6.39 (1H, s, H-8), 6.20 (1H, s, H-6), 5.22 (1H, d, J = 6.5 Hz, H-1′′), 4.53 (1H, s, H-1′′′), 3.94 (3H, s, 3′-OCH_3), 3.82 (1H, d, J = 10.6 Hz, H-6′′a), 3.62 (1H, s, H-2′′′), 3.50 -3.35 (6H, m, H-3′′′, H-5′′′, H-2′′, H-3′′, ,H-5′′, H-6′′b), 3.26 (2H, t, J = 9.4 Hz, H-4′′, H-4′′′), 1.10 (3H, d, J = 6.2 Hz, H-6′′′). ^{13}C-NMR (CD_3OD, 101MHz) δ: 179.27(C-4), 166.05(C-7), 162.94(C-9), 158.82(C-5), 158.44(C-2), 150.82(C-4′), 148.26(C-3′), 135.46(C-3), 123.96(C-6′), 122.94(C-

1′), 116.07(C-5′), 114.53(C-2′), 105.65(C-10), 104.46(C-1″), 102.50(C-1‴), 99.97(C-6), 94.93(C-8), 78.14(C-3″), 77.32(C-5″), 75.89(C-2″), 73.82(C-4‴), 72.25(C-3‴), 72.05(C-2‴), 71.59(C-4″), 69.76, 68.51(C-6″), 56.75(OCH_3), 17.87(C-6‴).

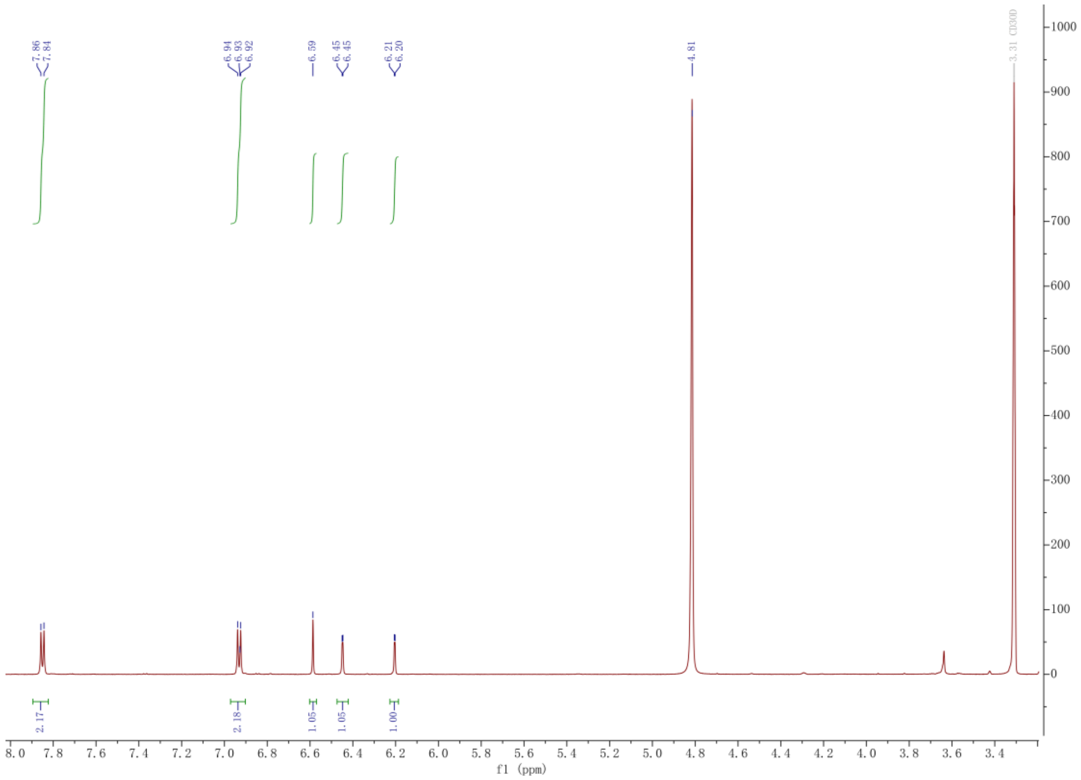

Figure S1. ^{1}H NMR spectrum of compound **1**.

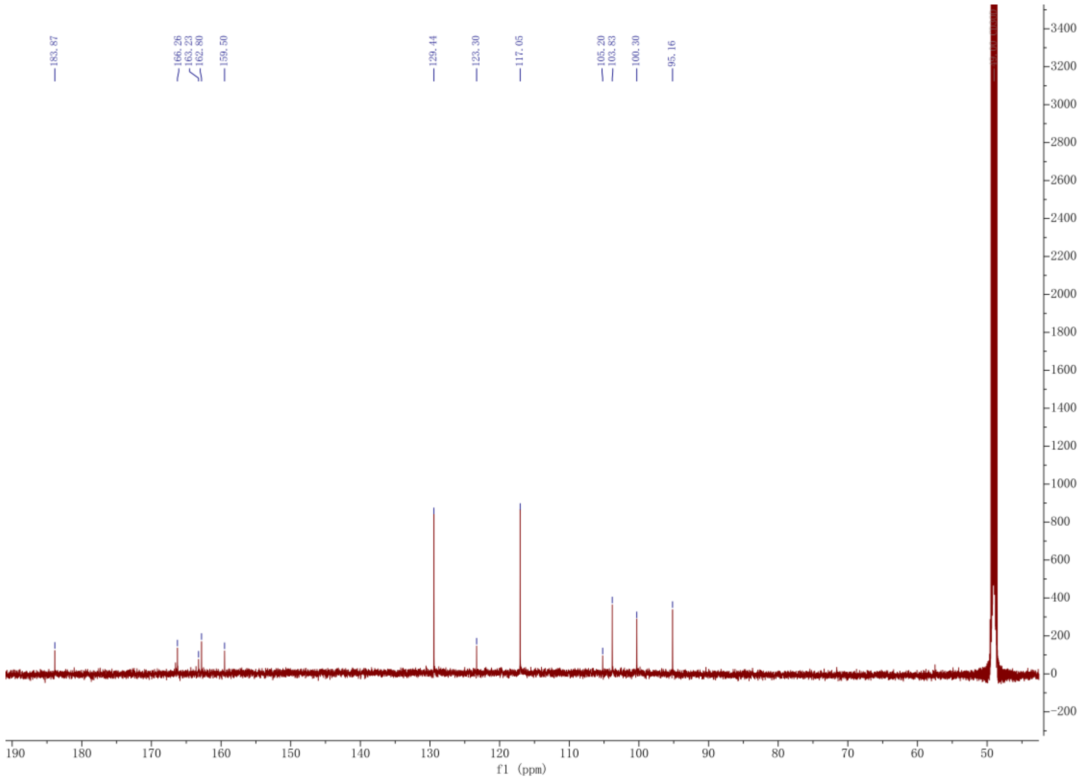

Figure S2. ^{13}C NMR spectrum of compound **1**.

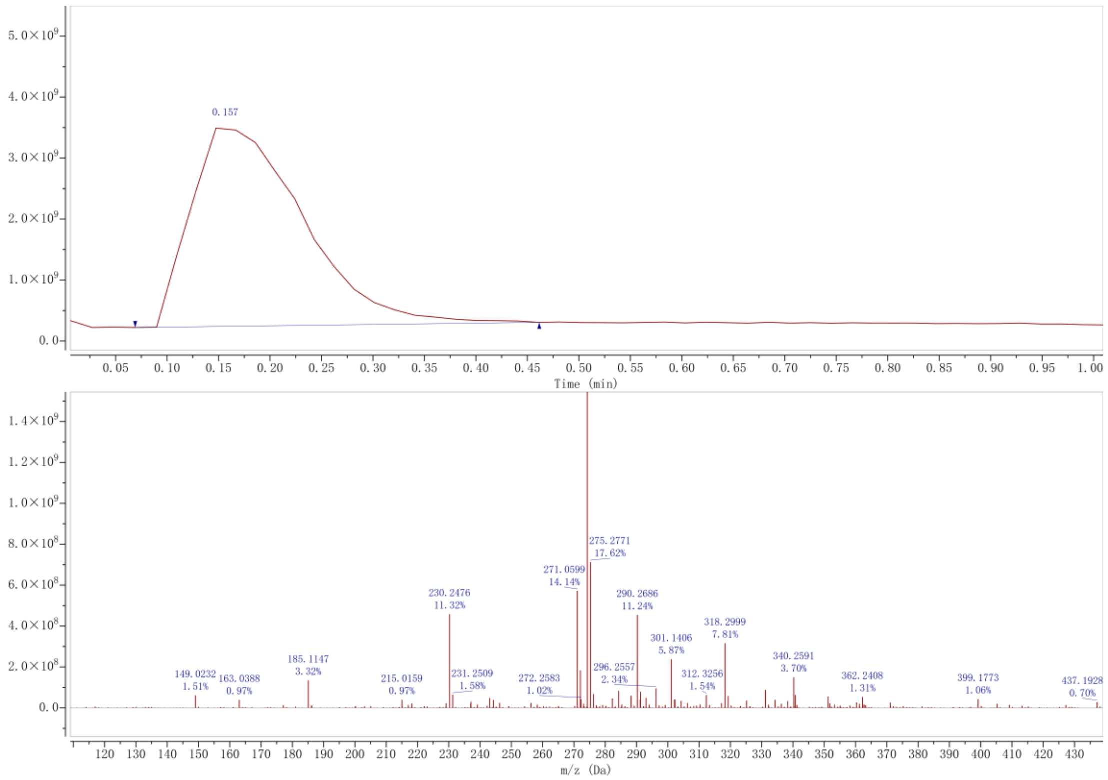

Figure S3. HRMS spectrum of compound **1**.

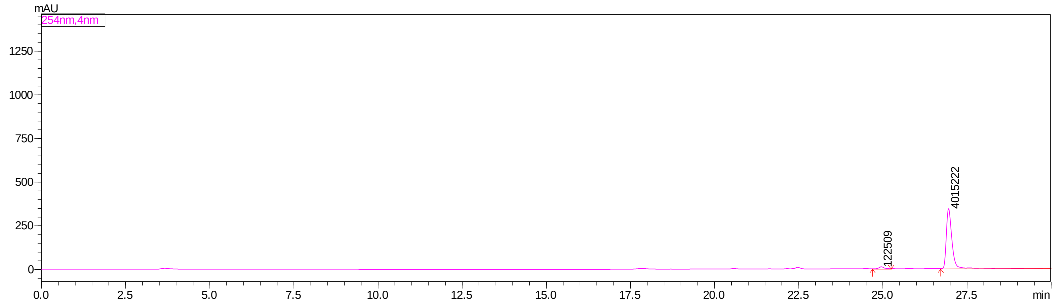

Figure S4. HPLC chromatogram of compound **1**.

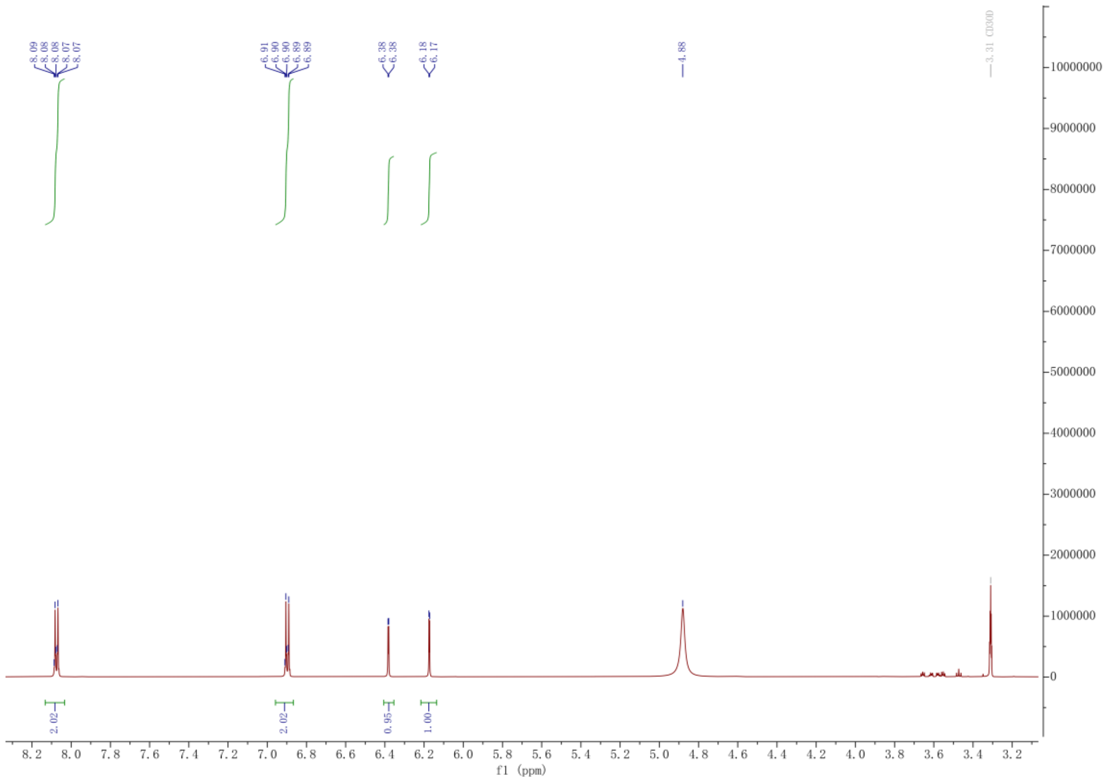

Figure S4. ^{1}H NMR spectrum of compound **2**.

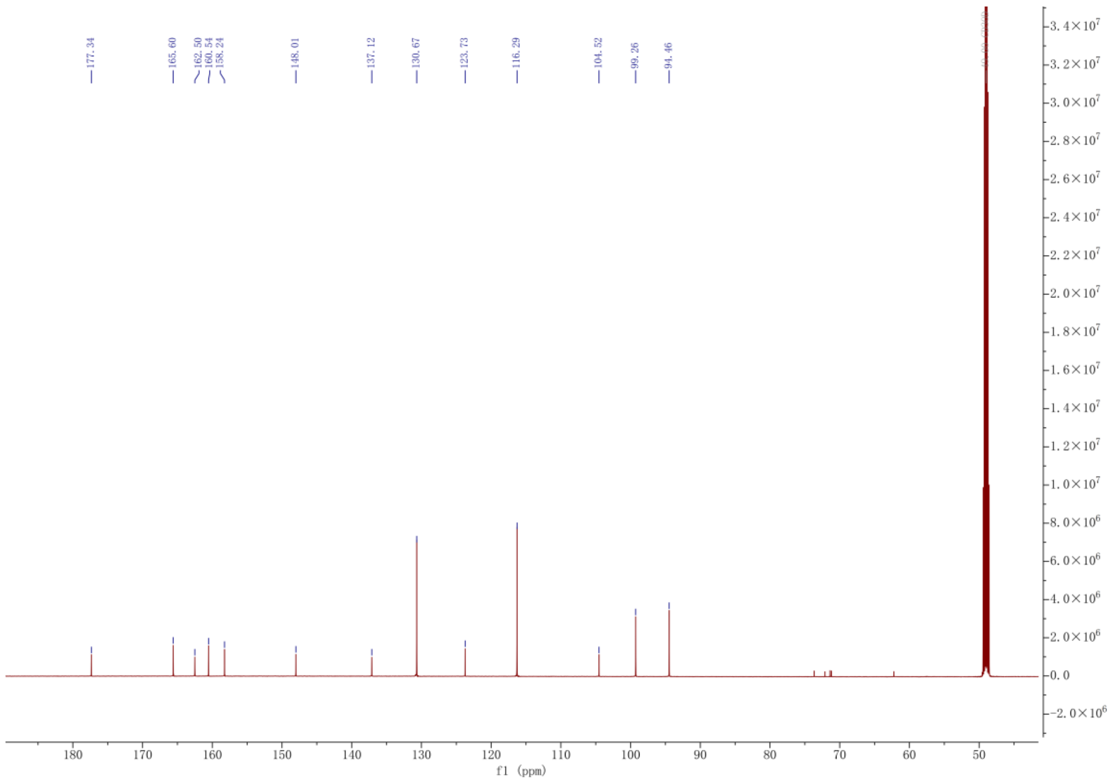

Figure S6. ^{13}C NMR spectrum of compound **2**.

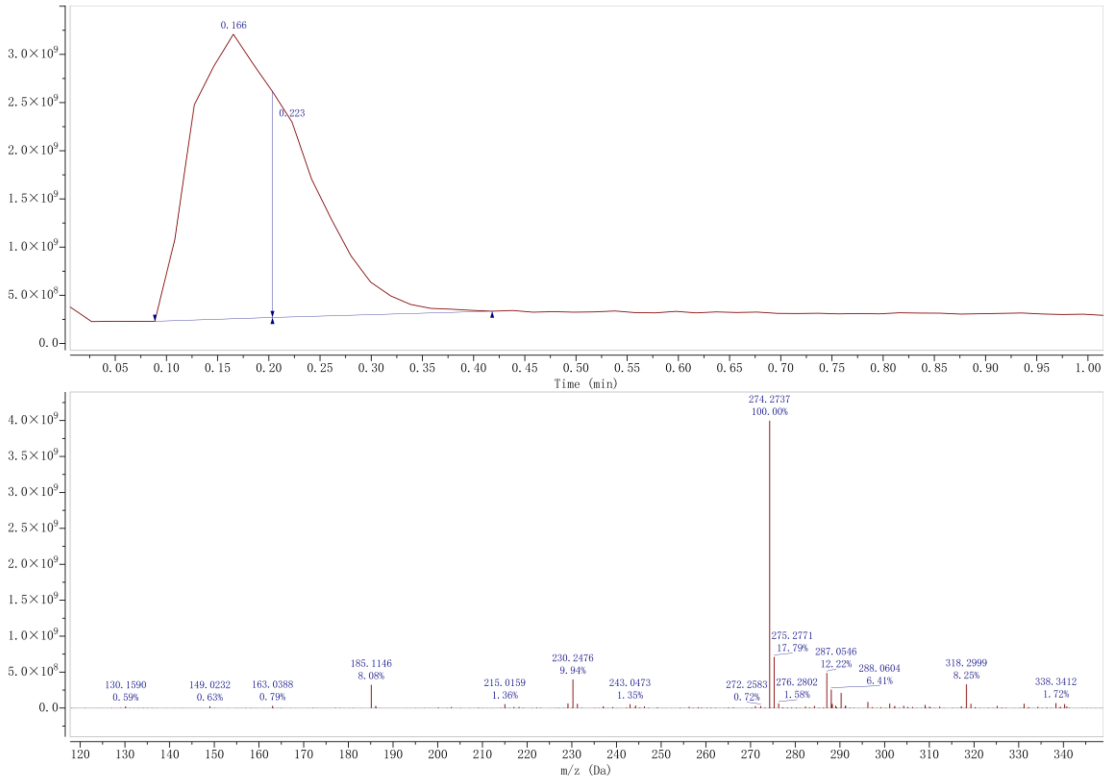

Figure S7. HRMS spectrum of compound **2**.

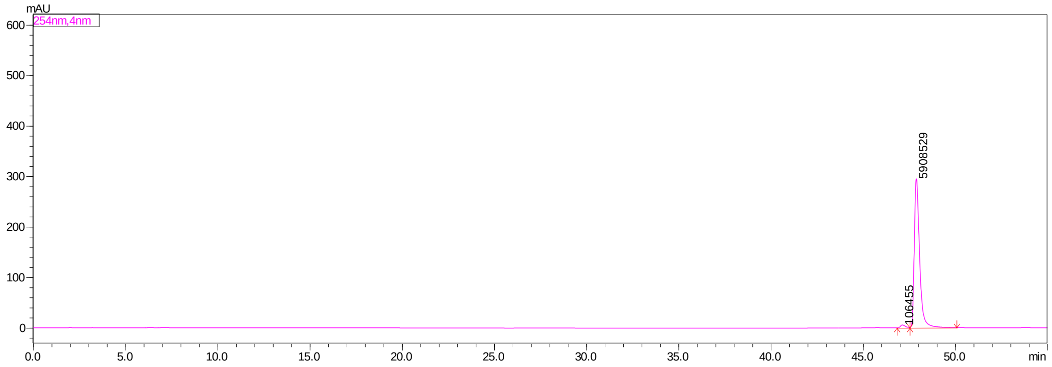

Figure S8. HPLC chromatogram of compound **2**.

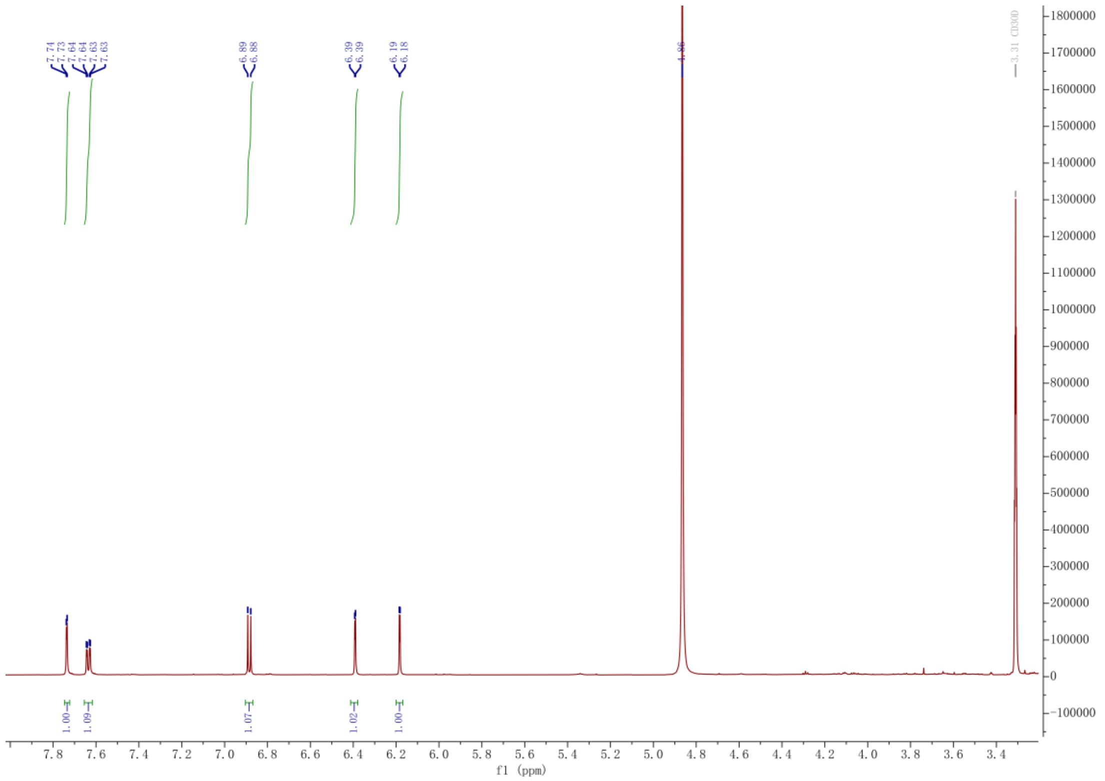

Figure S9. ^{1}H NMR spectrum of compound **3**.

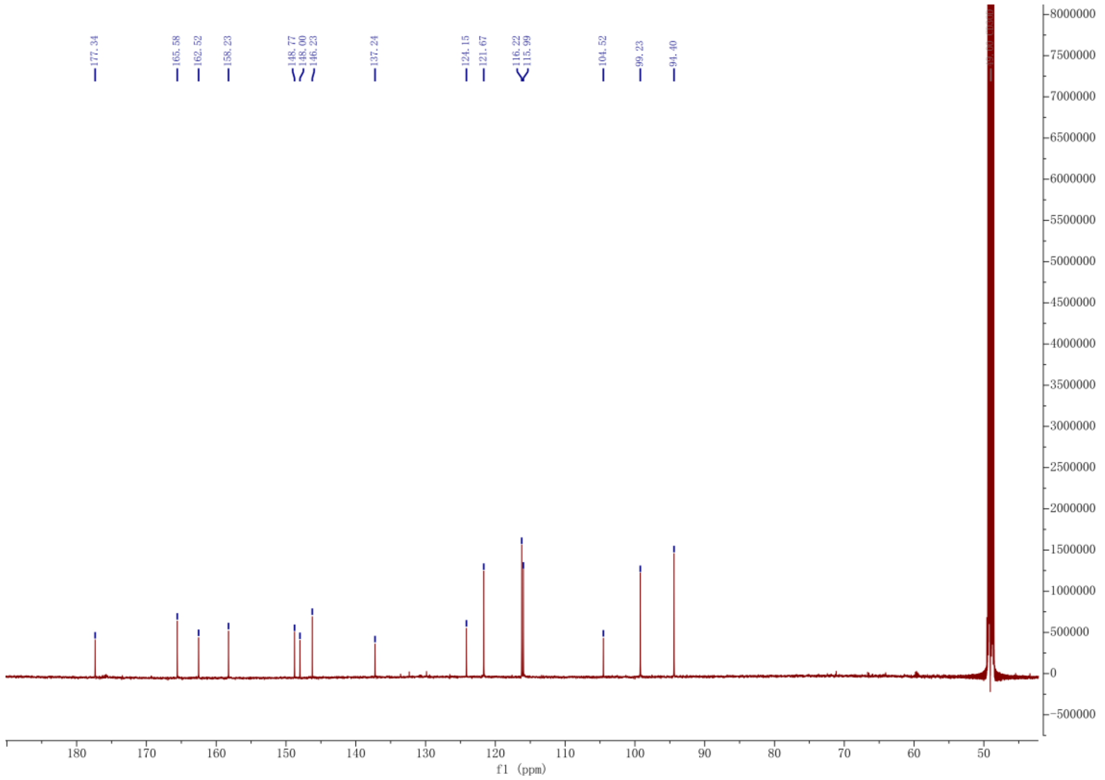

Figure S10. ^{13}C NMR spectrum of compound **3**.

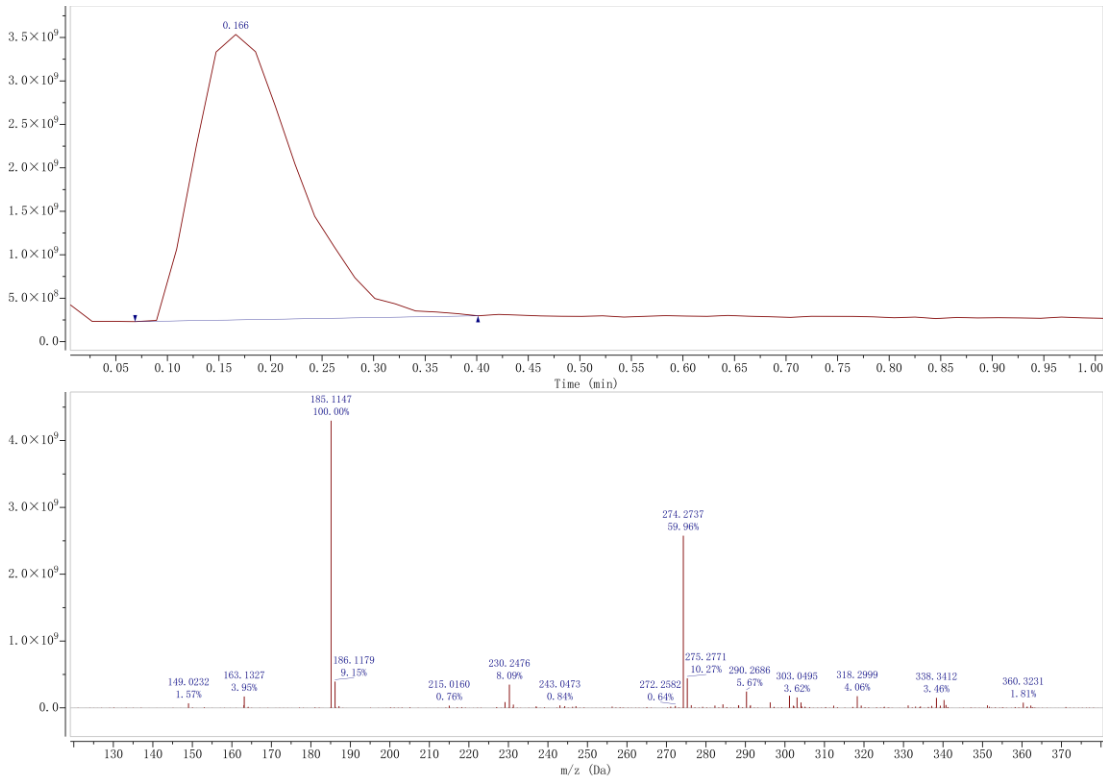

Figure S11. HRMS spectrum of compound **3**.

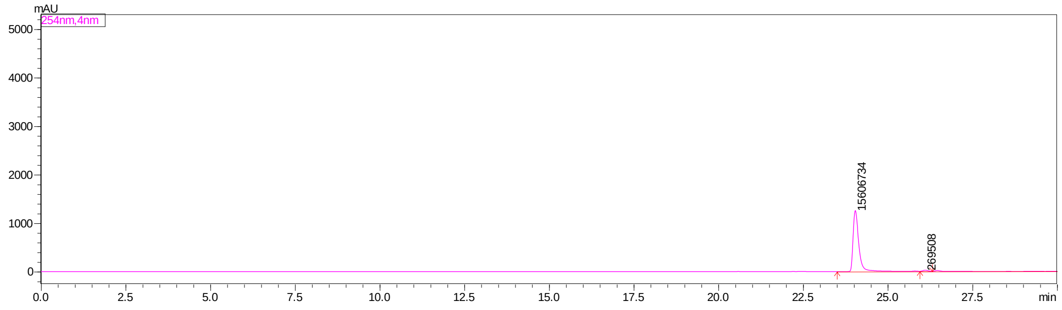

Figure S12. HPLC chromatogram of compound **3**.

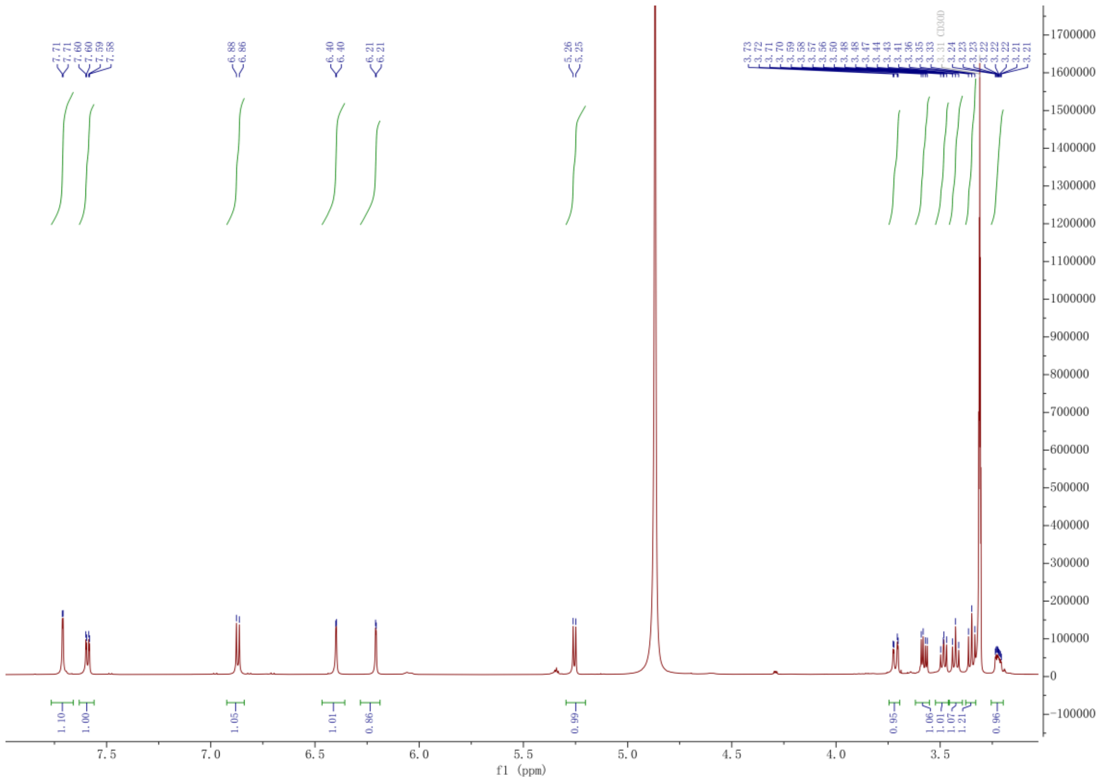

Figure S13. ^{1}H NMR spectrum of compound **4**.

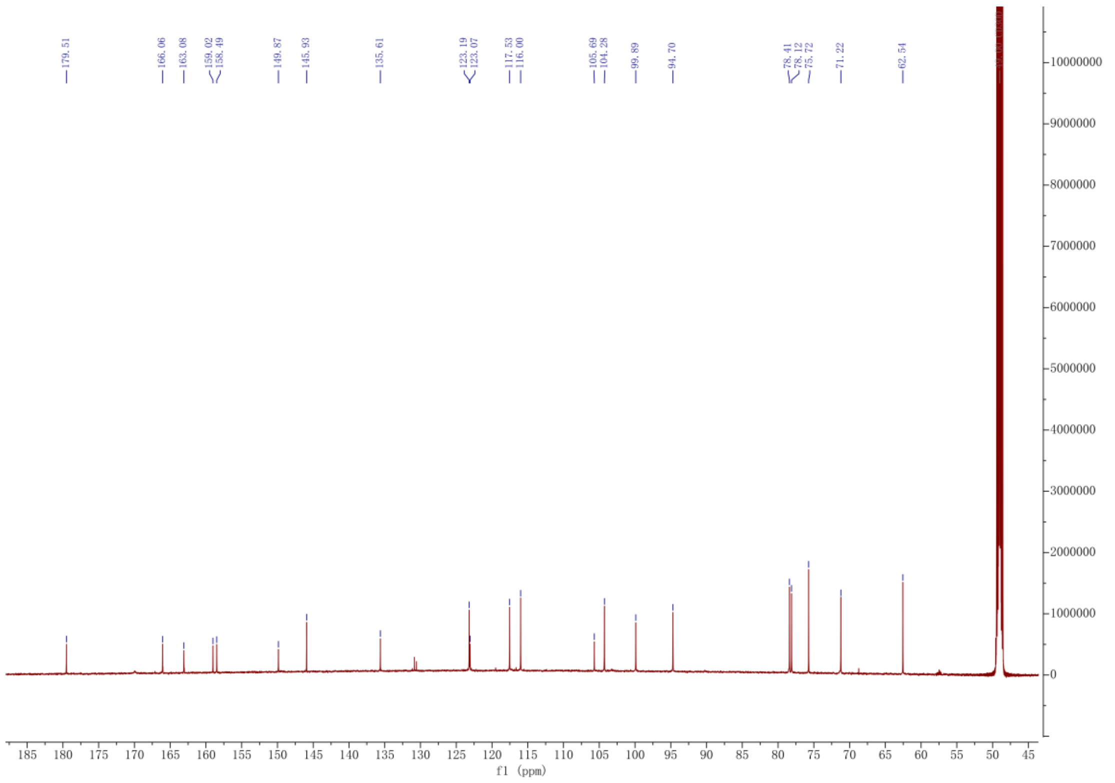

Figure S14. ^{13}C NMR spectrum of compound **4**.

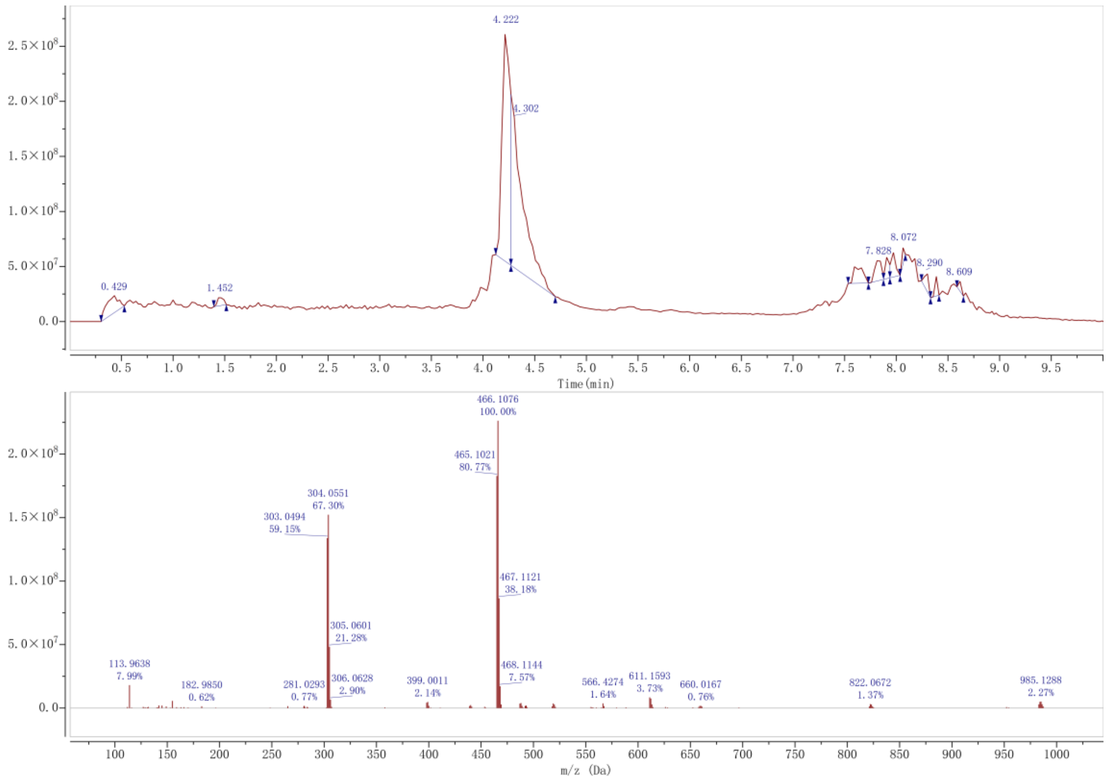

Figure S15. HRMS spectrum of compound **4**.

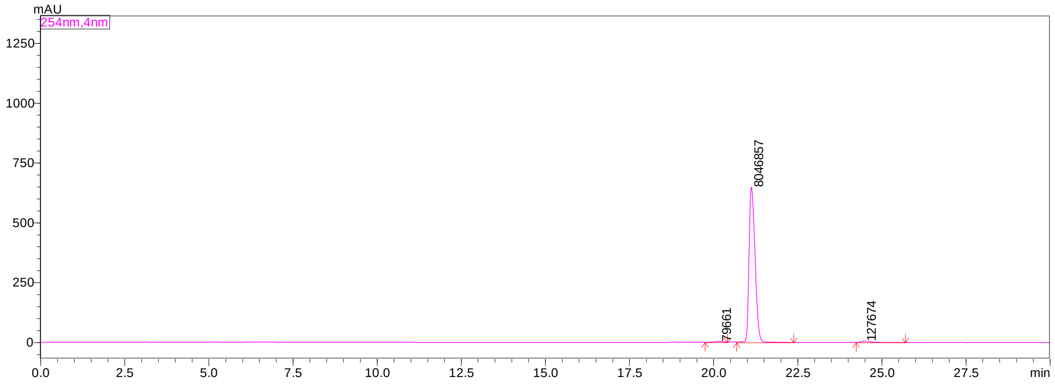

Figure S16. HPLC chromatogram of compound **4**.

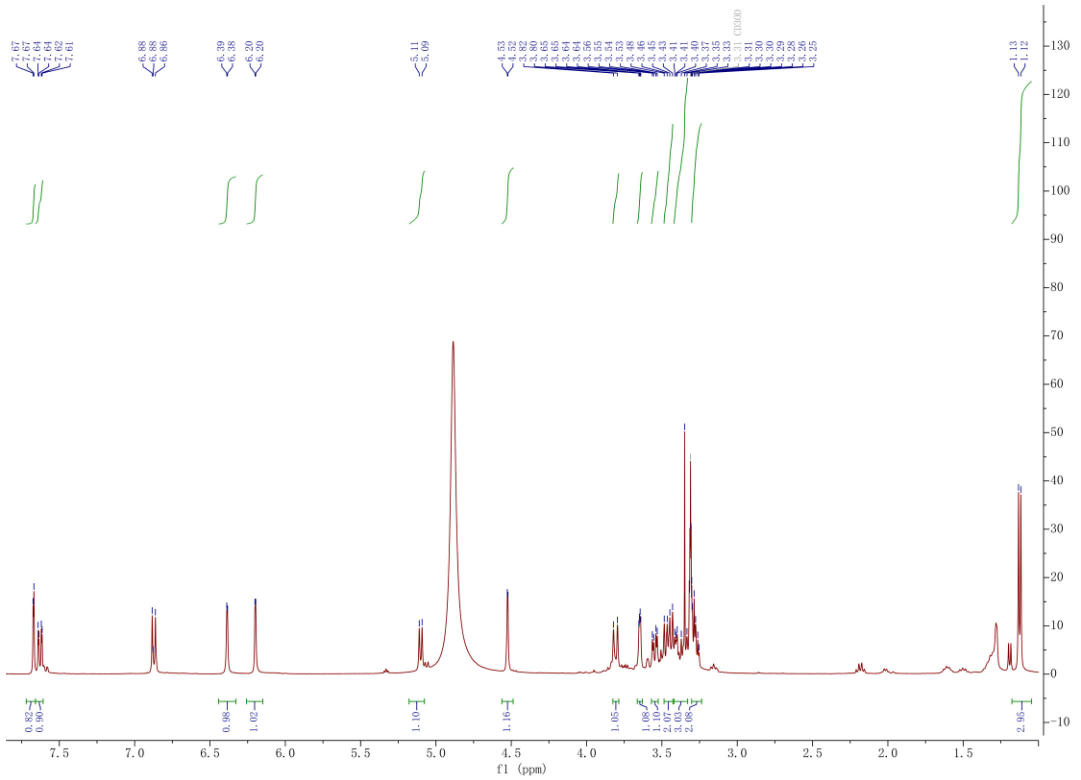

Figure S17. ^{1}H NMR spectrum of compound **5**.

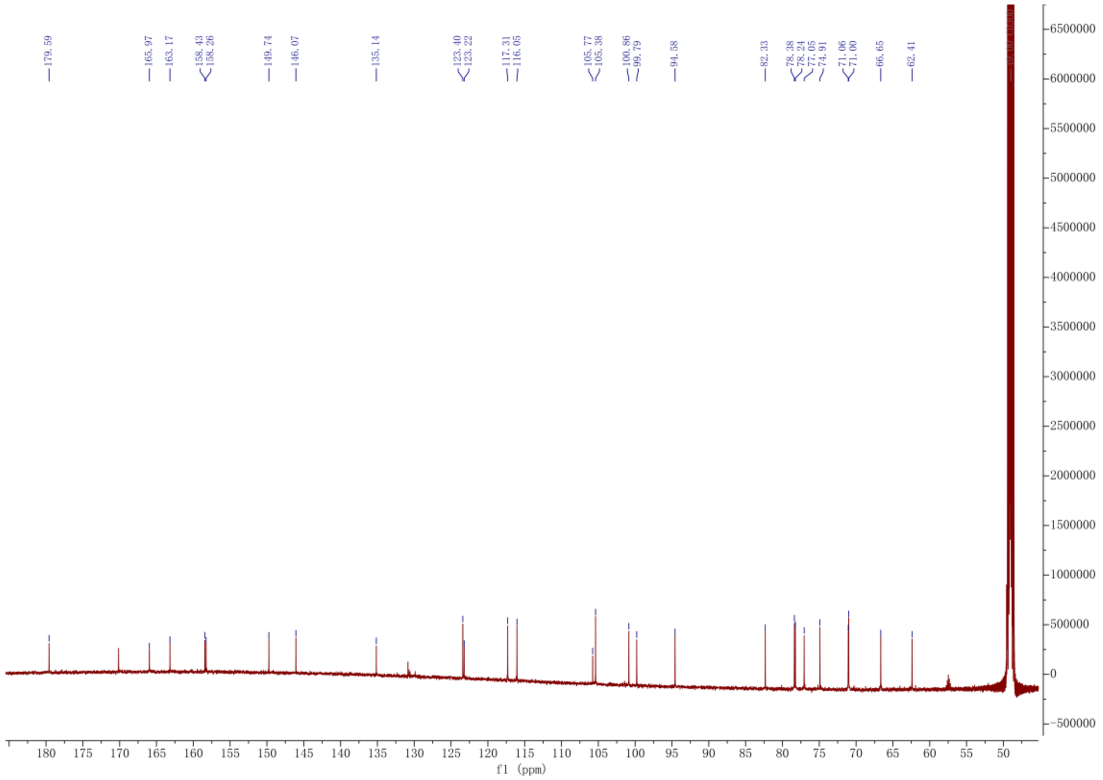

Figure S18. ^{13}C NMR spectrum of compound **5**.

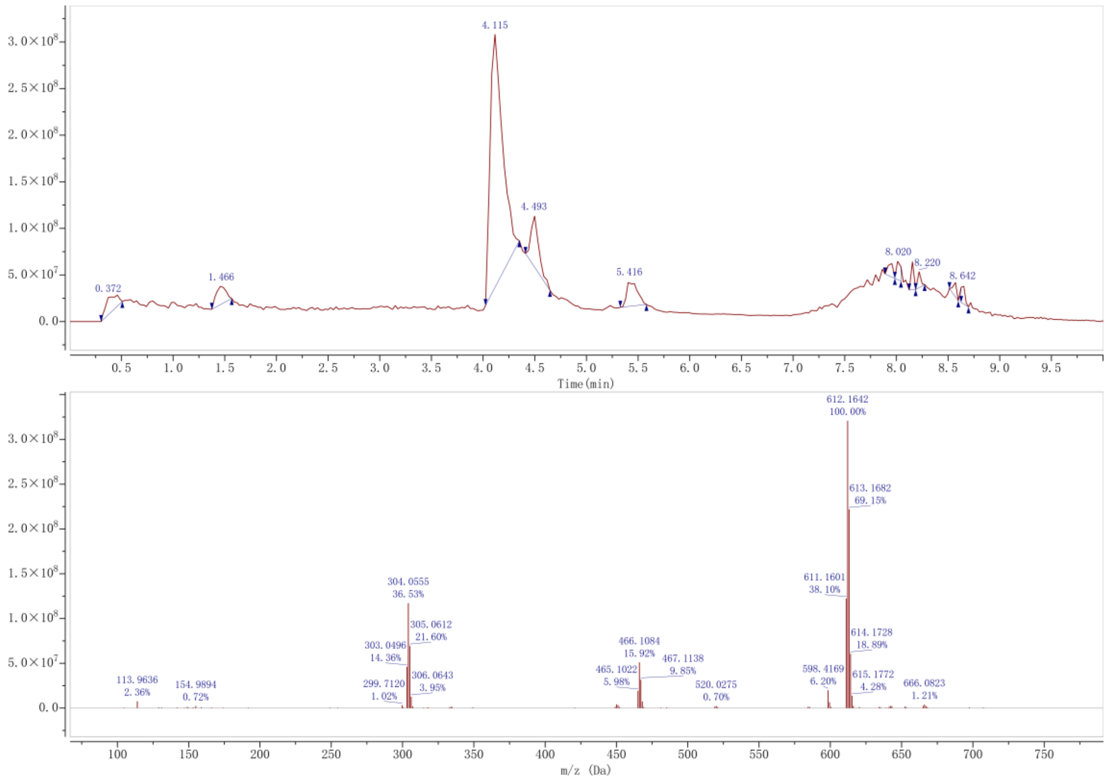

Figure S19. HRMS spectrum of compound **5**.

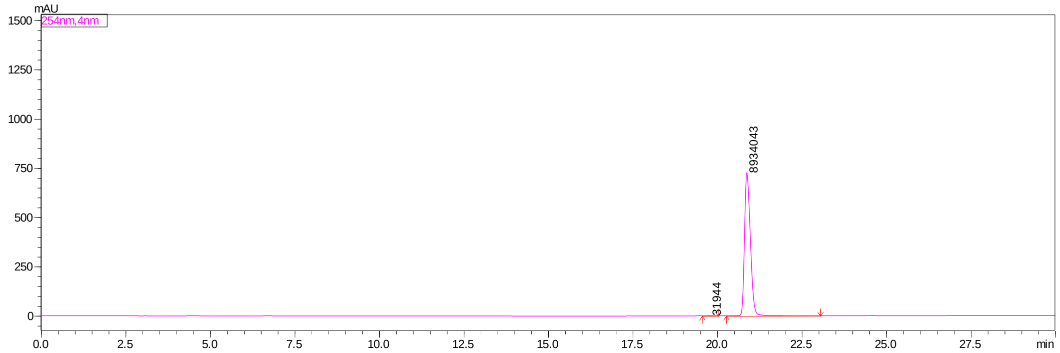

Figure S10. HPLC chromatogram of compound **5**.

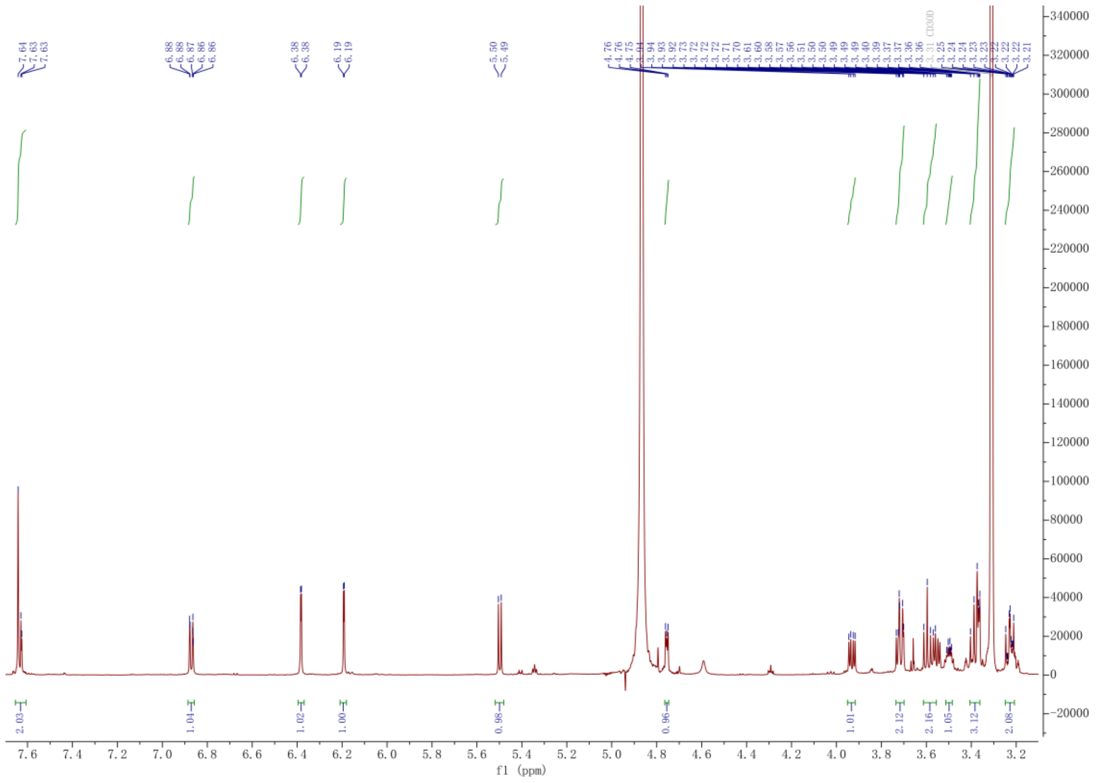

Figure S21. ^{1}H NMR spectrum of compound **6**.

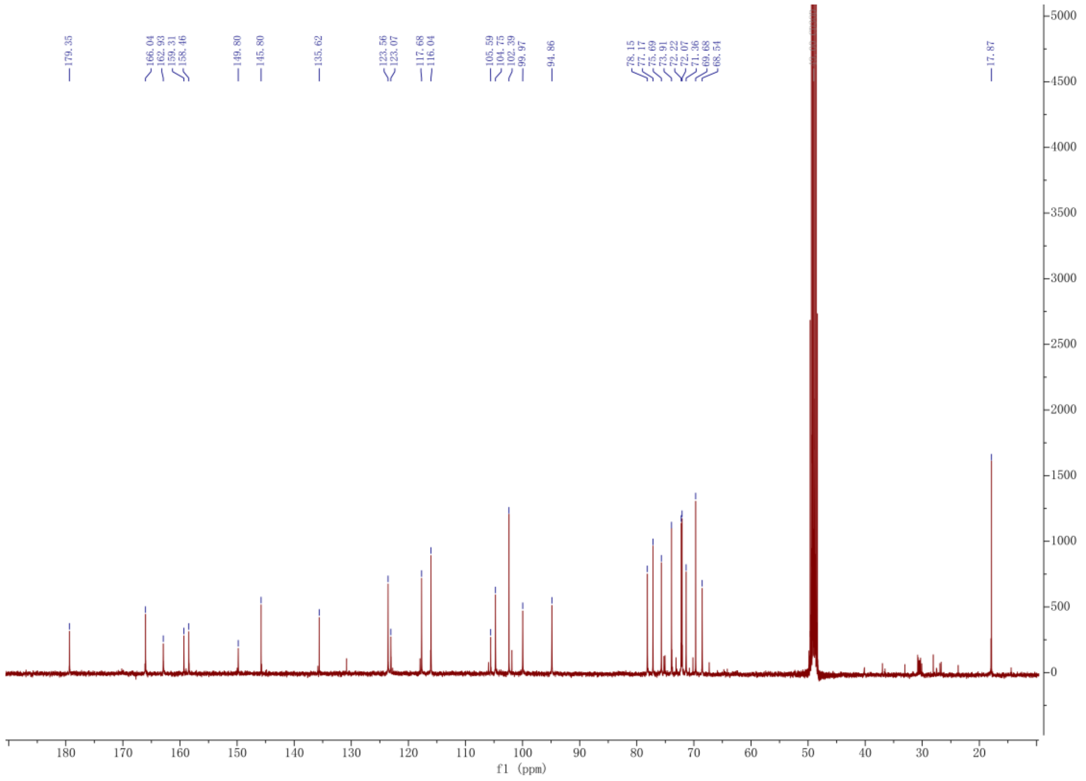

Figure S22. ^{13}C NMR spectrum of compound **6**.

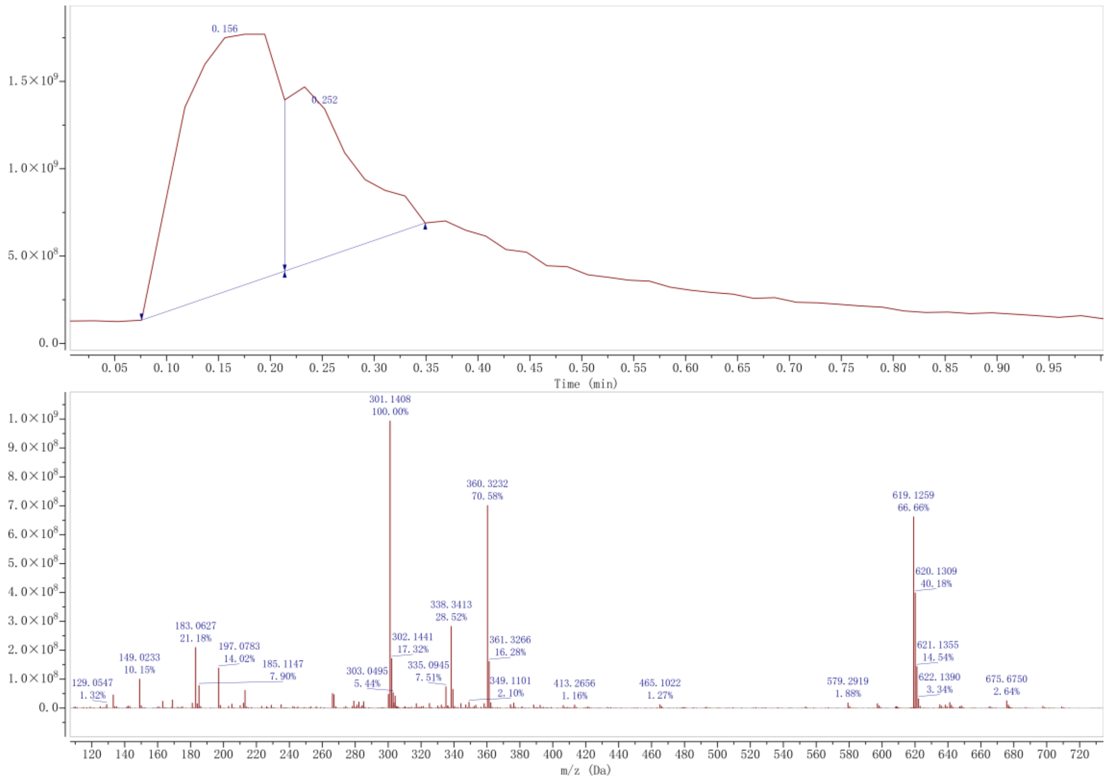

Figure S23. HRMS spectrum of compound **6**.

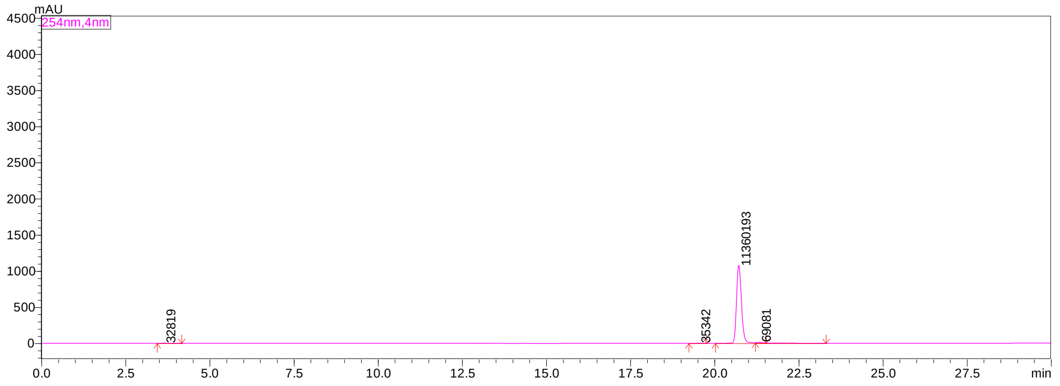

Figure S24. HPLC chromatogram of compound **6**.

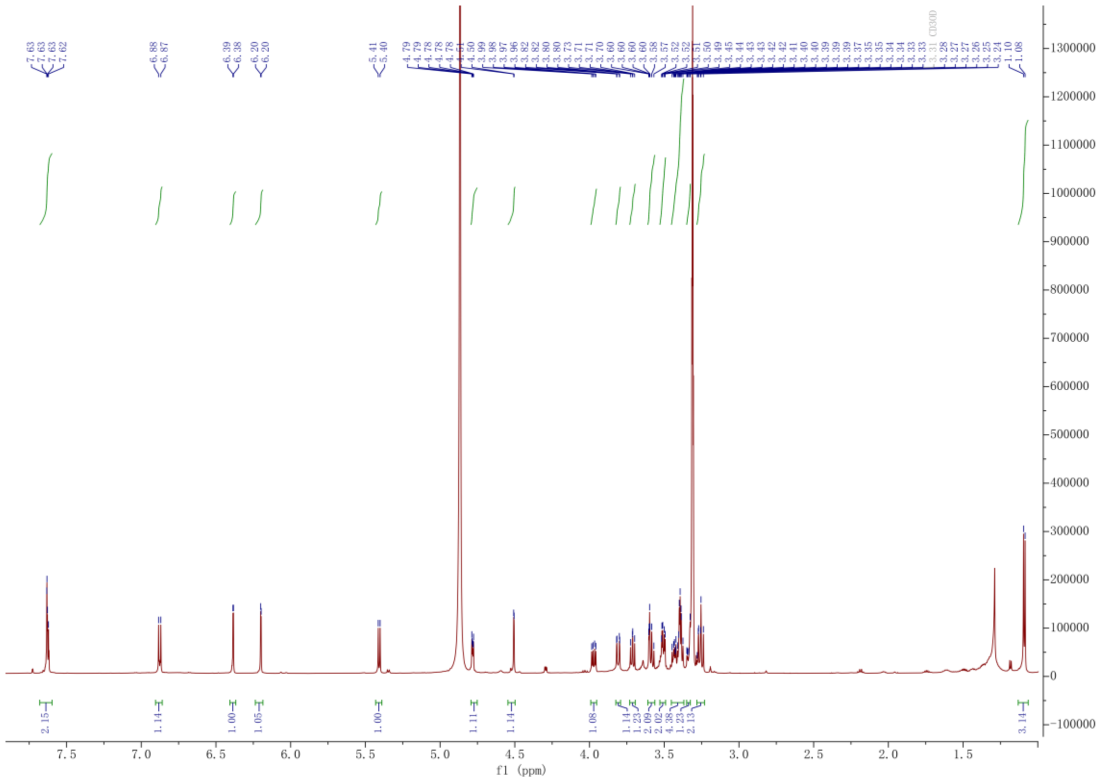

Figure S25. ^{1}H NMR spectrum of compound **7**.

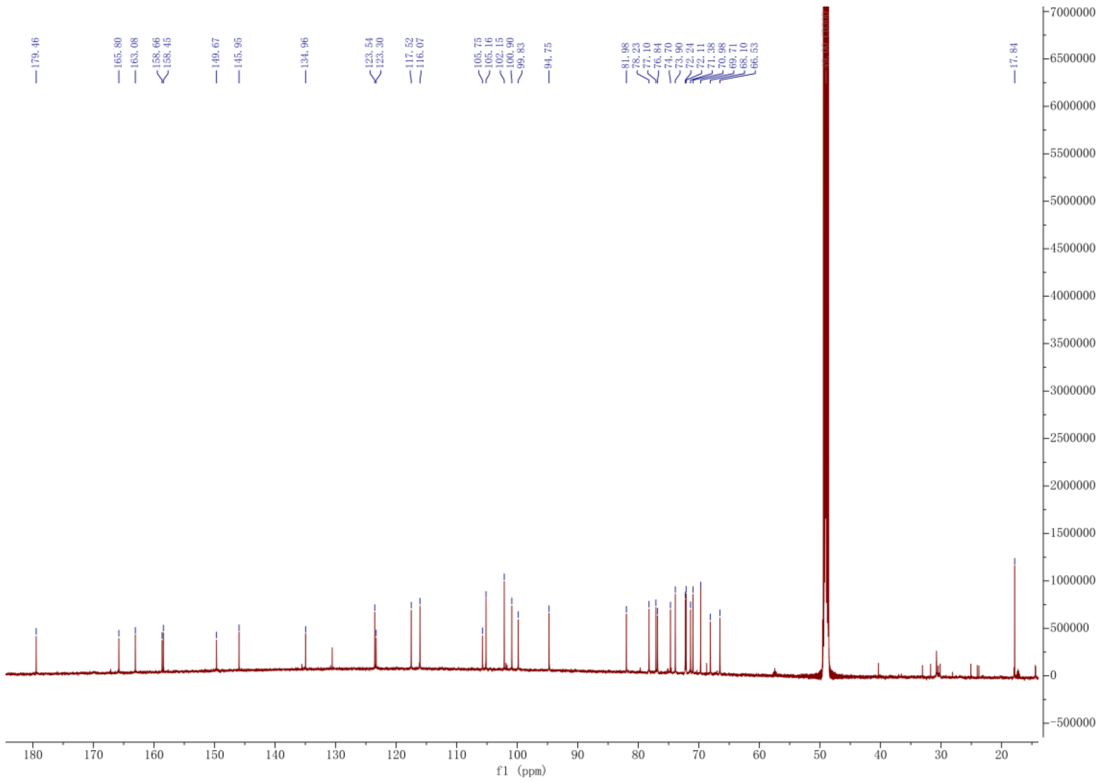

Figure S26. ^{13}C NMR spectrum of compound **7**.

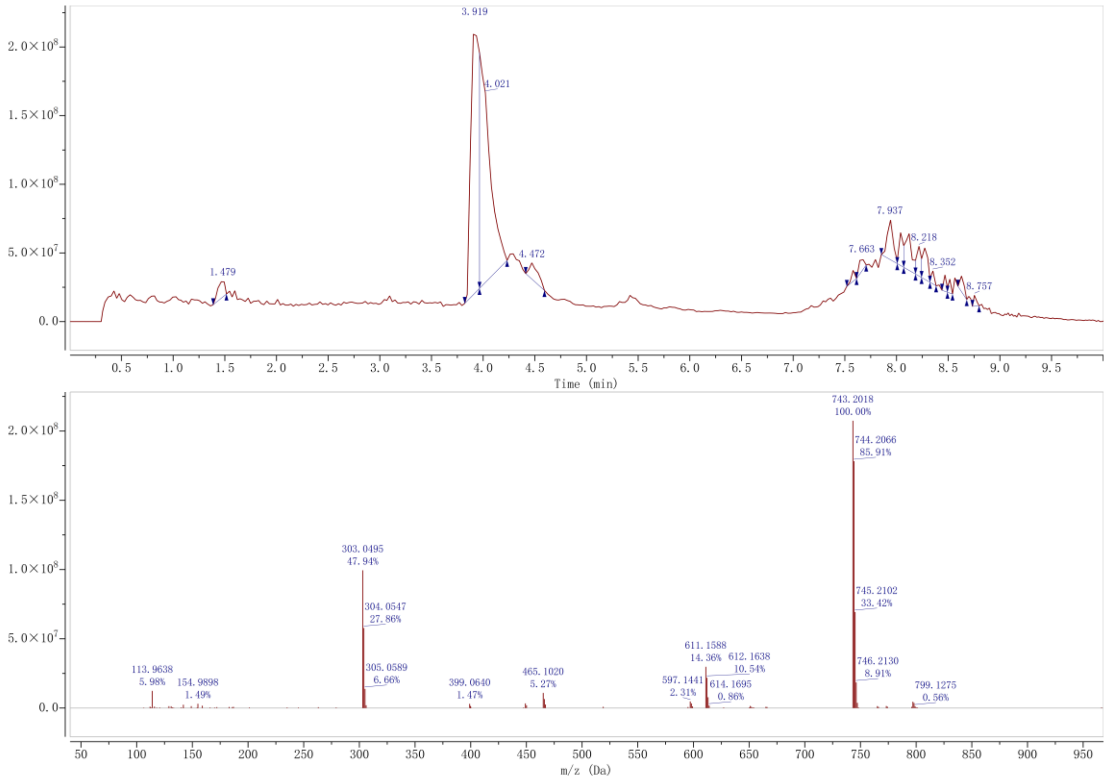

Figure S27. HRMS spectrum of compound **7**.

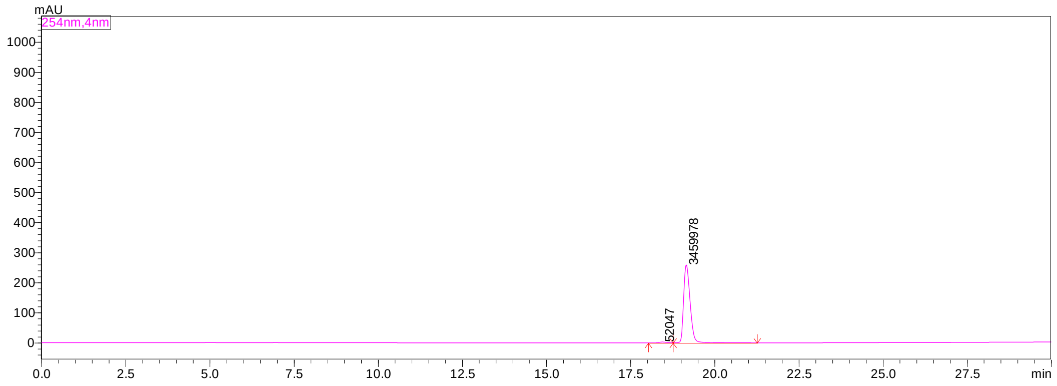

Figure S28. HPLC chromatogram of compound **7**.

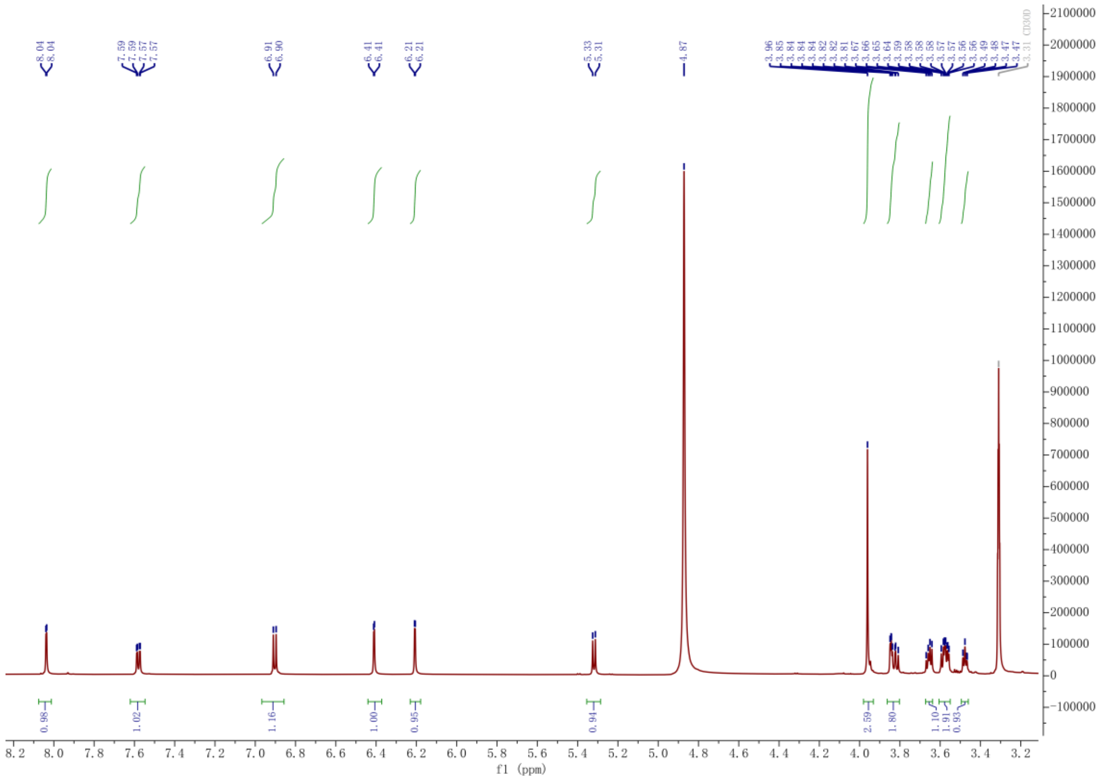

Figure S29. ^{1}H NMR spectrum of compound **8**.

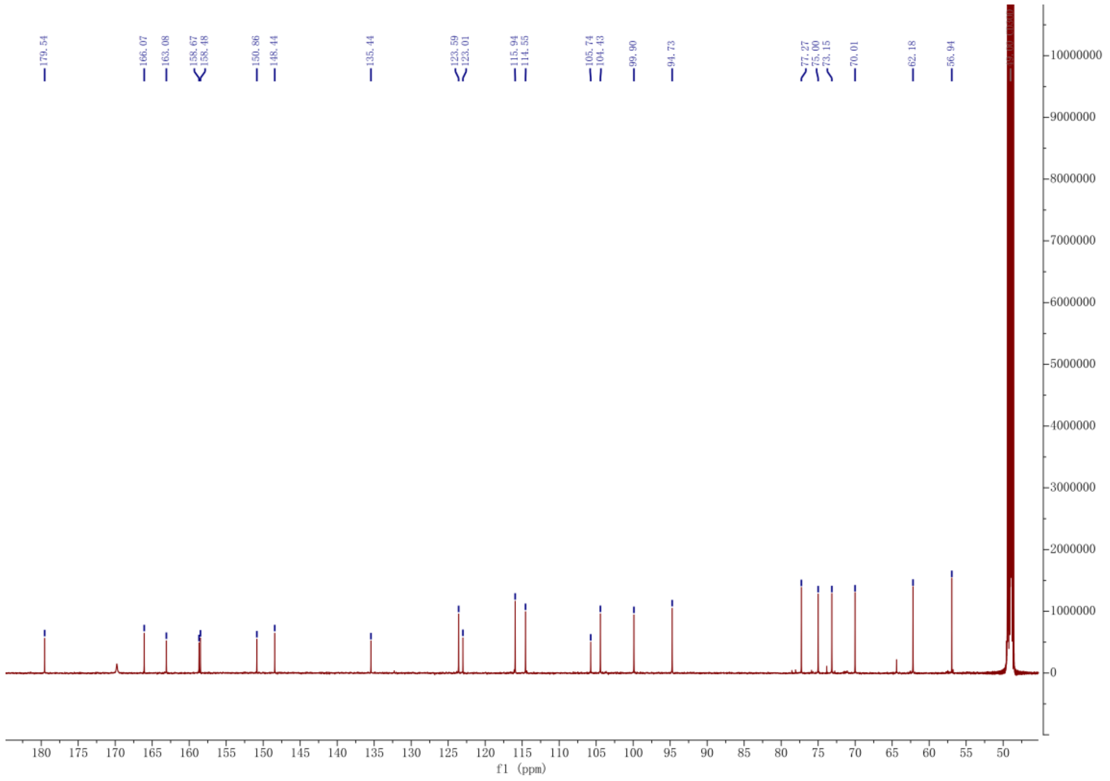

Figure S30. ^{13}C NMR spectrum of compound **8**.

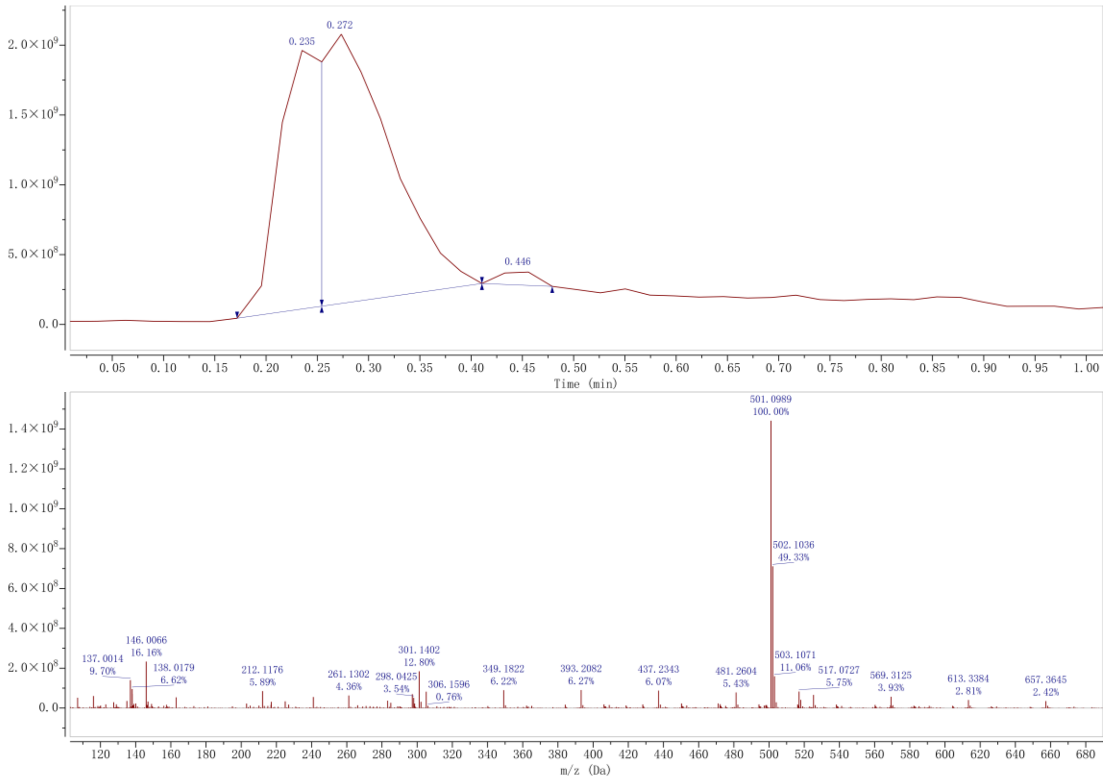

Figure S31. HRMS spectrum of compound **8**.

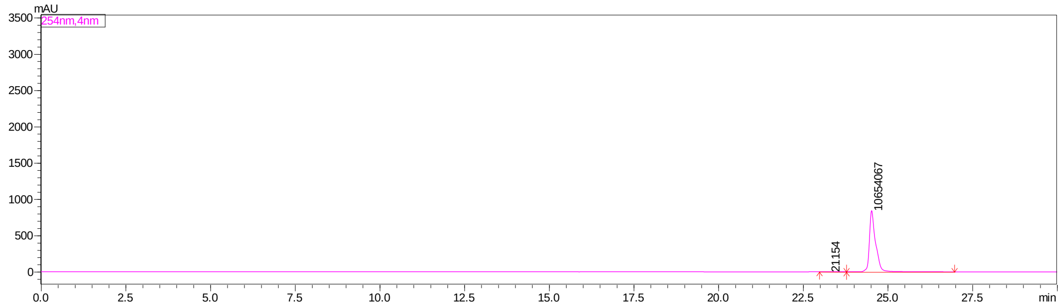

Figure S32. HPLC chromatogram of compound **8**.

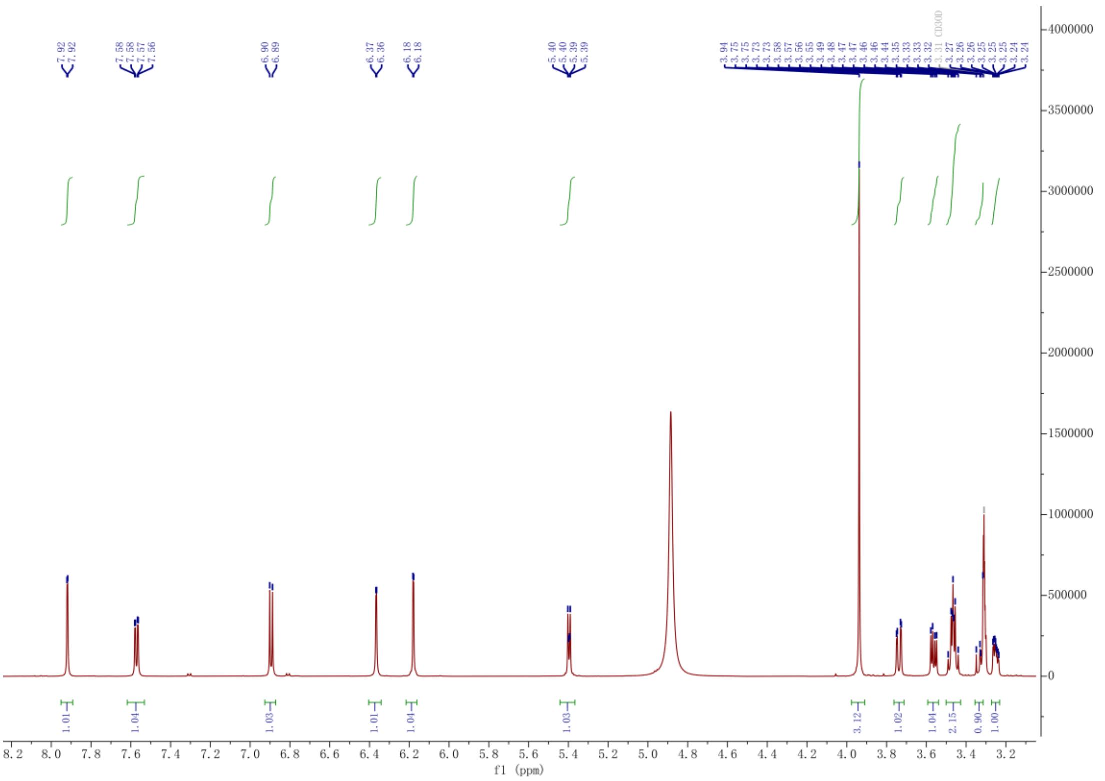

Figure S33. ^{1}H NMR spectrum of compound **9**.

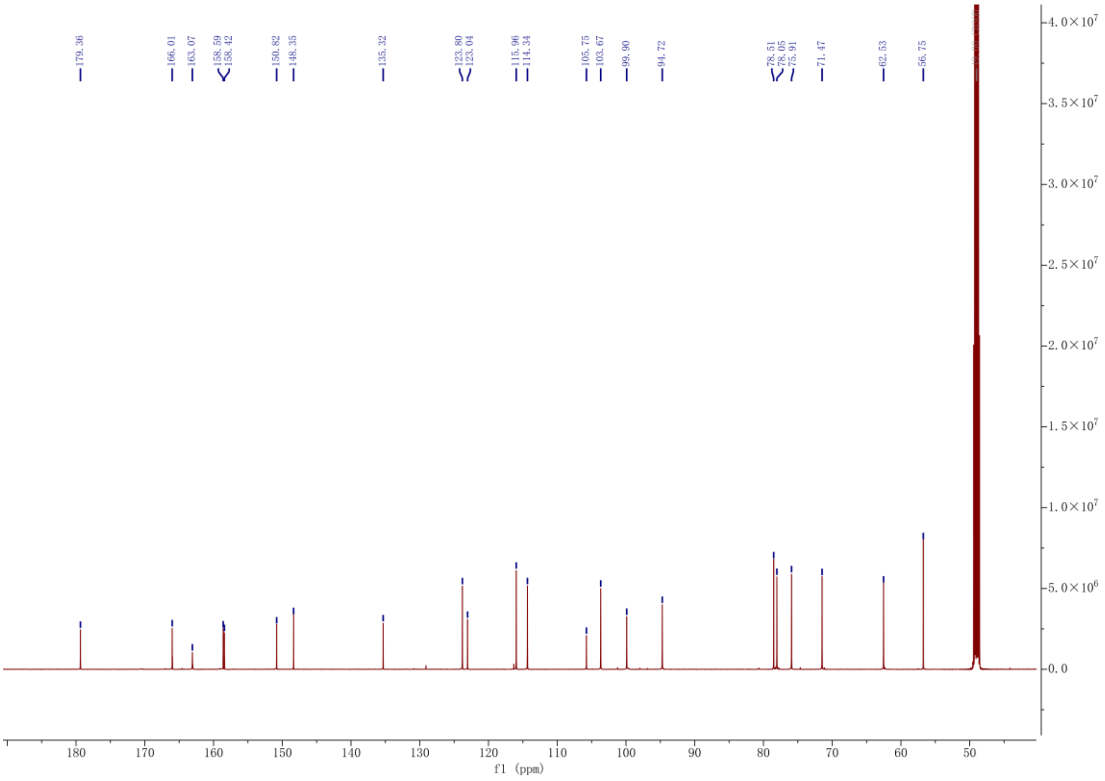

Figure S34. ^{13}C NMR spectrum of compound **9**.

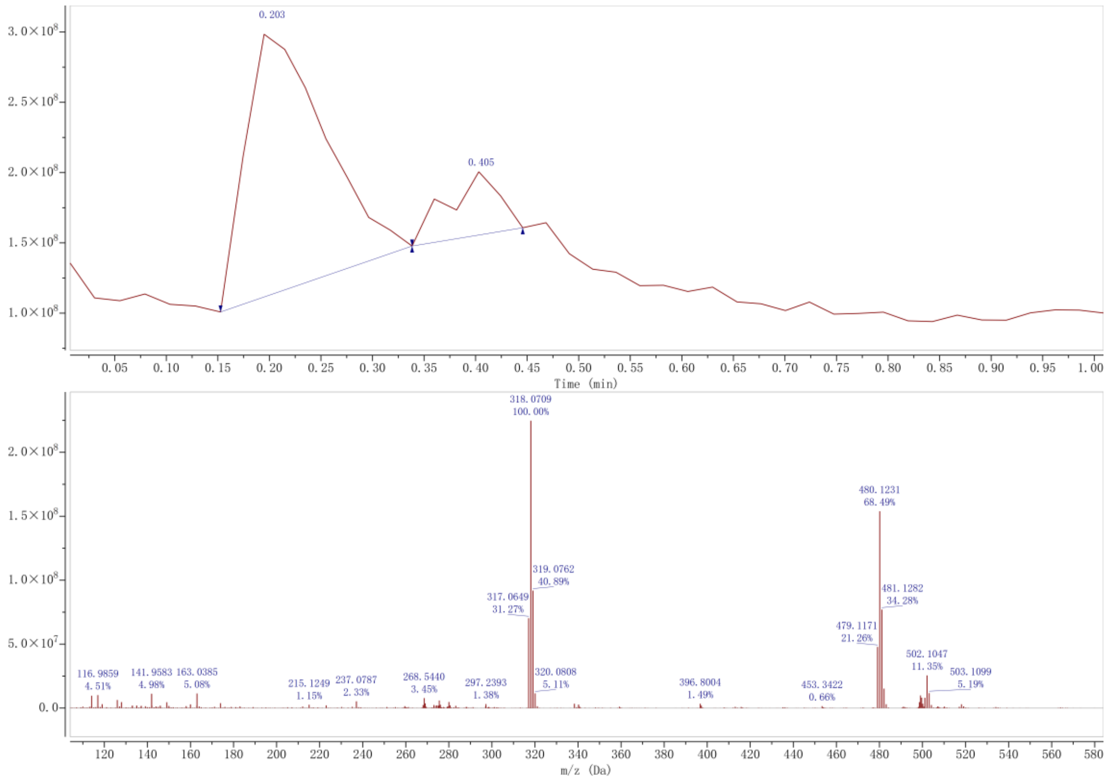

Figure S35. HRMS spectrum of compound **9**.

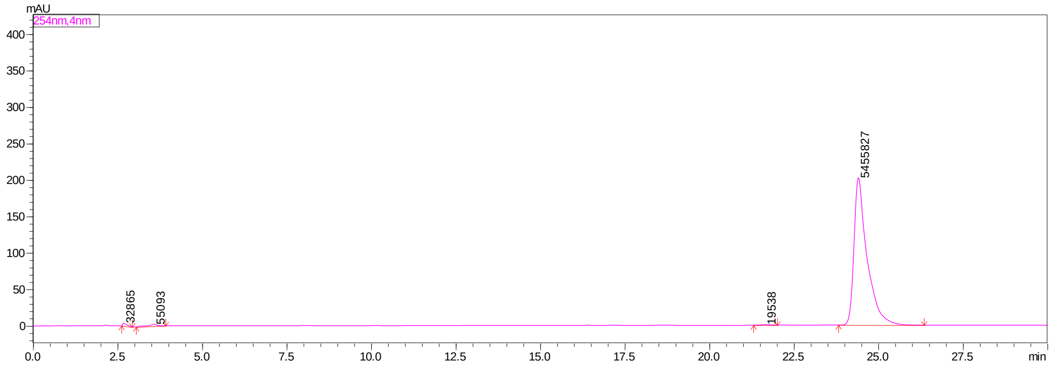

Figure S36. HPLC chromatogram of compound **9**.

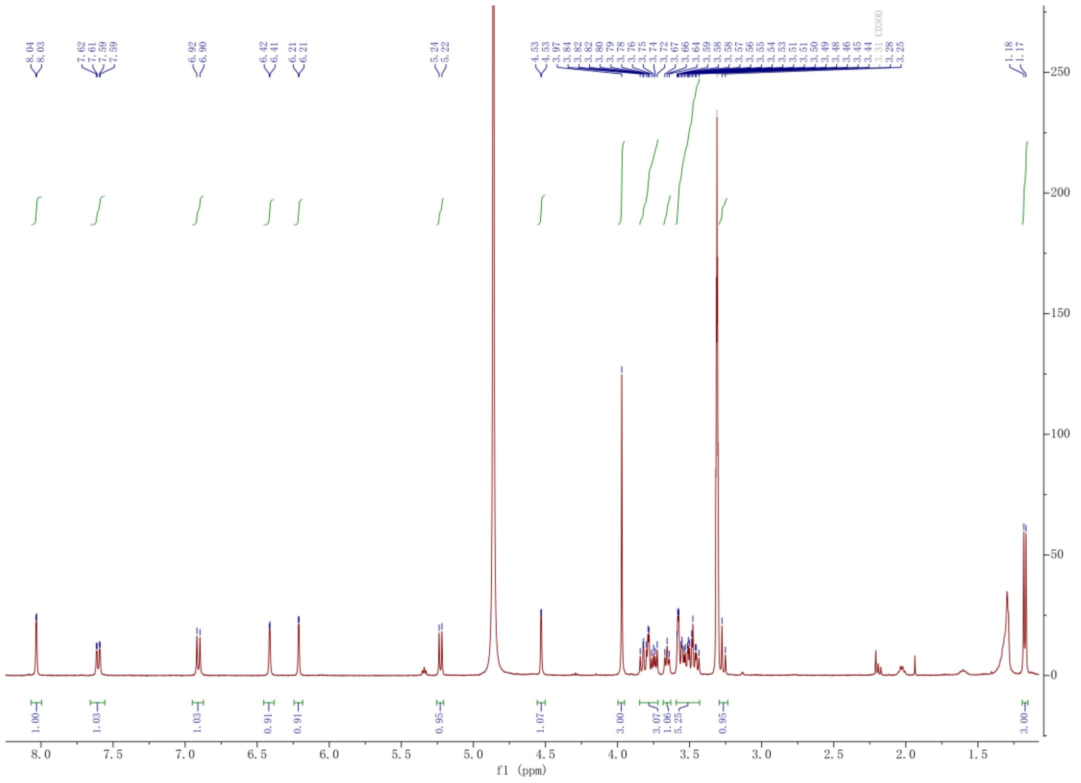

Figure S37. ^{1}H NMR spectrum of compound **10**.

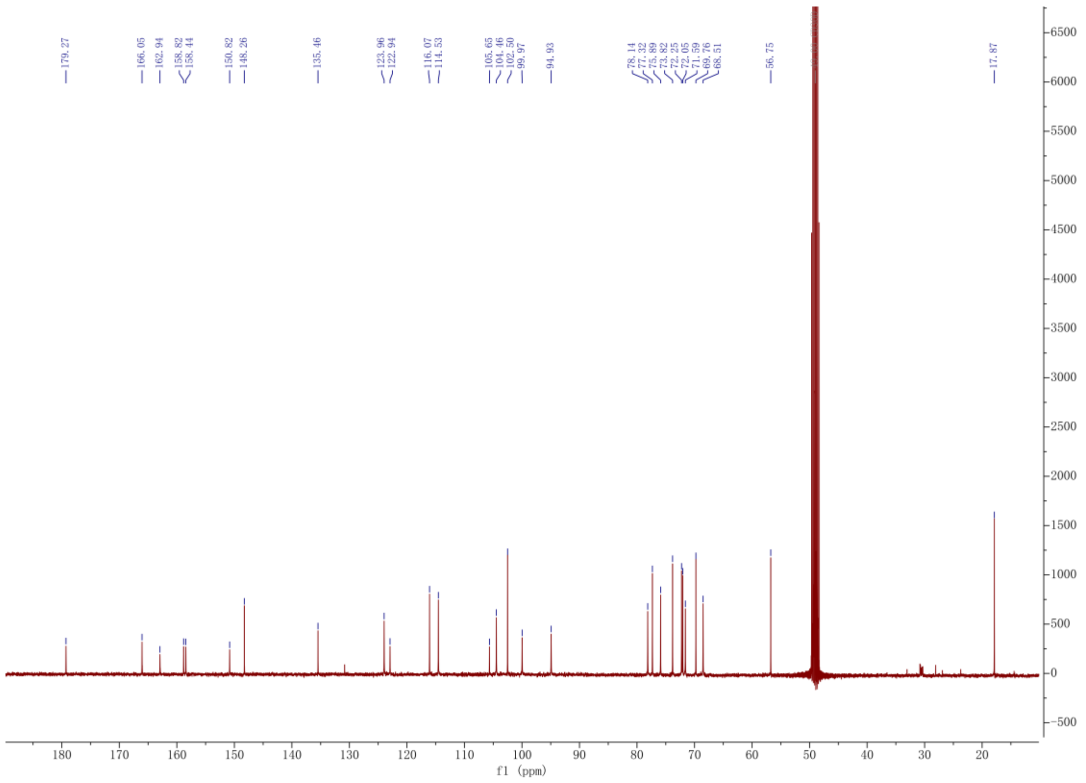

Figure S38. ^{13}C NMR spectrum of compound **10**.

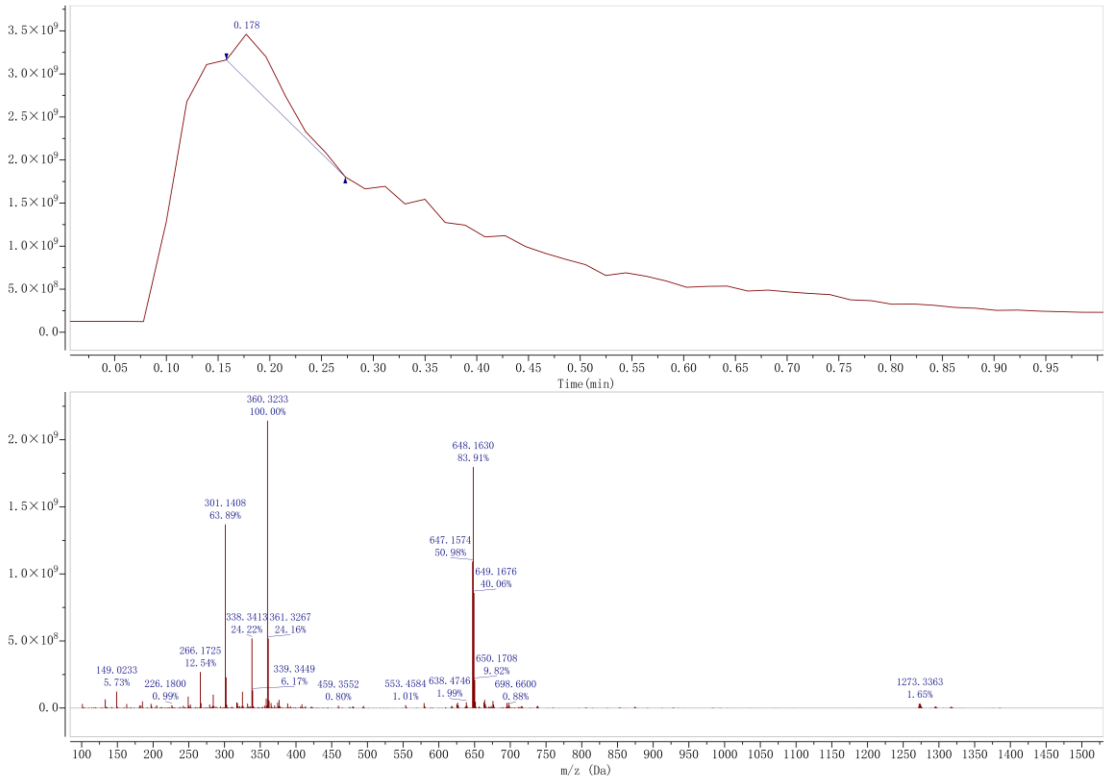

Figure S39. HRMS spectrum of compound **10**.

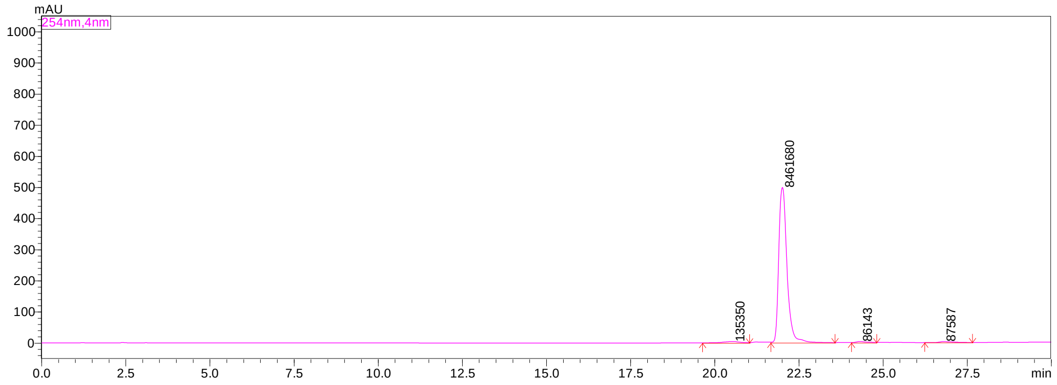

Figure S40. HPLC chromatogram of compound **10**.

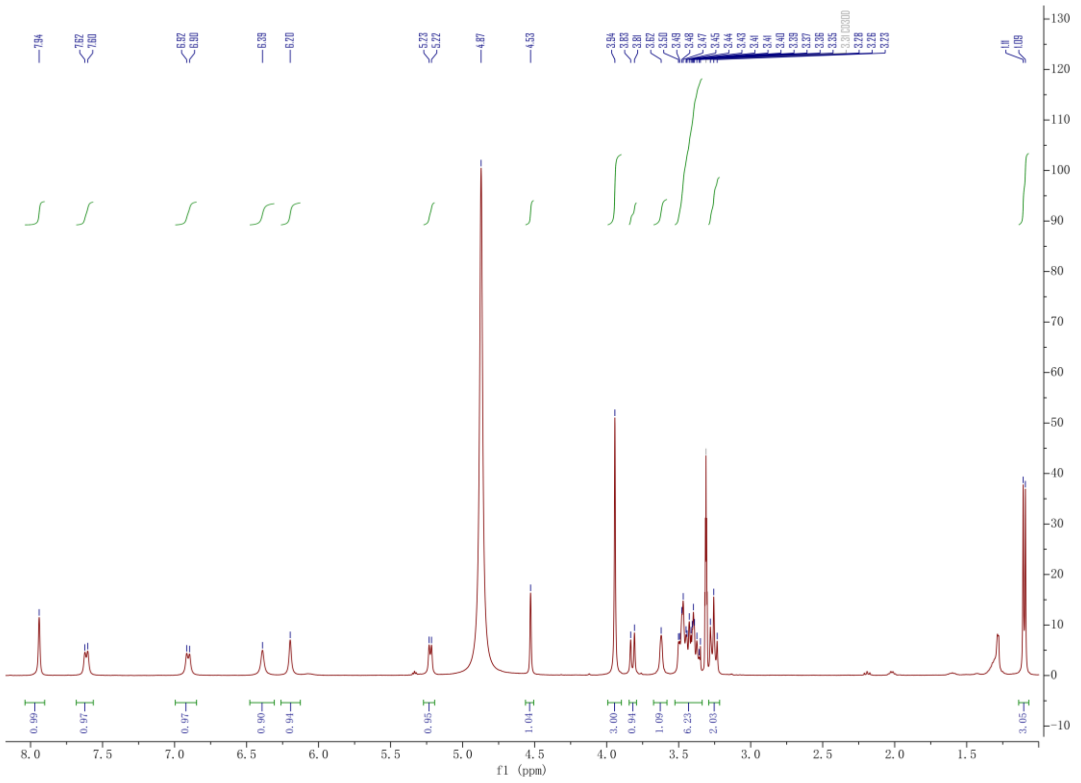

Figure S41. ¹H NMR spectrum of compound **11**.

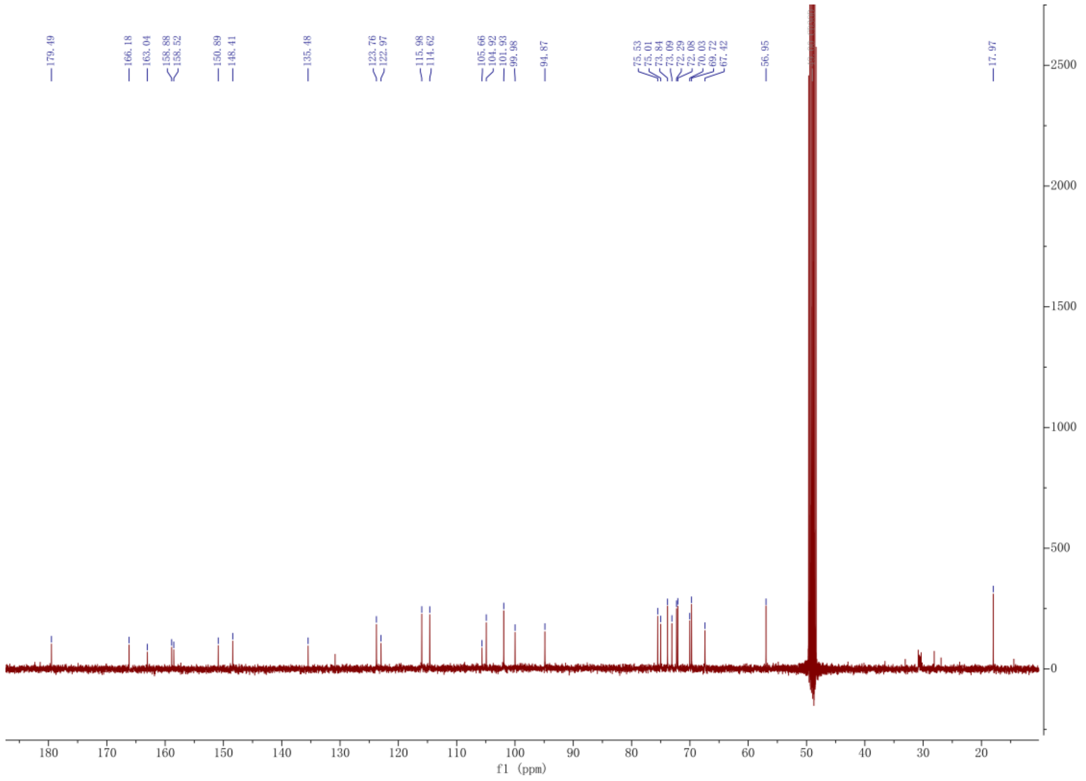

Figure S42. ¹³C NMR spectrum of compound **11**

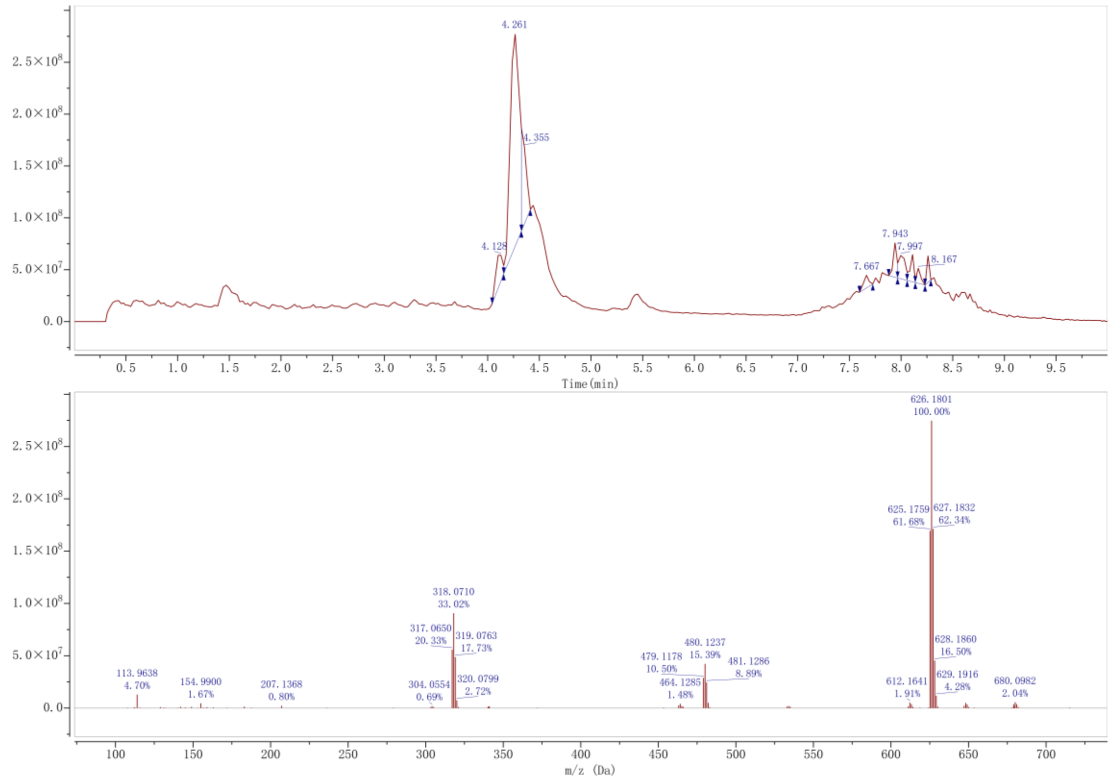

Figure S43. HRMS spectrum of compound **11**.

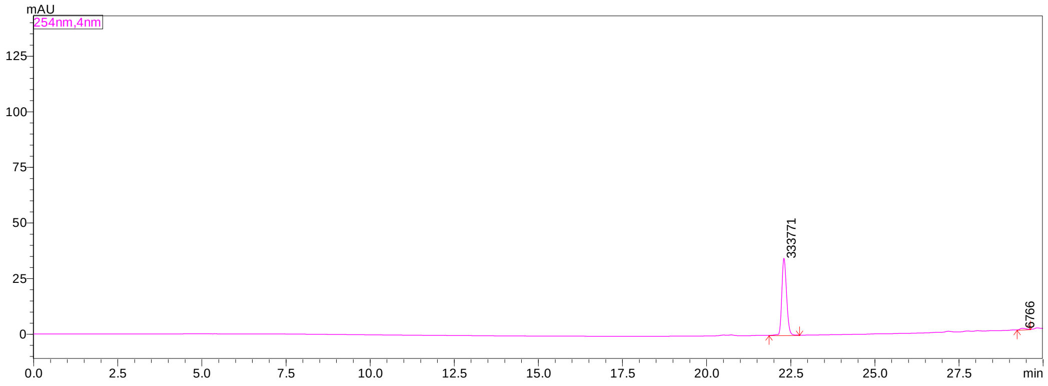

Figure S44. HPLC chromatogram of compound **11**.

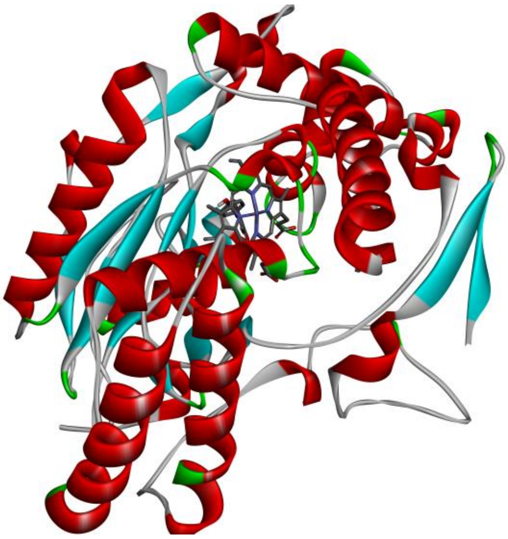

Figure S45. Overall view of Compound **1** docked with the iNOS.

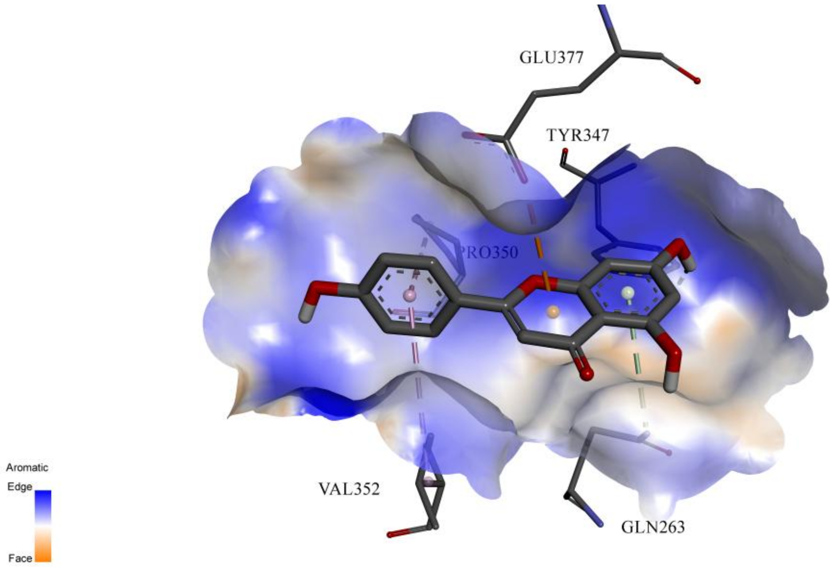

Figure S46. Enlarged view of Compound **1** docked with the iNOS.

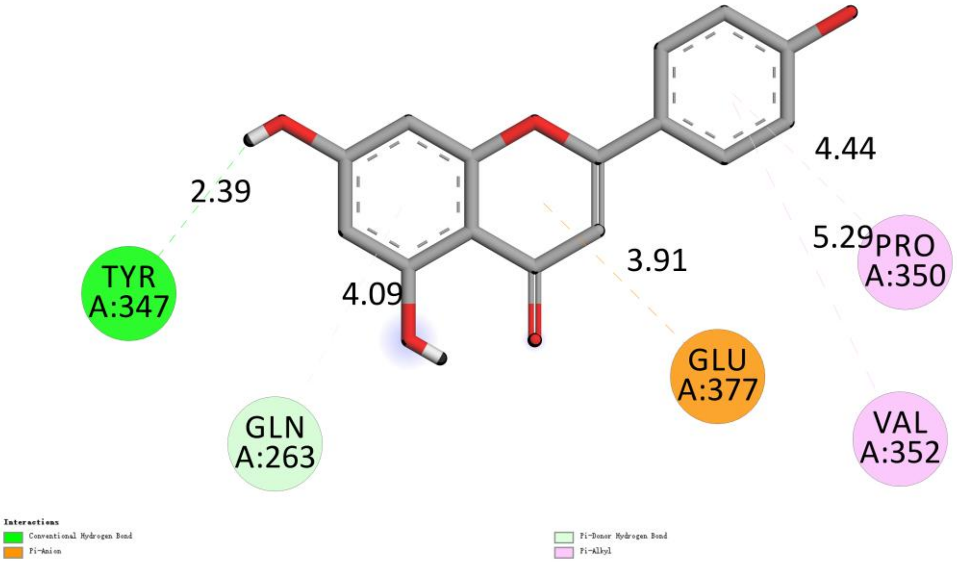

Figure S47. 2D diagram of Compound **1** docked with the iNOS.

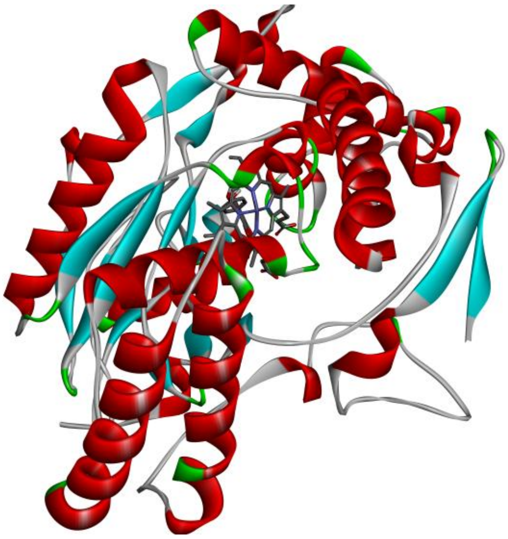

Figure S48. Overall view of Compound **2** docked with the iNOS.

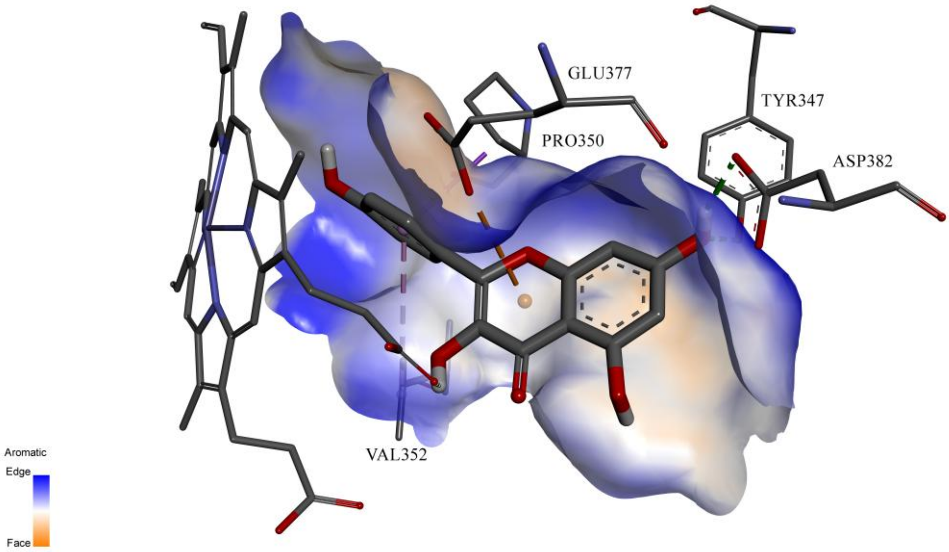

Figure S49. Enlarged view of Compound **2** docked with the iNOS.

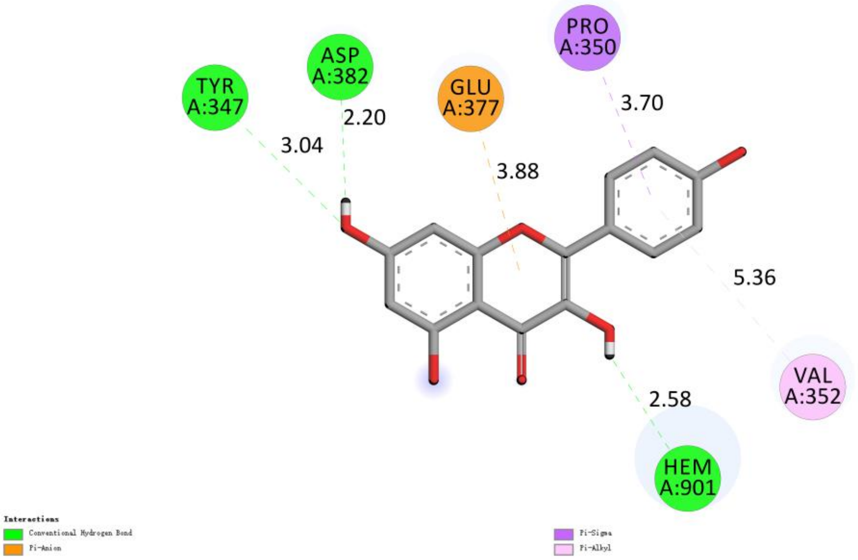

Figure S50. 2D diagram of Compound **2** docked with the iNOS.

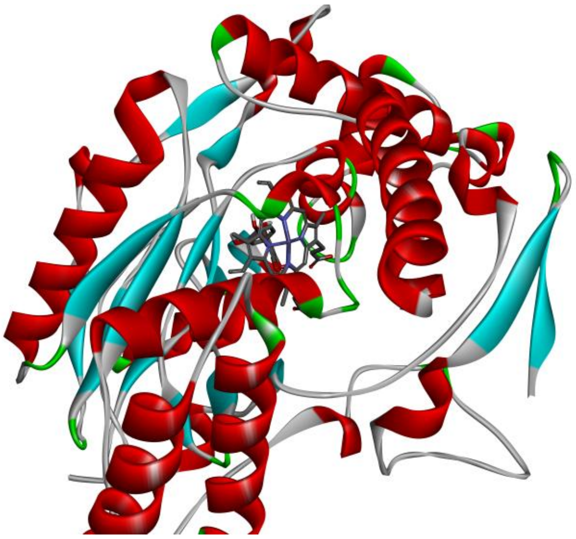

Figure S51. Overall view of Compound **3** docked with the iNOS.

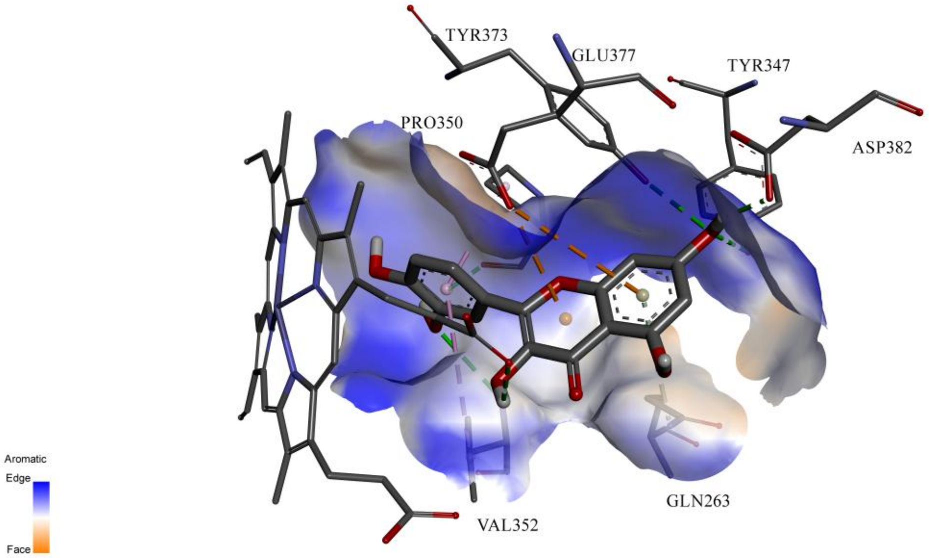

Figure S52. Enlarged view of Compound **3** docked with the iNOS.

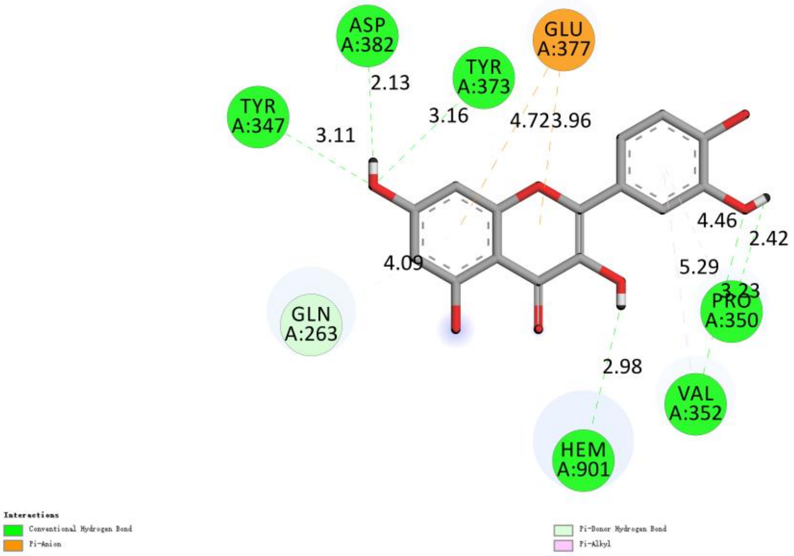

Figure S53. 2D diagram of Compound **3** docked with the iNOS.

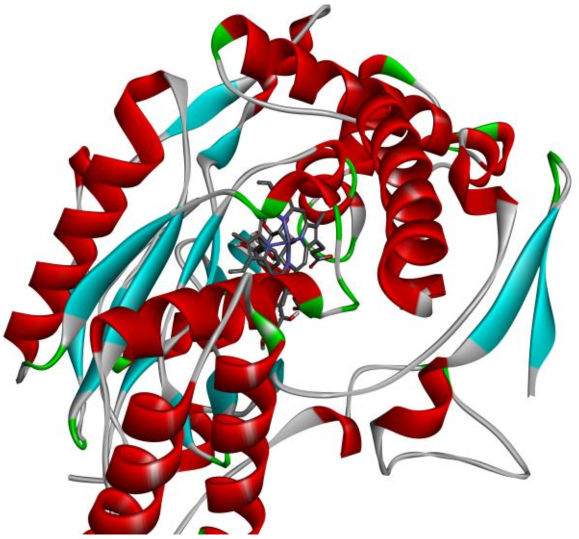

Figure S54. Overall view of Compound **4** docked with the iNOS.

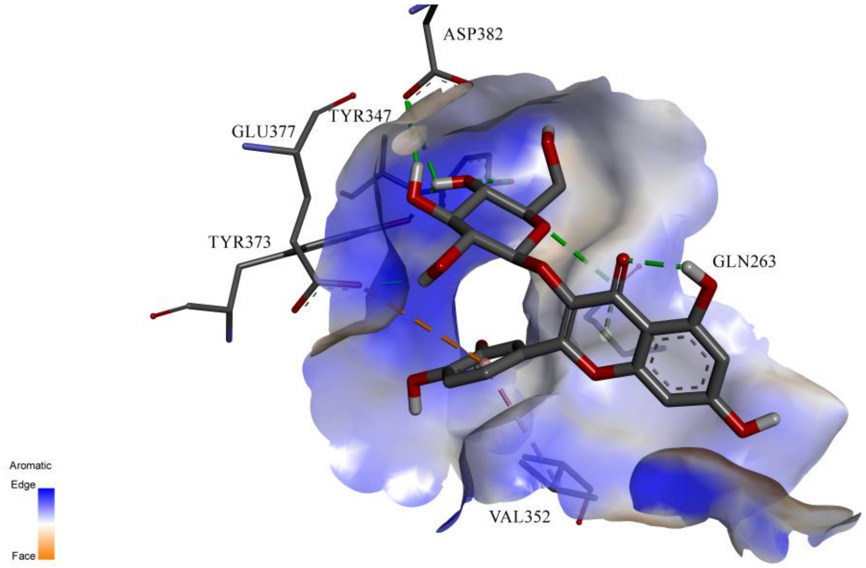

Figure S55. Enlarged view of Compound **4** docked with the iNOS.

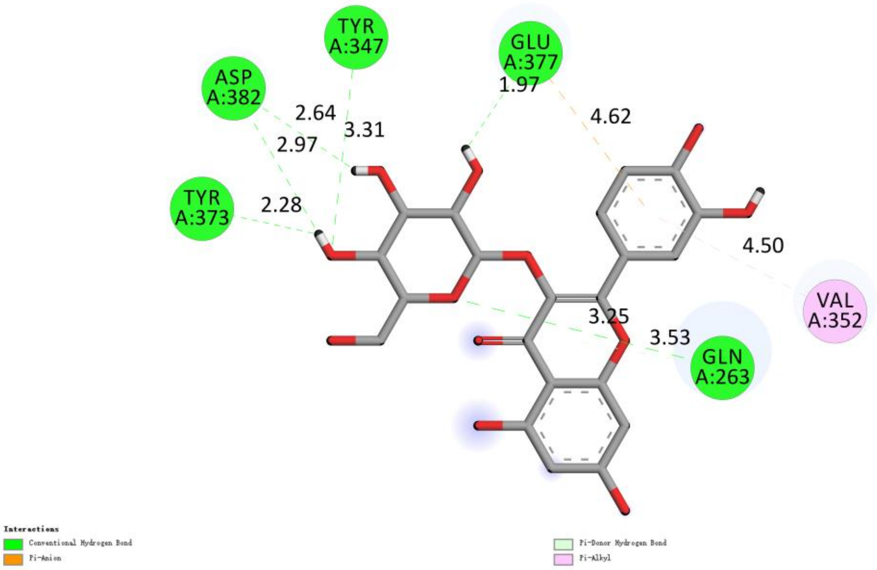

Figure S56. 2D diagram of Compound **4** docked with the iNOS.

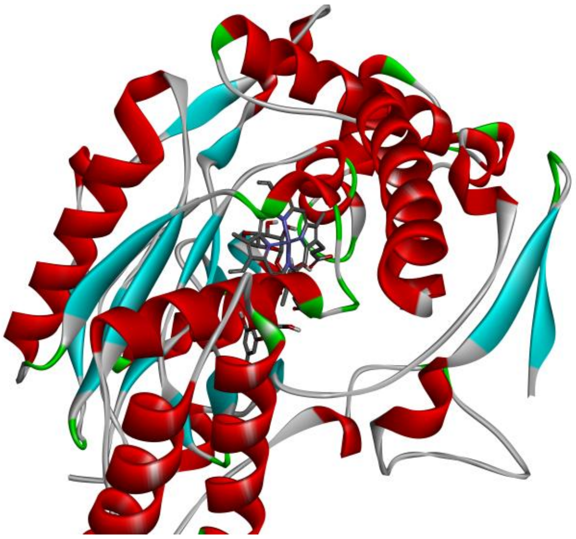

Figure S57. Overall view of Compound **5** docked with the iNOS.

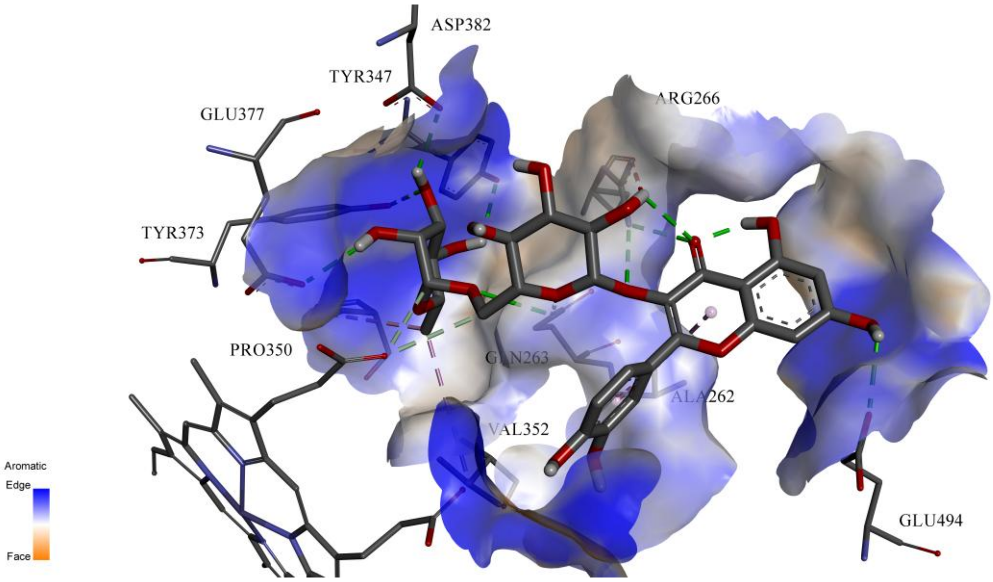

Figure S58. Enlarged view of Compound **5** docked with the iNOS.

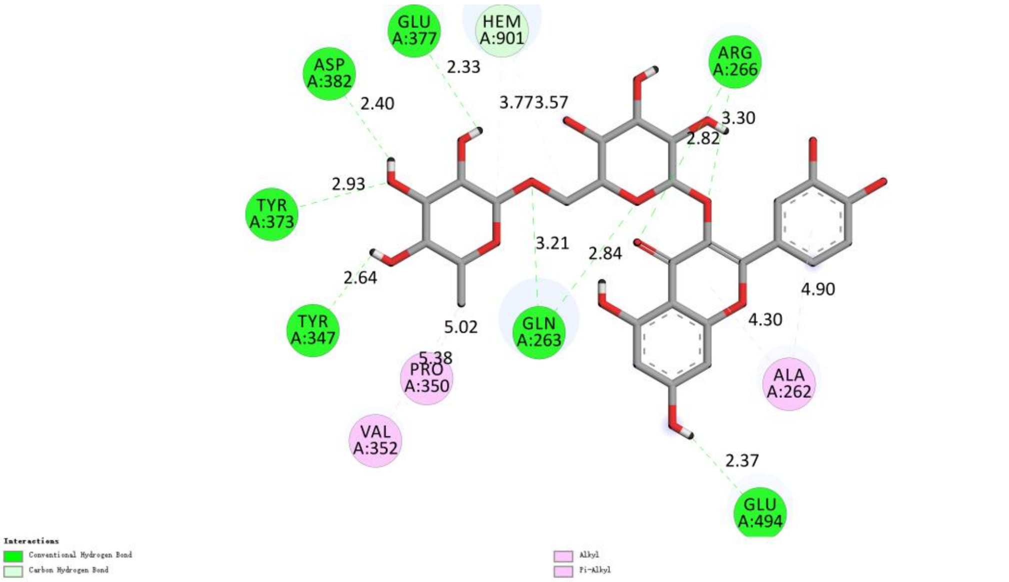

Figure S59. 2D diagram of Compound **5** docked with the iNOS.

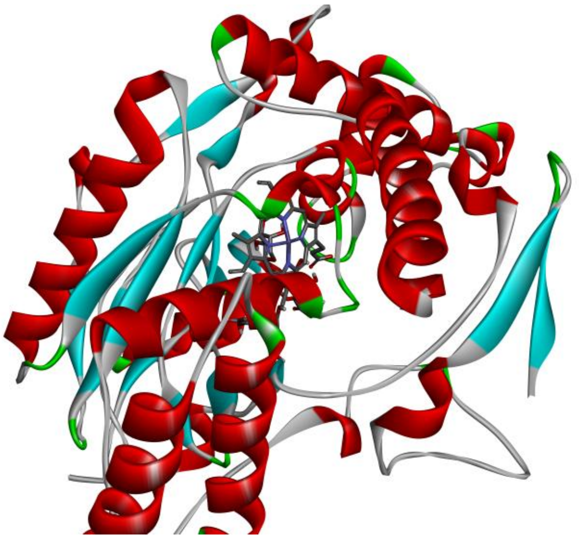

Figure S60. Overall view of Compound **6** docked with the iNOS.

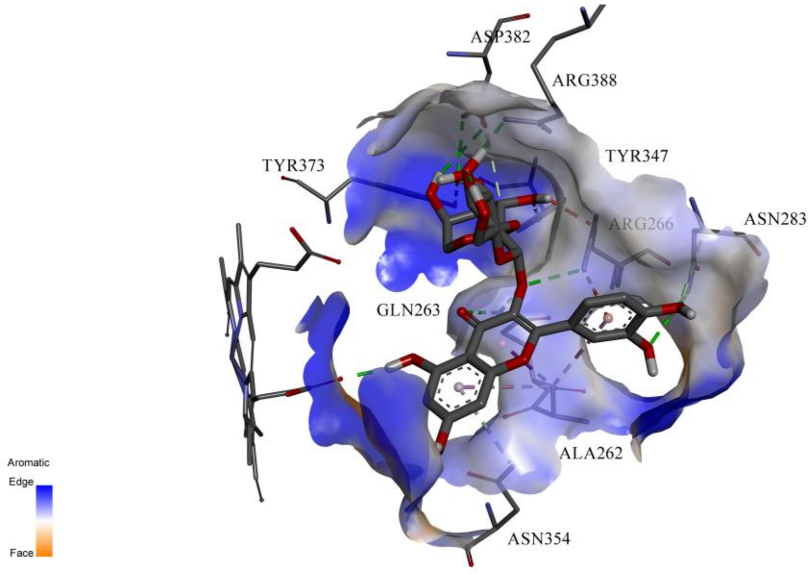

Figure S61. Enlarged view of Compound **6** docked with the iNOS.

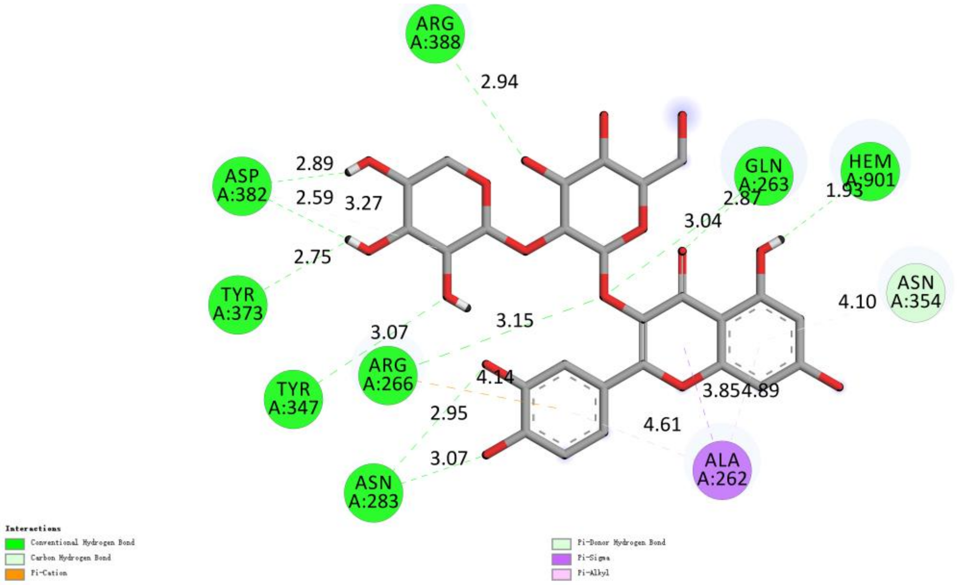

Figure S62. 2D diagram of Compound **6** docked with the iNOS.

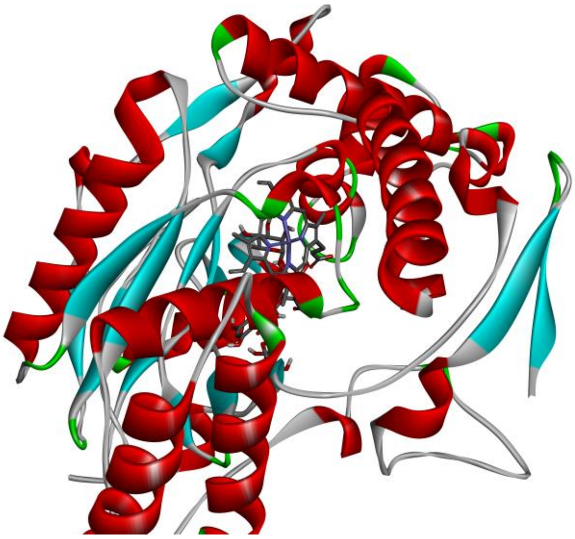

Figure S63. Overall view of Compound **7** docked with the iNOS.

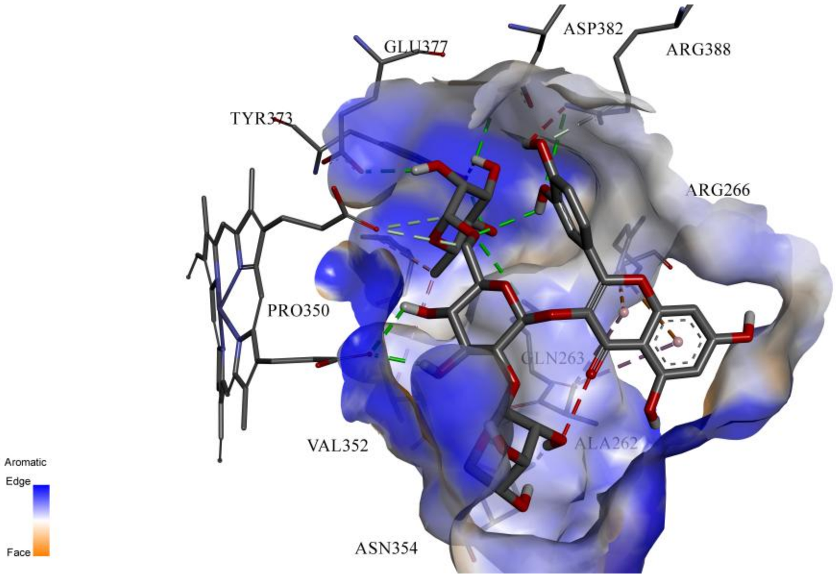

Figure S64. Enlarged view of Compound **7** docked with the iNOS.

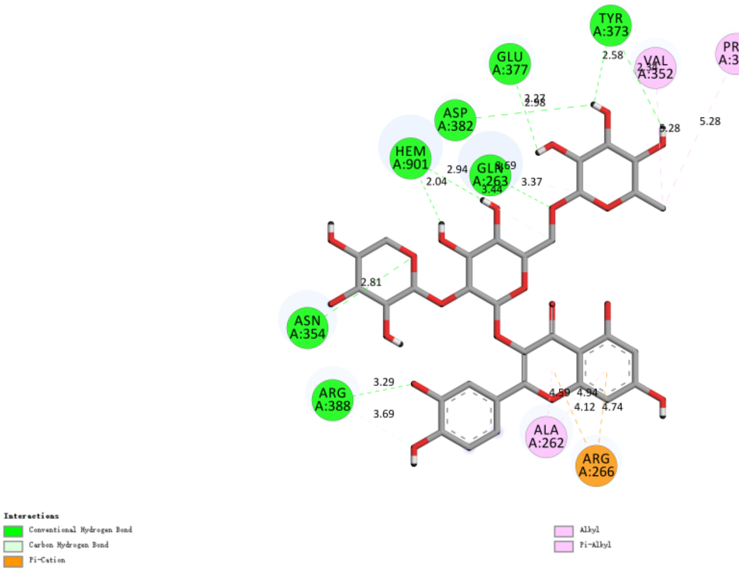

Figure S65. 2D diagram of Compound **7** docked with the iNOS.

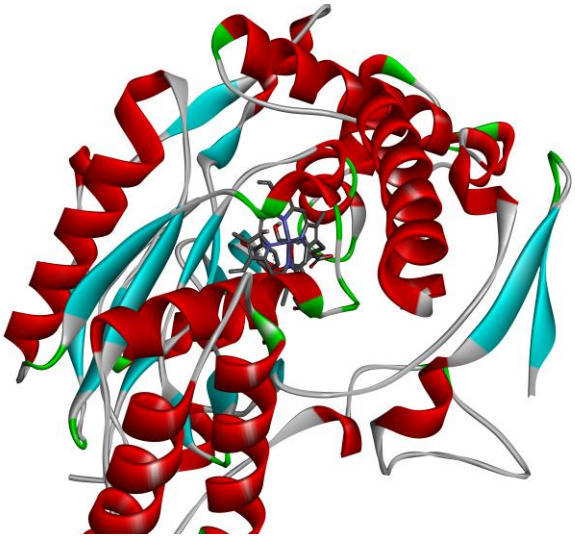

Figure S66. Overall view of Compound **8** docked with the iNOS.

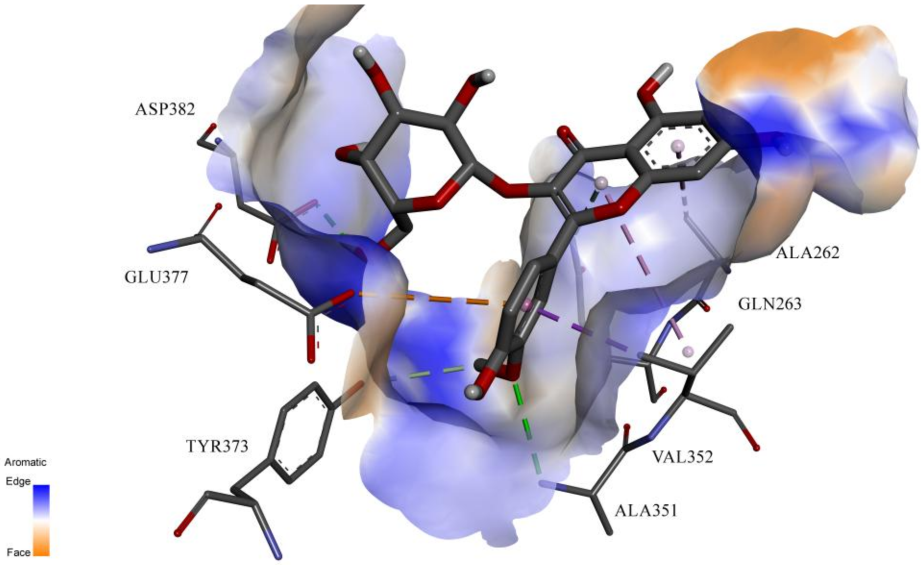

Figure S67. Enlarged view of Compound **8** docked with the iNOS.

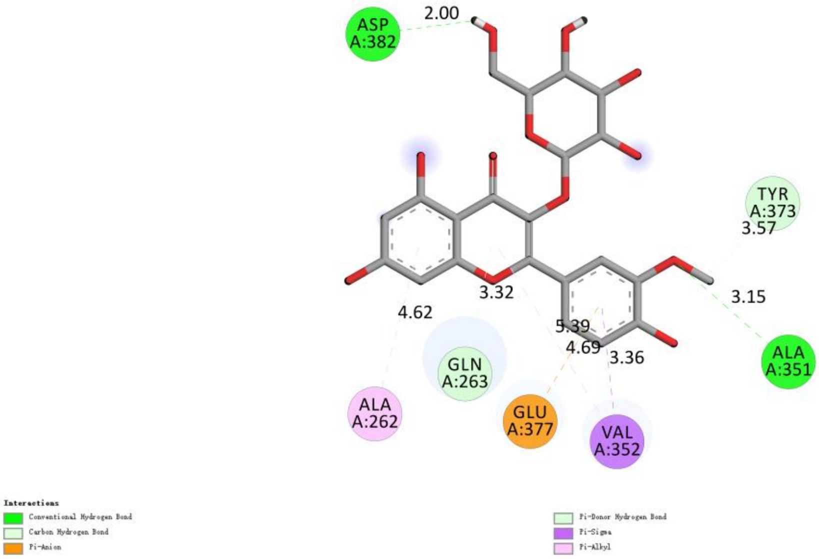

Figure S68. 2D diagram of Compound **8** docked with the iNOS.

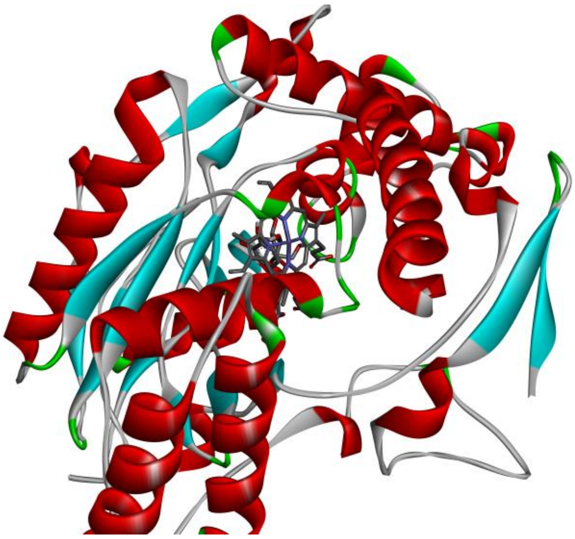

Figure S69. Overall view of Compound **9** docked with the iNOS.

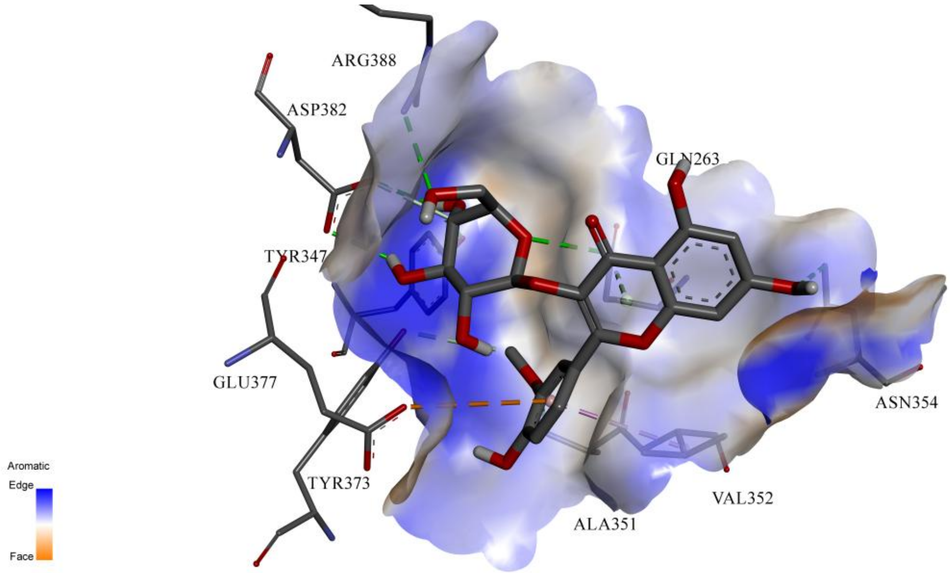

Figure S70. Enlarged view of Compound **9** docked with the iNOS.

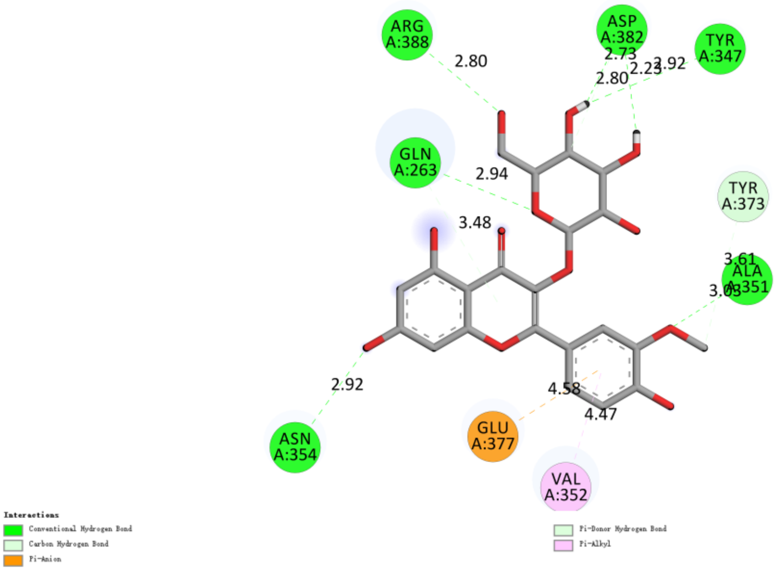

Figure S71. 2D diagram of Compound **9** docked with the iNOS.

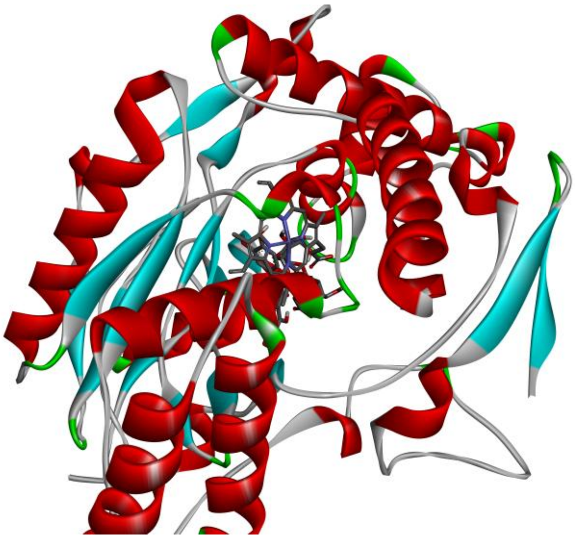

Figure S72. Overall view of Compound **10** docked with the iNOS.

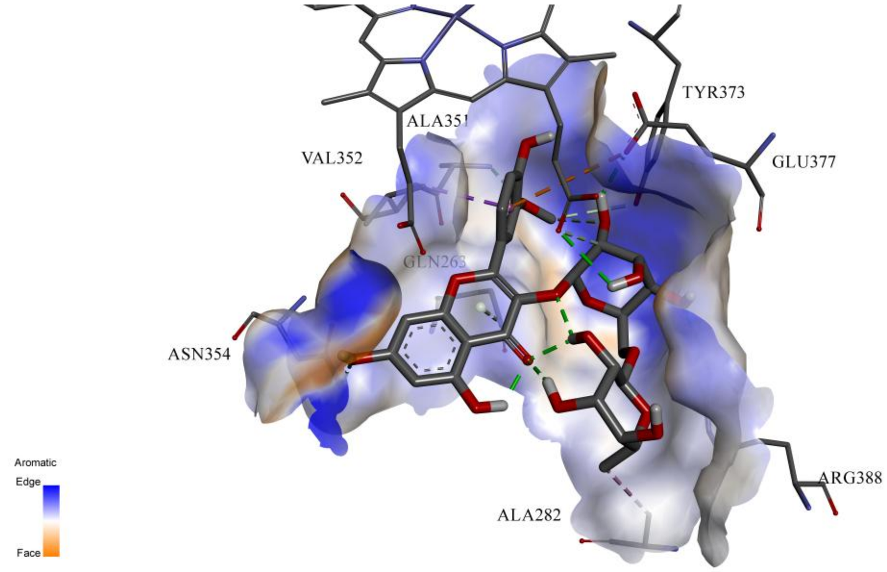

Figure S73. Enlarged view of Compound **10** docked with the iNOS.

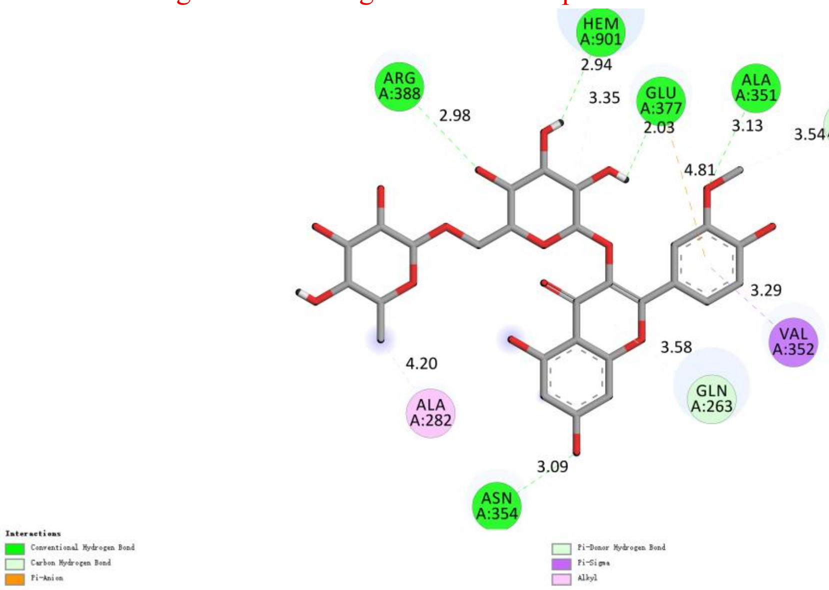

Figure S74. 2D diagram of Compound **10** docked with the iNOS.

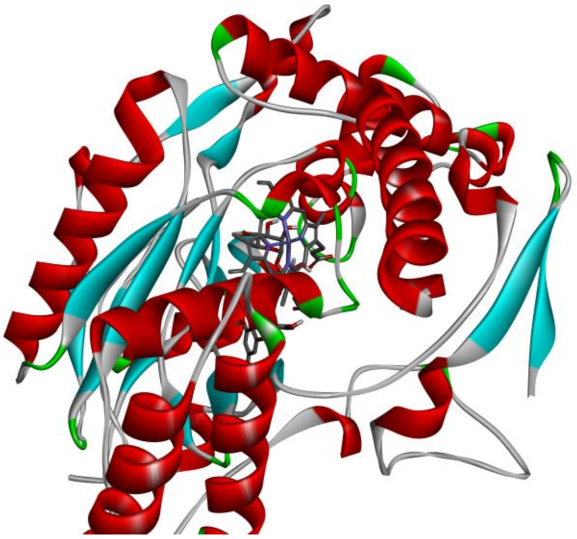

Figure S75. Overall view of Compound **11** docked with the iNOS.

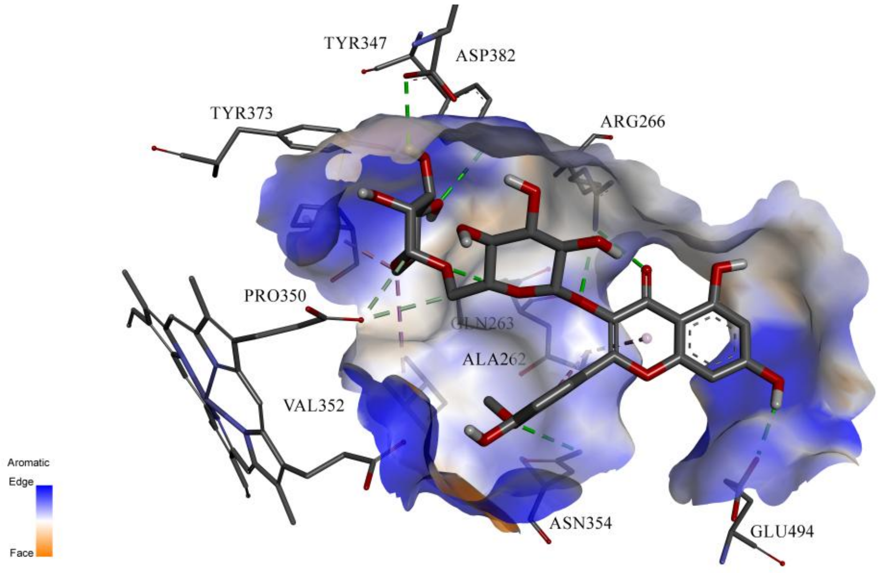

Figure S76. Enlarged view of Compound **11** docked with the iNOS.

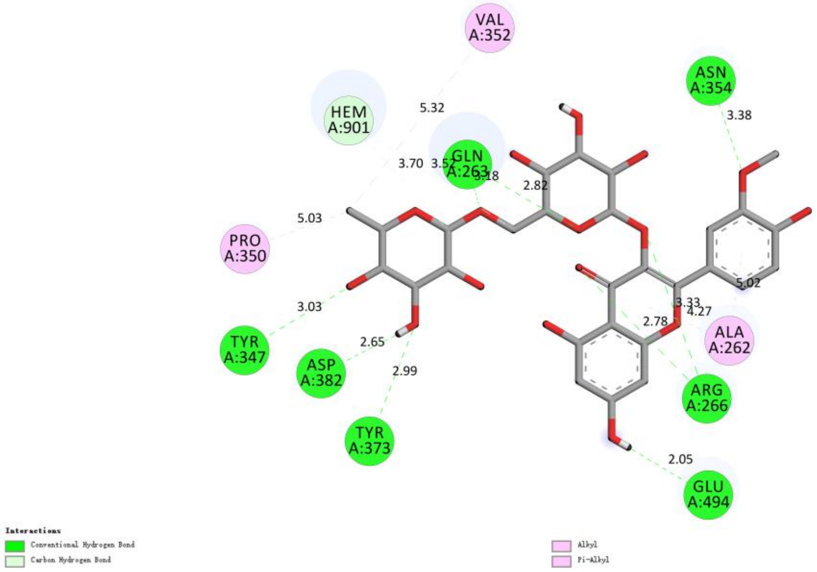

Figure S77. 2D diagram of Compound **11** docked with the iNOS.

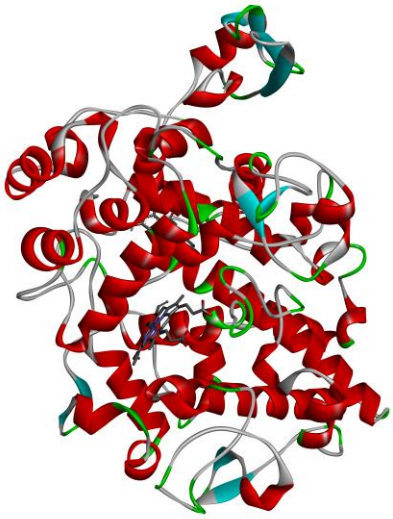

Figure S78. Overall view of Compound **1** docked with the COX-2.

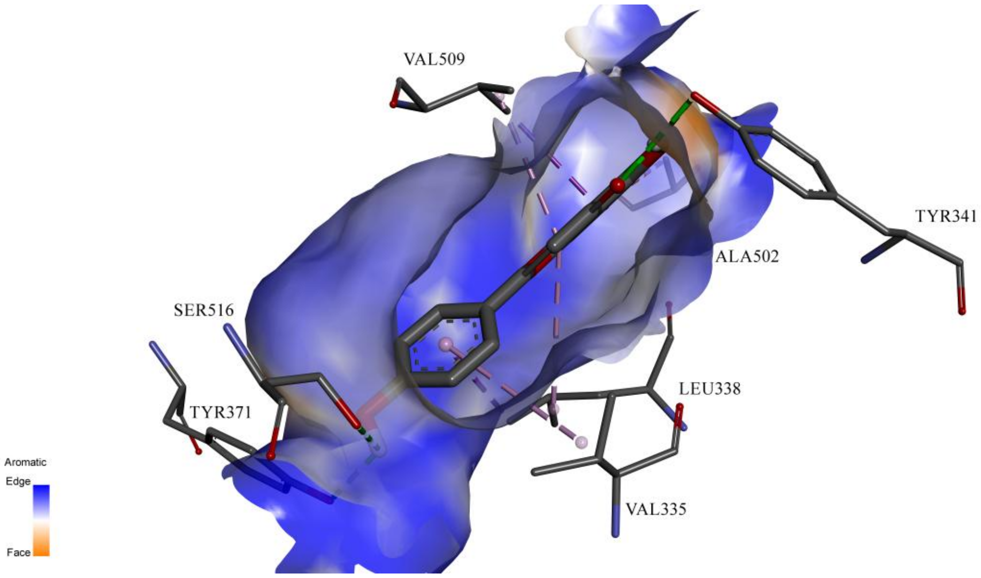

Figure S79. Enlarged view of Compound **1** docked with the COX-2.

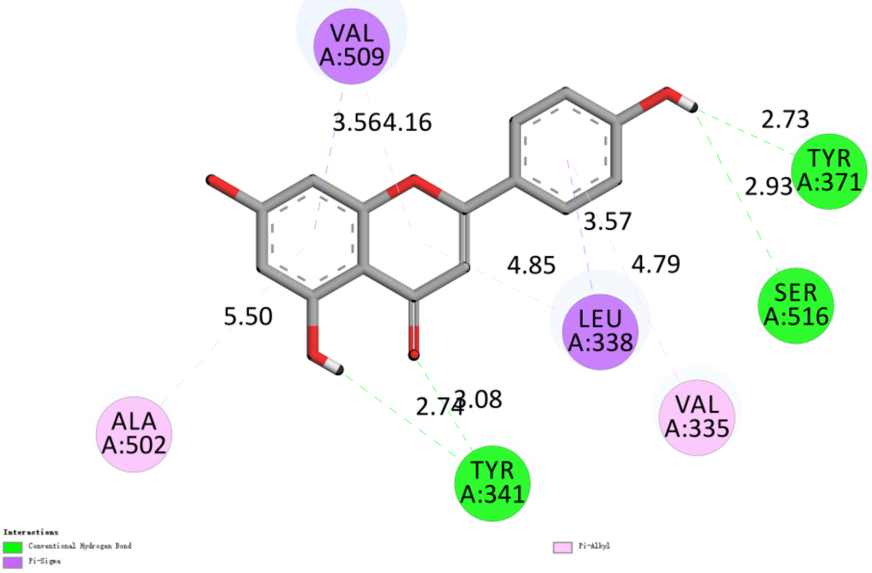

Figure S80. 2D diagram of Compound **1** docked with the COX-2.

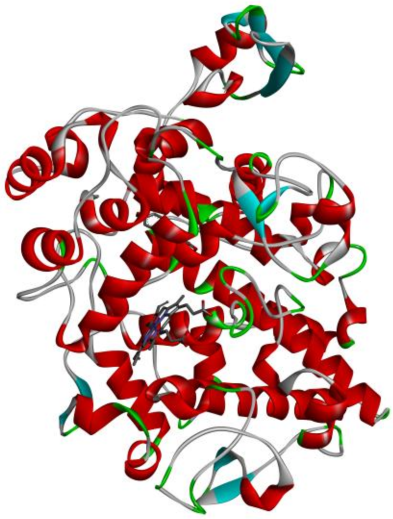

Figure S81. Overall view of Compound **2** docked with the COX-2.

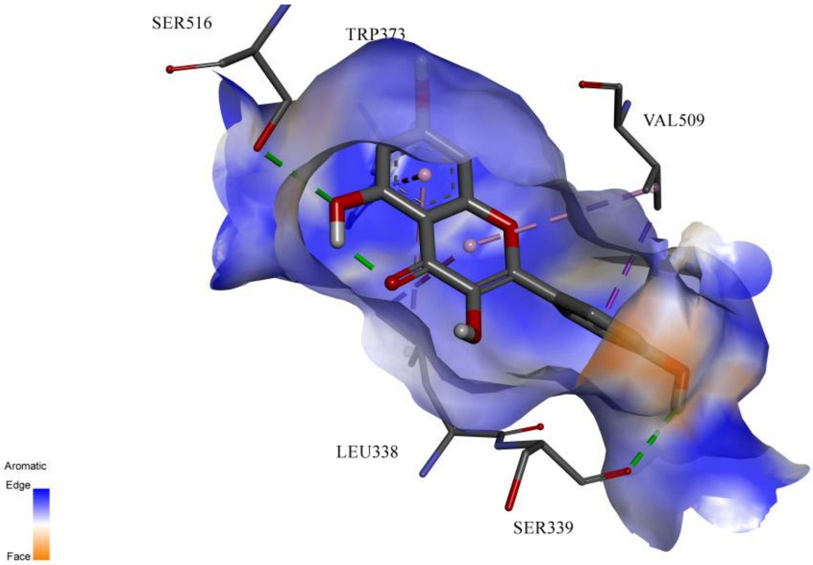

Figure S82. Enlarged view of Compound **2** docked with the COX-2.

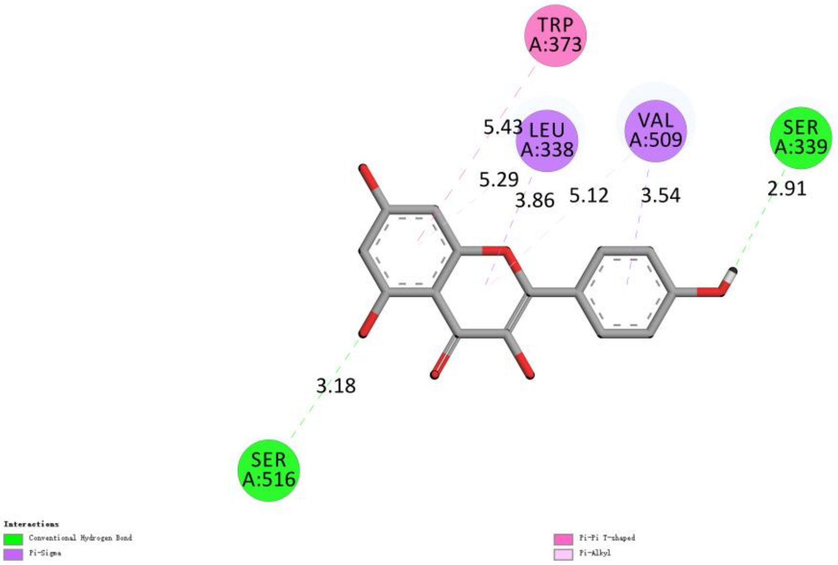

Figure S83. 2D diagram of Compound **2** docked with the COX-2.

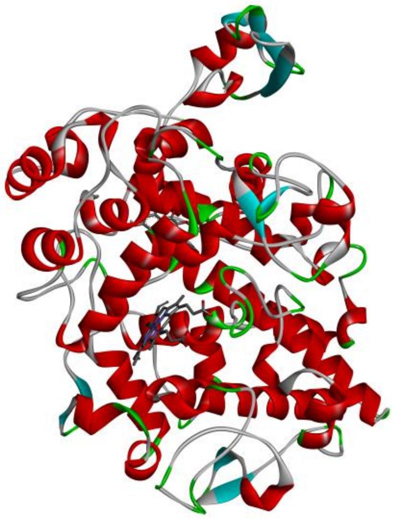

Figure S84. Overall view of Compound **3** docked with the COX-2.

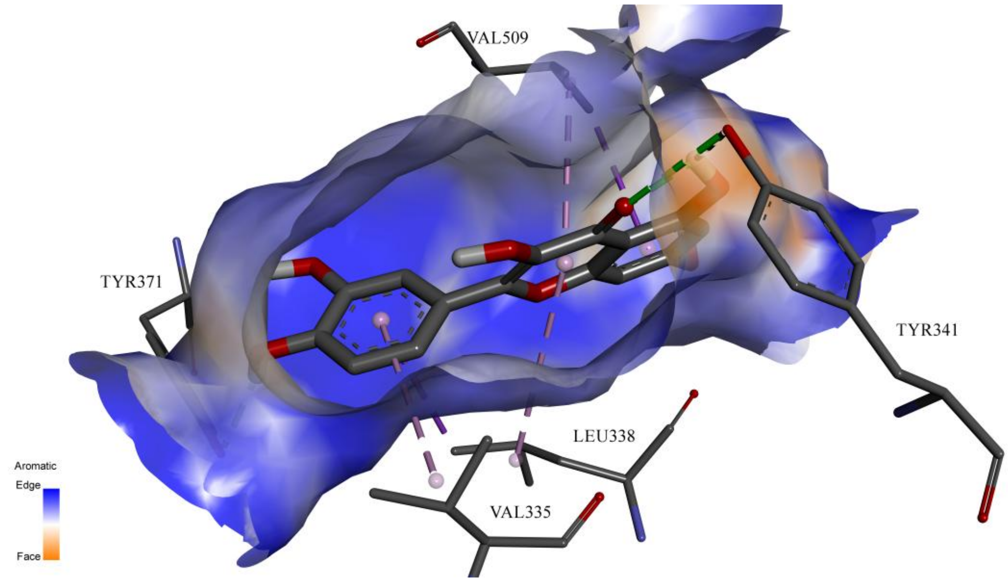

Figure S85. Enlarged view of Compound **3** docked with the COX-2.

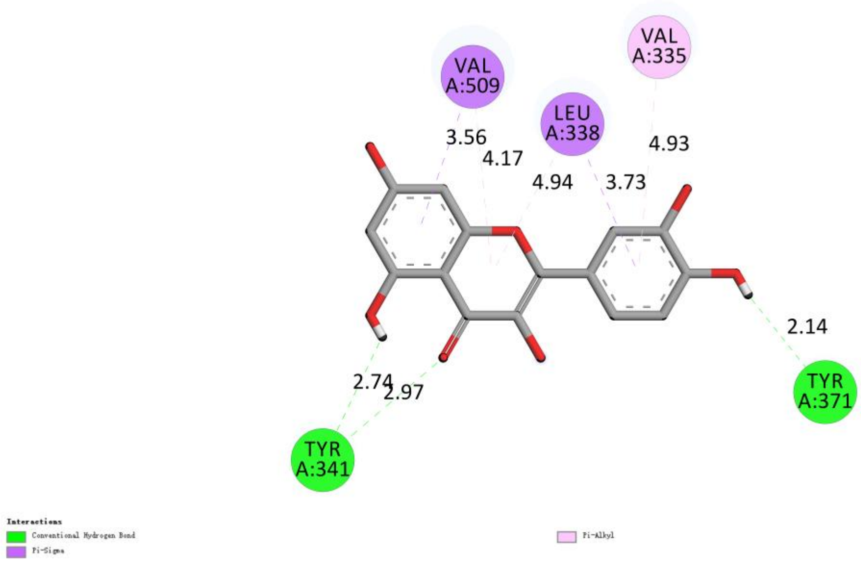

Figure S86. 2D diagram of Compound **3** docked with the COX-2.

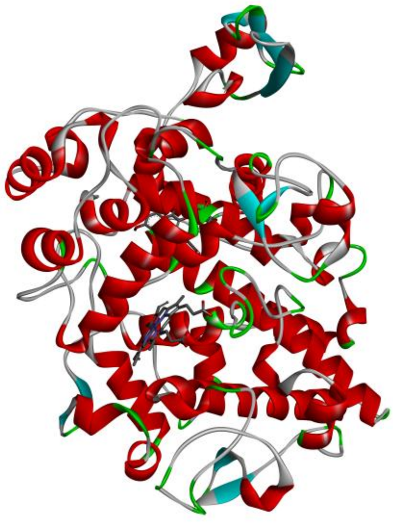

Figure S87. Overall view of Compound **4** docked with the COX-2.

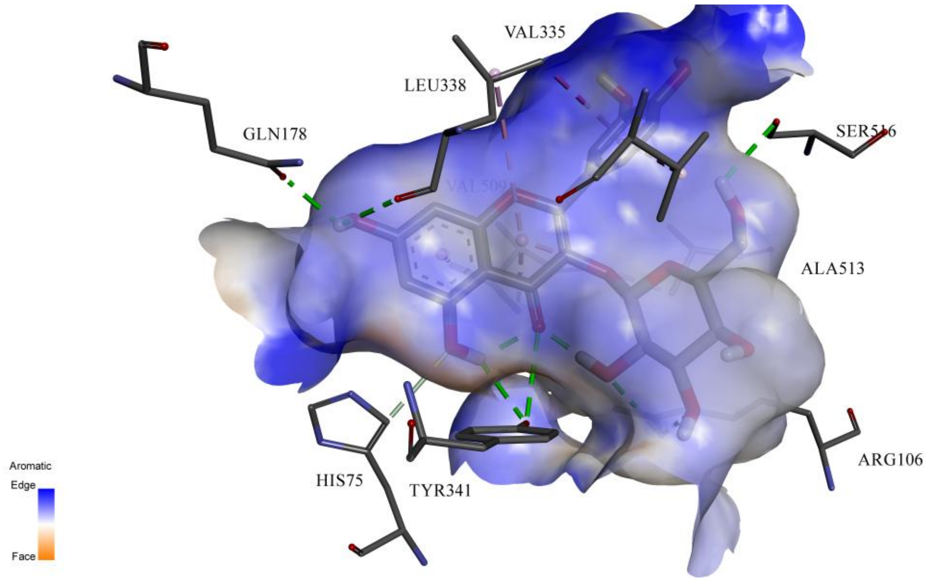

Figure S88. Enlarged view of Compound **4** docked with the COX-2.

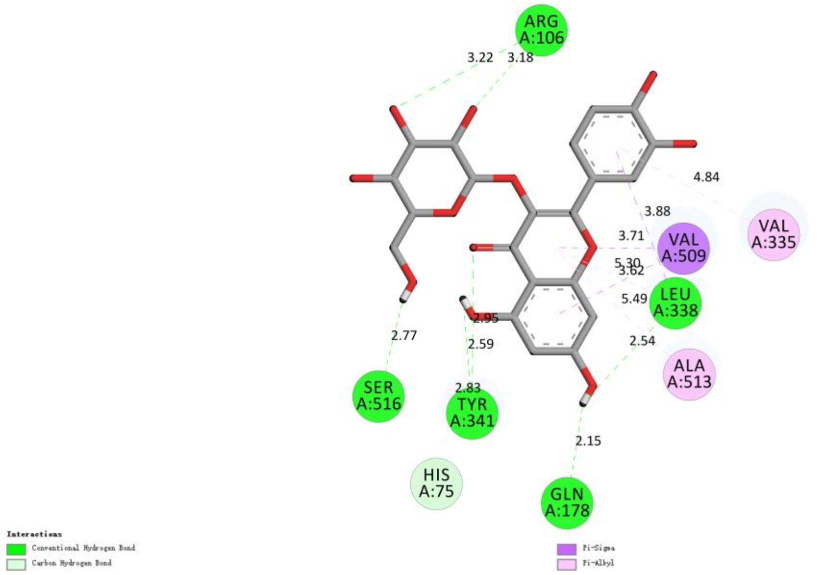

Figure S89. 2D diagram of Compound **4** docked with the COX-2.

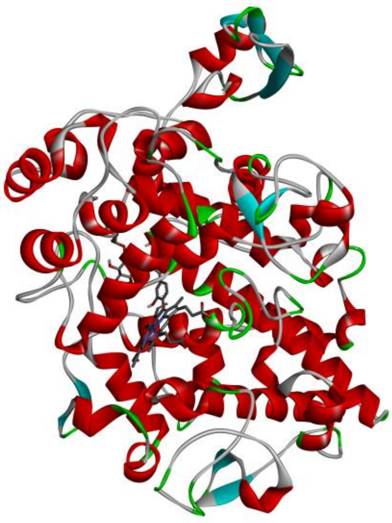

Figure S90. Overall view of Compound **5** docked with the COX-2.

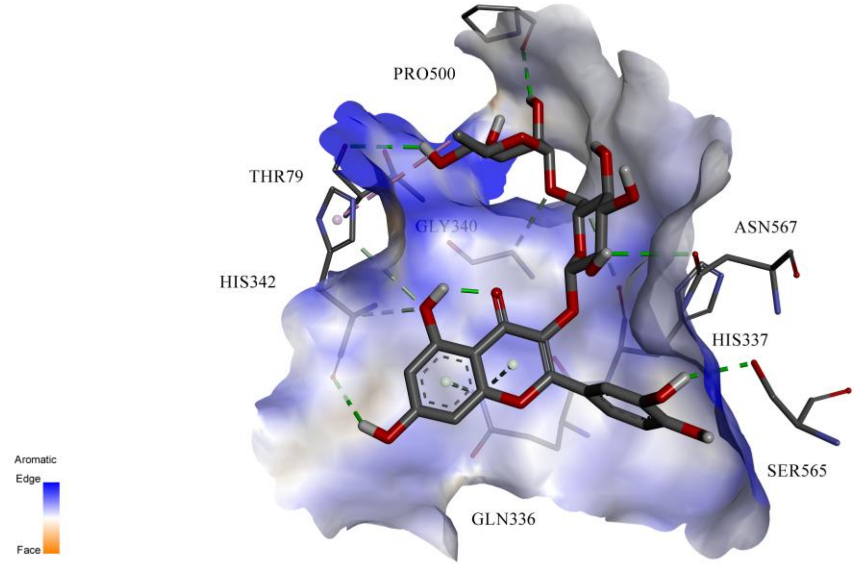

Figure S91. Enlarged view of Compound **5** docked with the COX-2.

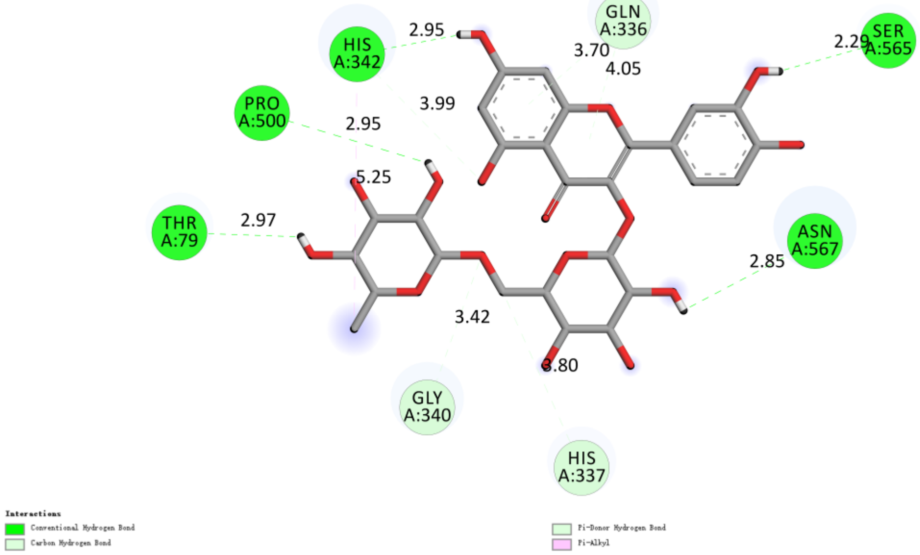

Figure S92. 2D diagram of Compound **5** docked with the COX-2.

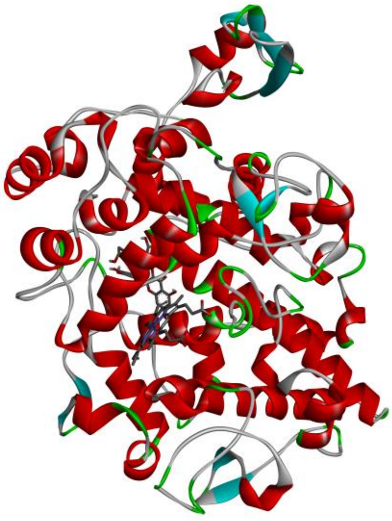

Figure S93. Overall view of Compound **6** docked with the COX-2.

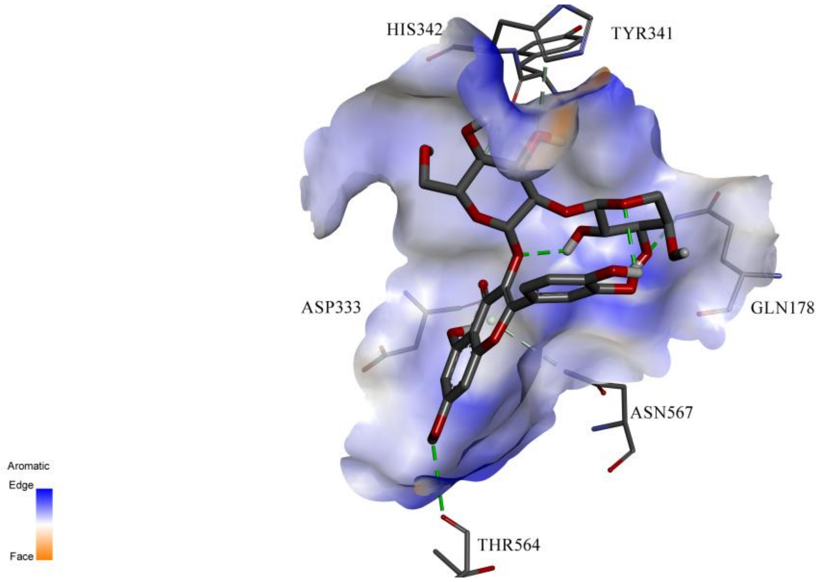

Figure S94. Enlarged view of Compound **6** docked with the COX-2.

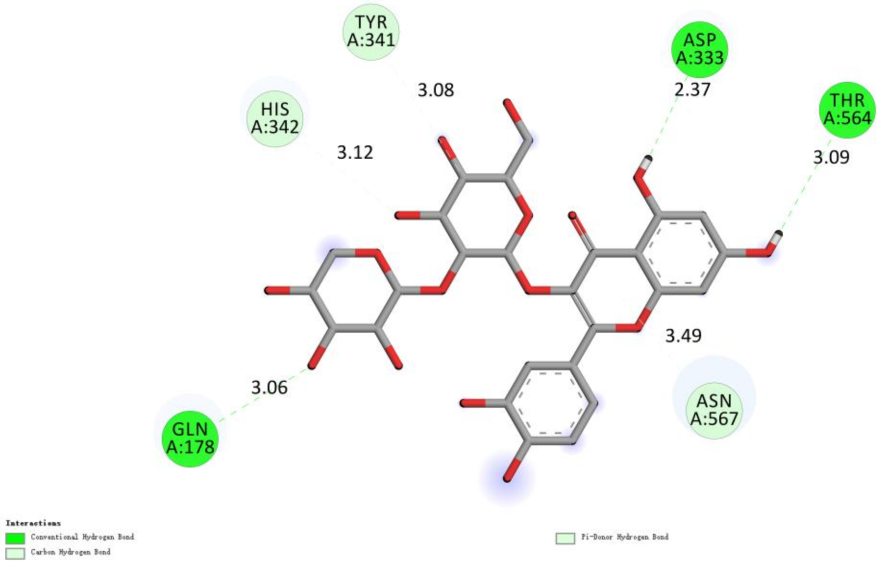

Figure S95. 2D diagram of Compound **6** docked with the COX-2.

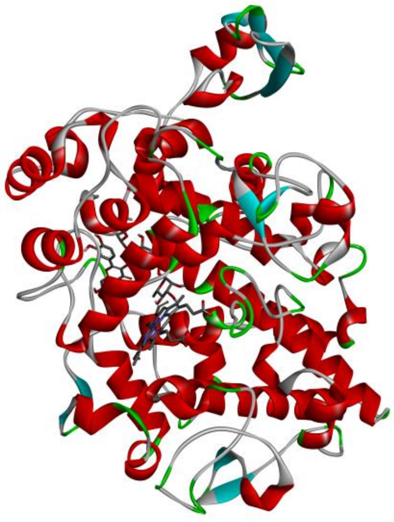

Figure S96. Overall view of Compound **7** docked with the COX-2.

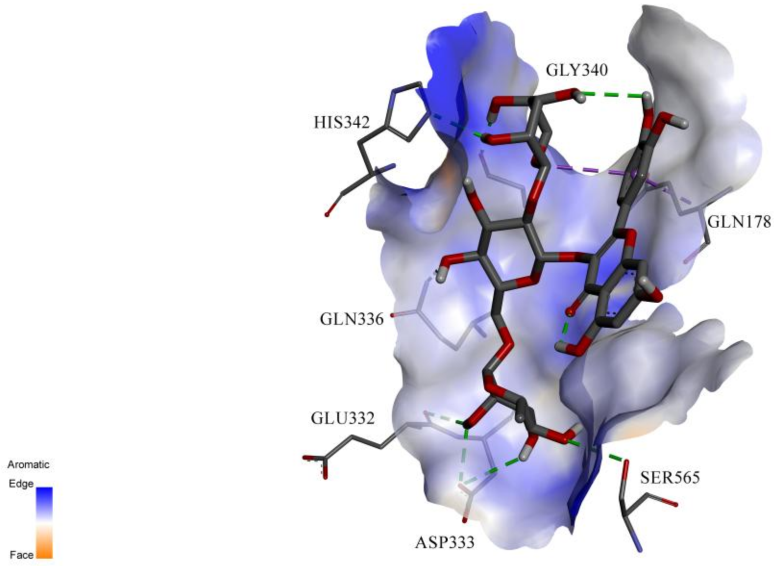

Figure S97. Enlarged view of Compound **7** docked with the COX-2.

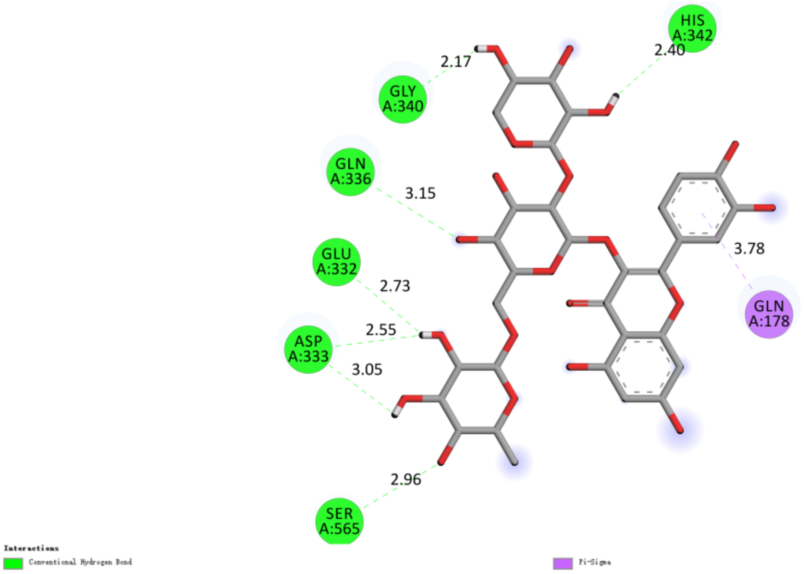

Figure S98. 2D diagram of Compound **7** docked with the COX-2.

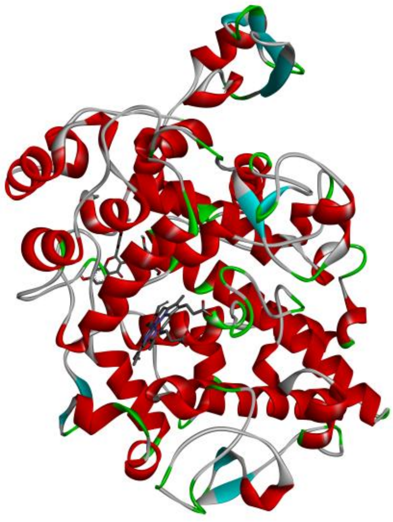

Figure S99. Overall view of Compound **8** docked with the COX-2.

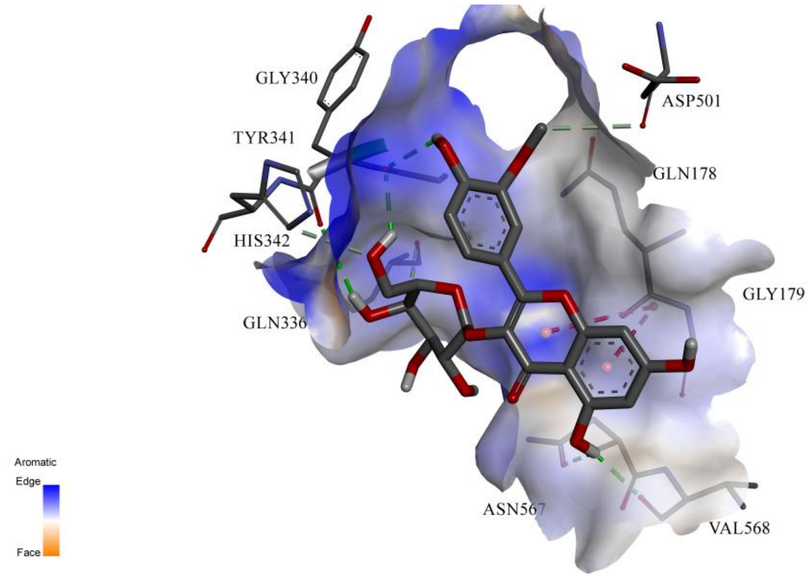

Figure S100. Enlarged view of Compound **8** docked with the COX-2.

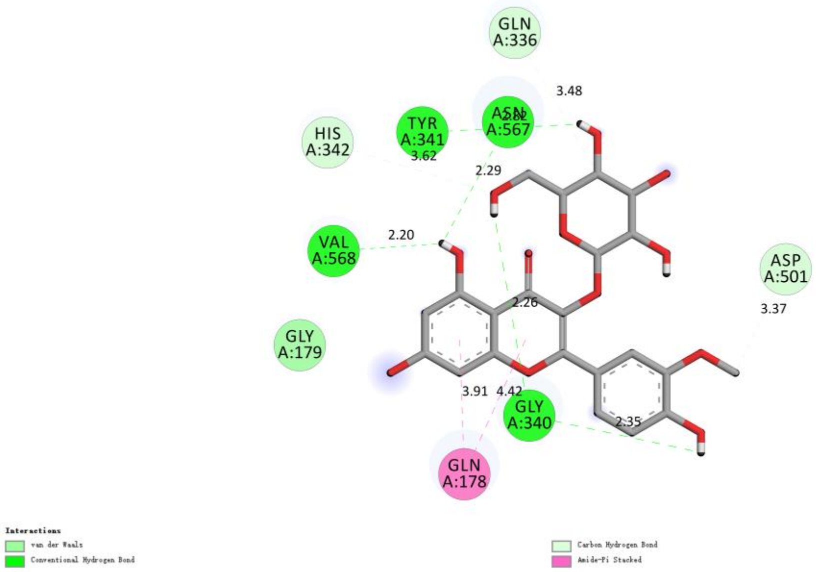

Figure S101. 2D diagram of Compound **8** docked with the COX-2.

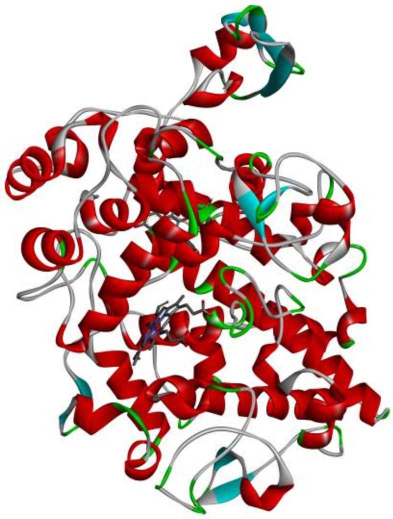

Figure S102. Overall view of Compound **9** docked with the COX-2.

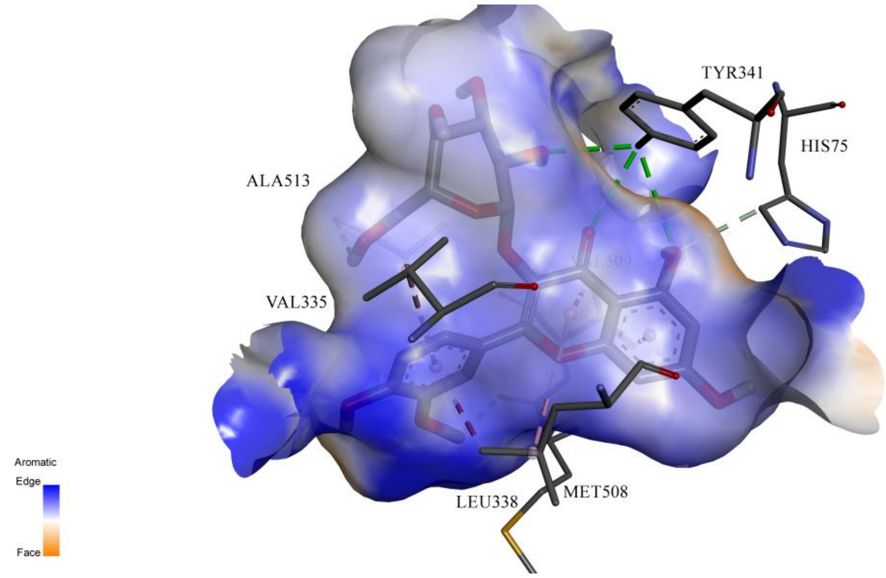

Figure S103. Enlarged view of Compound **9** docked with the COX-2.

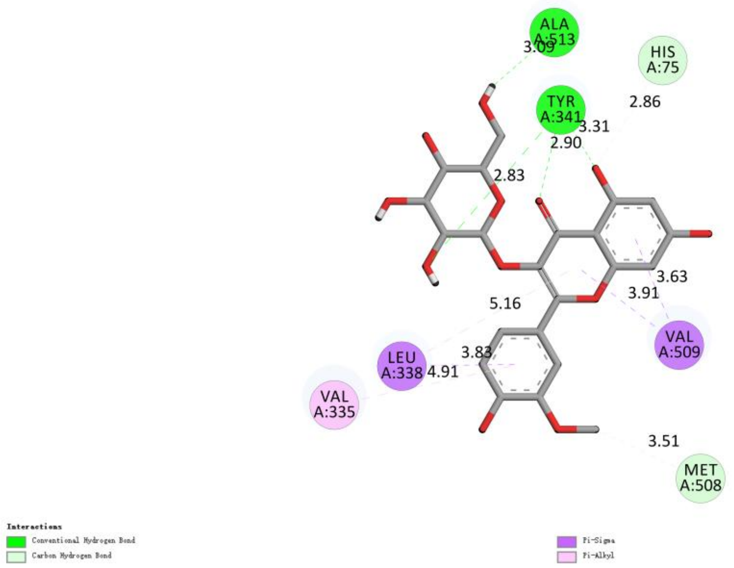

Figure S104. 2D diagram of Compound **9** docked with the COX-2.

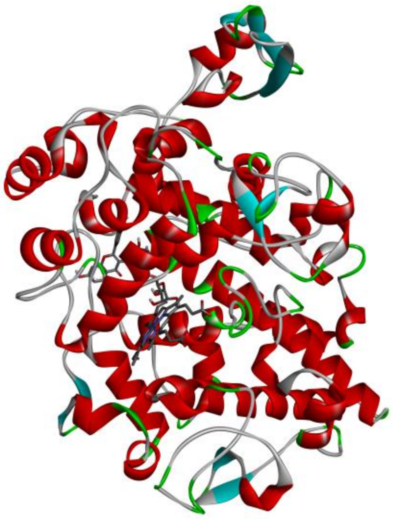

Figure S105. Overall view of Compound **10** docked with the COX-2.

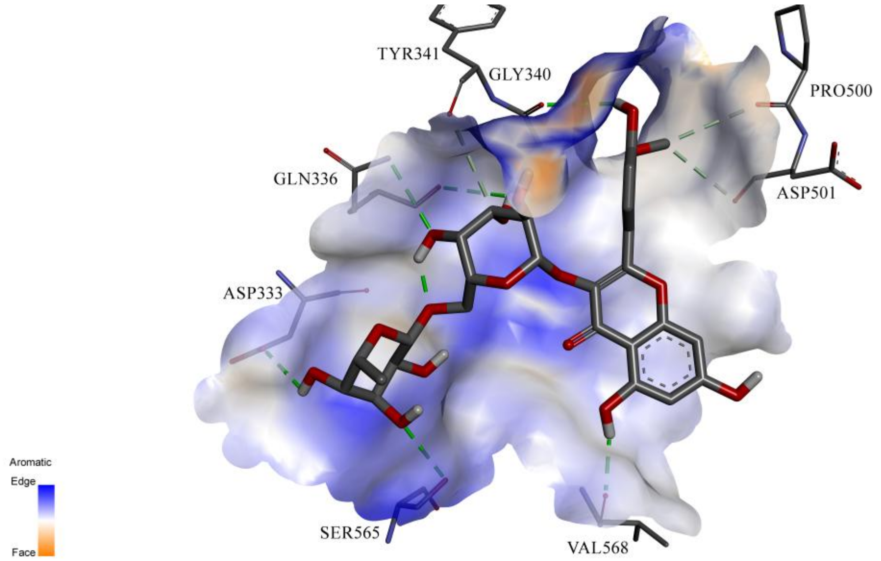

Figure S106. Enlarged view of Compound **10** docked with the COX-2.

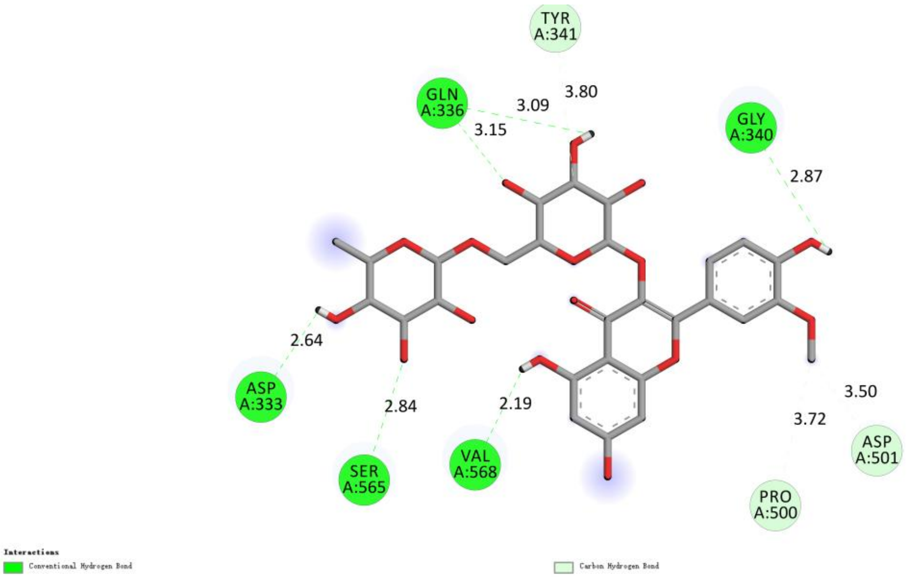

Figure S107. 2D diagram of Compound **10** docked with the COX-2.

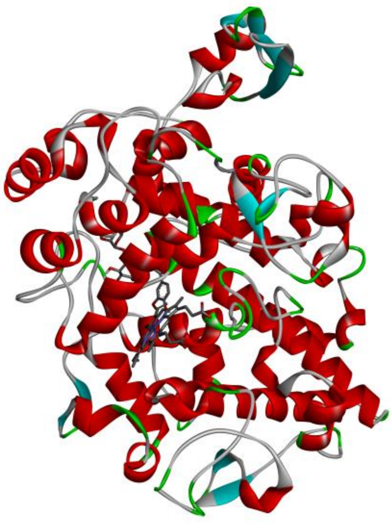

Figure S108. Overall view of Compound **11** docked with the COX-2.

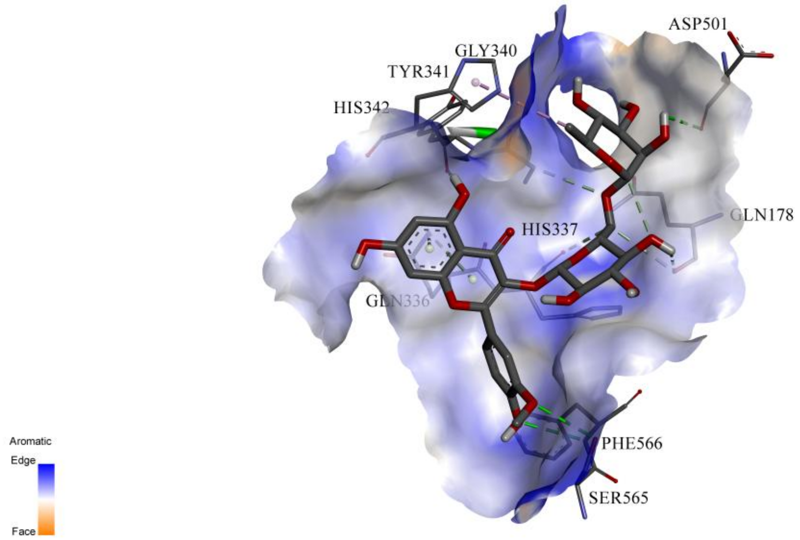

Figure S109. Enlarged view of Compound **11** docked with the COX-2.

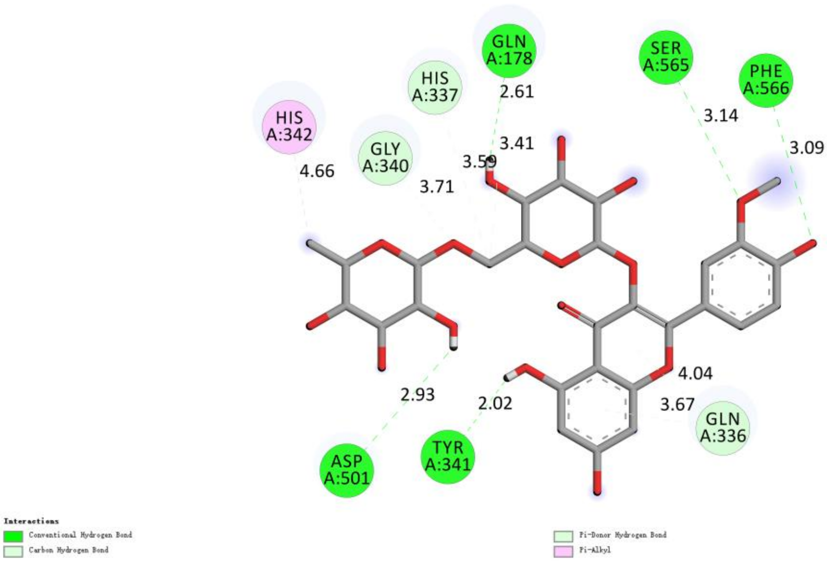

Figure S110. 2D diagram of Compound **11** docked with the COX-2.

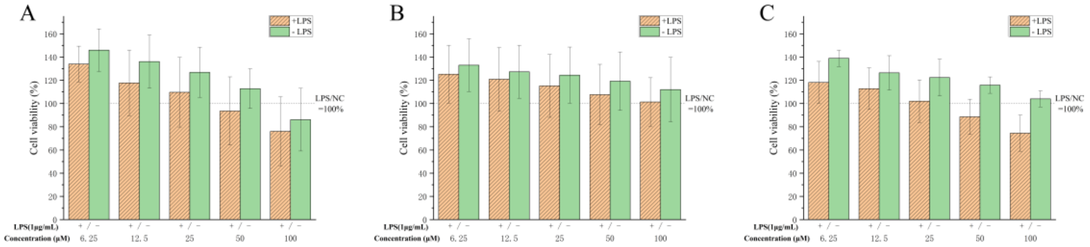

Figure S111. Cell viability after treatment with compounds **1**(A), **2**(B), and **8**(C) at different concentrations.
